# Stability-Indicating Assay of Novel 5-(Hydroxamic acid)methyl Oxazolidinones with 5-Lipooxygenase Inhibitory Activity

**DOI:** 10.3390/ph19010069

**Published:** 2025-12-29

**Authors:** Hessa M. Al-Mutairi, Oludotun A. Phillips, Naser F. Al-Tannak

**Affiliations:** 1 College of Graduate Studies, Kuwait University, P.O. Box 12345, Safat, Kuwait City 13060, Kuwait; 2 Department of Pharmaceutical Chemistry, College of Pharmacy, Health Science Center, Kuwait University, P.O. Box 24923, Safat, Kuwait City 13110, Kuwait; oludotun.phillips@ku.edu.kw

**Keywords:** oxazolidinone, oxazolidinone hydroxamic acid derivatives, stability indicating assay, ultra-high-performance liquid chromatography, liquid chromatography–quadrupole time-of-flight mass spectrometry

## Abstract

**Background**: Oxazolidinone derivatives are a novel class of synthetic antibacterial agents, characterized by a five-membered heterocyclic ring containing oxygen and nitrogen and a carbonyl functionality at position 2. This pharmacophore is responsible not only for antibacterial activity but also for a variety of other biological activities, including anticancer activity, anticoagulant activity, and several others. A series of novel oxazolidinone derivatives containing a hydroxamic acid moiety were synthesized in our laboratories and identified as potent inhibitors of the enzyme 5-lipoxygenase (5-LO), a key enzyme involved in the biosynthesis of leukotrienes (LTs). LTs are proinflammatory mediators implicated in allergic and inflammatory diseases. Currently, zileuton is the only FDA-approved 5-LO inhibitor, emphasizing the need to develop new agents for the treatment of such diseases. This project aims to develop validated stability-indicating analytical methods for the four most potent novel 5-(hydroxamic acid)methyl oxazolidinone derivatives (PH-211, PH-247, PH-249, and PH-251). **Methods**: The compounds were analyzed using Waters Acquity Ultra-High-Performance Liquid Chromatography (UHPLC-UV) with an ultraviolet detector to determine their stability in human plasma and under various forced degradation conditions, including acidic, basic, oxidative, and thermal conditions. Liquid chromatography–quadrupole time-of-flight mass spectrometry (LC-QToF-MS) was used to identify possible degradation products. **Results**: The compounds were found to be stable in human plasma and under thermal degradation conditions with high extraction recoveries (82–90%) but unstable in acidic, basic, and oxidative conditions. **Conclusions**: The findings show that the compounds are stable in biological conditions; they hold promise for the treatment of inflammatory and allergic diseases.

## 1. Introduction

**Figure 1:**
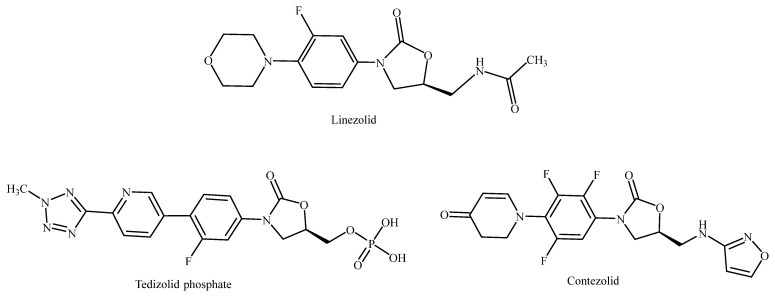
Chemical structure of oxazolidinone antibacterial agents.

The oxazolidinone scaffold is a remarkable structural motif in medicinal chemistry because of its favorable pharmacological properties. Numerous studies have shown that compounds with this core show a variety of biological activities, including antibacterial, anticancer, anticoagulant, antithyroid, enzyme inhibitory, and central nervous system effects, among others [1,2,3,4,5,6,7,8,9]. Oxazolidinone is a fully synthetic five-membered heterocyclic ring containing oxygen and nitrogen atoms and a carbonyl functional group at different positions within the ring. However, 2-oxazolidinone is the most investigated in drug discovery and is the focus of our study. The oxazolidinone class of antibacterial agents, exemplified by linezolid, tedizolid, which is available in its prodrug form tedizolid phosphate, and contezolid (Figure 1), represents a novel class of antibacterial agents showing high potency against multidrug-resistant (MDR) Gram-positive pathogenic bacterial strains and is active against methicillin-resistant *Staphylococcus aureus* (MRSA), vancomycin-resistant Enterococci (VRE), and penicillin-resistant *Streptococcus pneumoniae* (PRSP) [10]. Several other derivatives from this class have been discovered and are currently under clinical investigation to evaluate their safety and efficacy profiles [11]. Oxazolidinones inhibit bacterial protein biosynthesis through a distinct mechanism of action, which prevents cross-resistance with other protein synthesis inhibitors [12]. It was found that oxazolidinone inhibits the formation of the 70S initiation complex by binding only to the 50S subunit but having no affinity for the 30S subunit [12,13,14].

**Figure 2:**
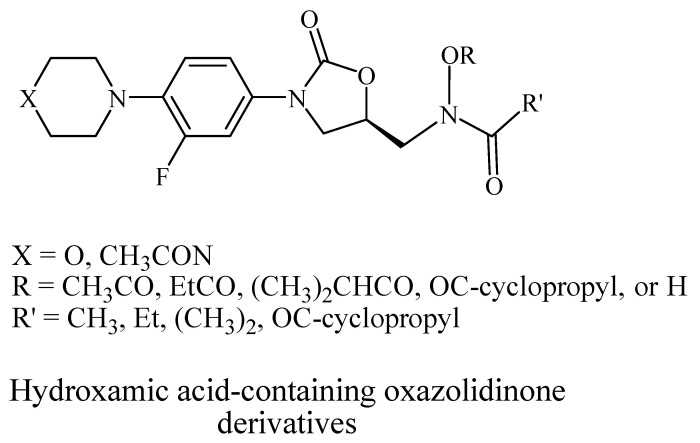
Chemical structures of hydroxamic acid-containing oxazolidinone derivatives.

Oxazolidinone derivatives have also demonstrated additional pharmacological properties of significance, and some are clinically used as anticoagulants and psychoactive agents [6]. Rivaroxaban is the first available oral anticoagulant featuring the oxazolidinone ring, which is a direct factor Xa inhibitor [6]. Monoamine oxidase (MAO) inhibitory activity was noticed with oxazolidinone antibacterial agents and was considered as a side effect of the treatment. However, this biological effect was useful for developing psychoactive agents, namely toloxatone, which is a reversible inhibitor of MAO-A clinically used as an antidepressant. Furthermore, oxazolidinone derivatives with varying structural features have been identified and investigated globally and in our laboratories for their anticancer and central nervous system activity [4,5,6,7,15]. Moreover, structural modifications conducted on the 2-oxazolidinone moiety yielded the novel oxazolidinone derivative PH-192 with anticonvulsant effects [8,9]. PH-192 demonstrated significant protection in mice and rat models against both chemically induced and electrically induced seizures [9]. Further modifications on the oxazolidinone ring resulted in the synthesis of C-5 hydroxamic acid-containing oxazolidinone derivatives (Figure 2). The hydroxamic acid moiety, when present in molecules, can act as an iron-chelating functionality, which may alter the iron intake and/or metabolism in bacteria. These derivatives were devoid of potent antibacterial activity as a result of the substantial steric interaction between the hydroxamic acid N-OH oxygen atom and the binding site. Furthermore, these derivatives also showed a reduced activity as MAO-A and -B inhibitors compared with linezolid [16].

Although oxazolidinone hydroxamic acid derivatives lack antibacterial activity, some derivatives have shown excellent inhibitory activity against leukotriene (LT) biosynthesis [17]. These derivatives, exemplified by PH-211 having an *N*-cyclopropanoyl group, PH-247 having an *N*-hexanoyl group, PH-249 having an *N*-heptanoyl group, and PH-251 having an *N*-octanoyl group (Table 1), are powerful inhibitors of 5-lipoxygenase (5-LO), the elaborate enzyme in the biosynthesis of LTs. LTs are involved in multiple allergic and inflammatory diseases, including asthma, allergic rhinitis, atopic dermatitis, and rheumatoid arthritis, as well as cardiovascular diseases and certain types of cancers [18]. Therefore, targeting the 5-LO enzyme can have beneficial anti-inflammatory and anti-allergic effects via the inhibition of LT biosynthesis since it is implicated in the pathogenicity and chronicity of such diseases. Currently, zileuton is the only FDA-approved drug that is a 5-LO inhibitor, specified for the prophylactic management and long-term treatment of asthma in adults and children 12 years and above [19]. However, the use of this drug is limited due to hepatotoxicity and its unfavorable short half-life [20]. As a result, there is a need to develop new potent 5-LO inhibitors with favorable safety and pharmacokinetic profiles.

Analytical techniques utilized in pharmaceutical analysis provide essential information useful for understanding key aspects regarding bioavailability, bioequivalence, and therapeutic monitoring during patient care. Different analytical methods utilizing different instruments, mobile phase systems, and stationary phases have been reported and used to assess oxazolidinone derivatives and to identify their instability products [21,22,23,24,25,26]. Most of these methods are performed with High-Performance Liquid Chromatography (HPLC) coupled with Ultraviolet-Visible detectors (UV-Vis) or mass spectrometer detectors. Since linezolid is considered the main representative of the oxazolidinone class of compounds, several HPLC-constructed methods were developed for the analysis of linezolid in pharmaceuticals and biological samples [27,28]. Rapid and reliable stability-indicating assays using the Waters Acquity Ultra-High-Performance Liquid Chromatography with Ultraviolet detector (UHPLC-UV) system were developed and used for the analysis of novel triazolyl-oxazolidinone derivatives PH-189 and PH-192, with antibacterial and anticonvulsant activities, respectively [22,29].

We hereby report the development of simple, rapid, and accurate UHPLC bioanalytical methods to analyze (qualitatively and quantitatively) and investigate the stability of representative novel oxazolidinone hydroxamic acid derivatives PH-211, PH-247, PH-249, and PH-251, with 5-LO activity. The stability of these derivatives in human plasma in the presence of other biological compounds at 37 °C in the presence of an internal standard (IS) was determined. In addition, a simple extraction method was developed to indicate the comparative stabilities of these oxazolidinone derivatives in human plasma at 37 °C and establish the percentage recovered. Finally, stress stability experiments were performed, and decomposition products were recognized using UHPLC-MS under acidic, basic, oxidative, and elevated temperature conditions. Data obtained from this study are important for the further pharmacological evaluation of these novel compounds.

## 2. Results and Discussion

### 2.1. Chemistry

**Figure 3:**
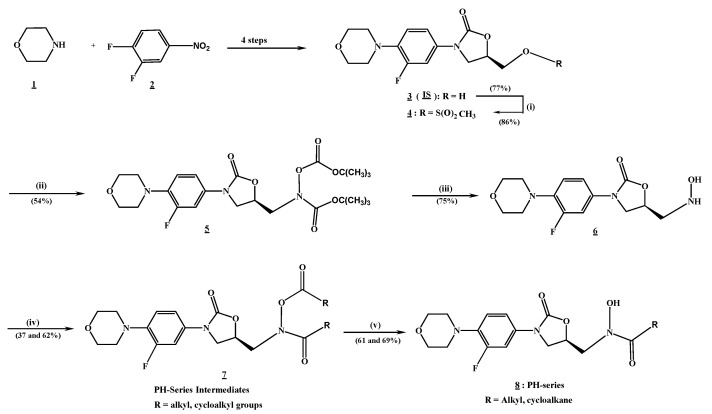
The synthetic route for the oxazolidinone hydroxamic acid derivatives. (i) DCM/TEA/CH_3_S(O)_2_Cl/0 °C to r.t., (ii) DMF/NaH/tert-Butyl-*N*-(tertbutoxycarbonyloxy) carbamate/0 °C-r.t., (iii) DCM/TFA/0 °C to r.t., (iv) DCM/TEA/RCOCl or (RCO)_2_O/0 °C to r.t., and (v) MeOH/THF/NaOH/0 °C.

The oxazolidinone hydroxamic acid derivatives (PH-211, PH-251, and the IS) were set as outlined in the chemical reaction sequence in Figure 3, along with previously reported experimental methods [16,17,30], while the other two derivatives, PH-247 and PH-249, were already available in adequate quantities in our laboratory. The compounds were obtained with a good yield and characterized using suitable spectroscopic and analytical methods, as detailed in the experimental section. As previously reported, the characteristic signals for the *N*-hydroxamic acid N–OH appeared between 9.94 and 10.18 ppm, which were exchangeable with D_2_O and as broadbands around 3181 and 3183 cm^−1^, in the nuclear magnetic resonance (^1^H NMR) and infrared (IR) spectra, respectively.

### 2.2. Method Validation for PH-211

#### 2.2.1. Linearity and Sensitivity

In the concentration range of 0.01–0.1 mg/mL, a linear correlation was established between the relative peak areas and the concentrations of PH-211. The method demonstrated excellent linearity with a correlation coefficient (r) of 0.999. The calibration curve was achieved in triplicate, with the slope and correlation coefficient showing strong consistency, which demonstrates the reliability of the standard curve over the concentration range studied. The limit of quantification (LOQ) was determined to be 0.01 mg/mL, with a relative standard deviation percentage (%RSD) of 0.22. While the limit of detection (LOD) was found to be 0.003 mg/mL, using 5 µL as the injection volume, as detailed in Table 2.

#### 2.2.2. Precision and Accuracy

The accuracy results were stated as the accuracy (%) of PH-211 in the samples. The overall results indicate that the proposed UHPLC-UV method for the analysis of PH-211 is accurate. Regarding precision, the values of %RSD for the intra-day and inter-day variation are presented in Table 3 and Table 4. The results show that in both cases, the %RSD values fell within the acceptable 2% limit, confirming the repeatability of the developed method.

#### 2.2.3. Evaluation of PH-211 Extraction and Stability in Human Plasma

To assess the efficacy of the extraction method for PH-211, the extraction recovery percentage was computed. This recovery was obtained by comparing the relative peak areas of PH-211 in plasma and the mobile phase. The extraction method was capable of extracting about 85.52% of PH-211 from human plasma, which is generally considered acceptable in regulatory and industry practice. While the International Council for Harmonisation (ICH) guidelines do not specify a precise recovery range, a value between 70% and 120% is generally acceptable. The stability assessment of PH-211 in human plasma at 37 °C for 90 min revealed no presence of degradation products. Therefore, PH-211 showed good stability in human plasma. The recovered amount of PH-211 was found to be equivalent to 0.034 mg/mL from an initial spiked amount of 0.04 mg/mL.

#### 2.2.4. Stability Study

**Figure 4:**
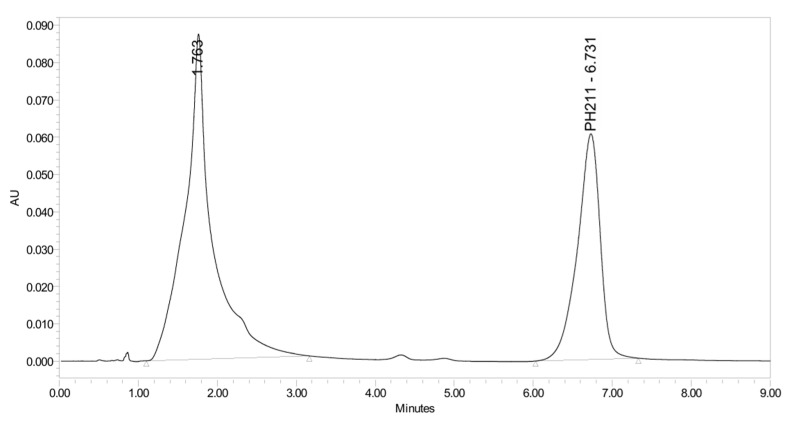
UHPLC-UV chromatogram for the acidic decomposition product of PH-211.

**Figure 5:**
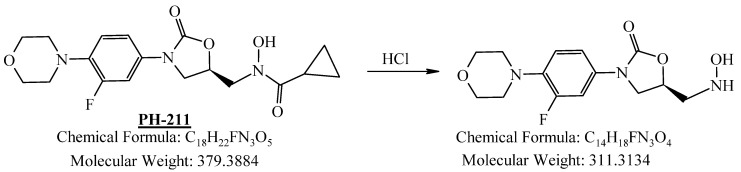
Decomposition product of PH-211 after adding 1 N HCl.

**Figure 6:**
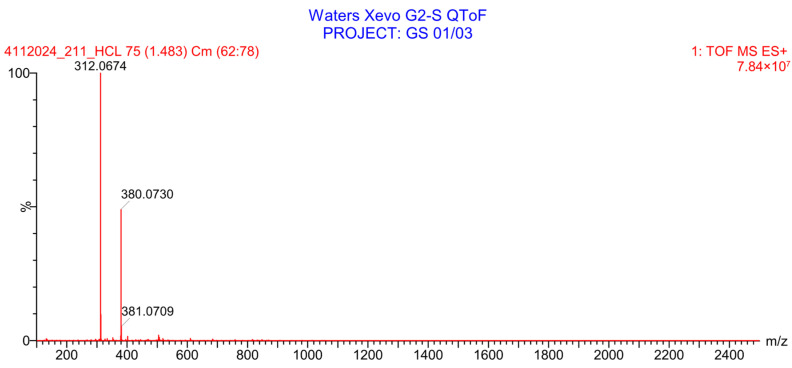
LC-QToF-MS analysis of PH-211 after subjection to acidic degradation.

**Figure 7:**
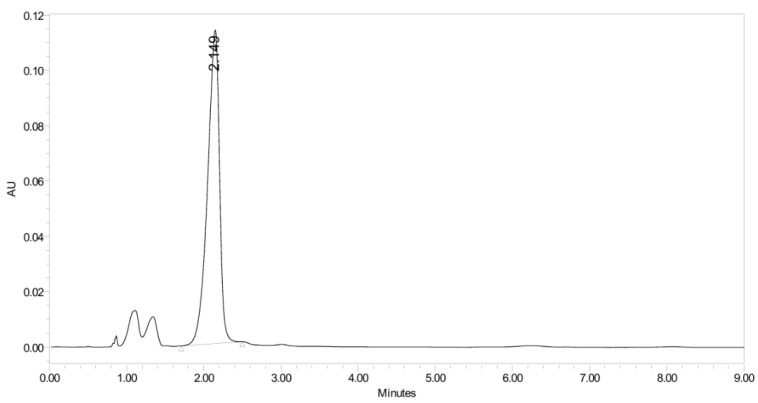
UHPLC-UV chromatogram for the basic decomposition product of PH-211.

**Figure 8:**
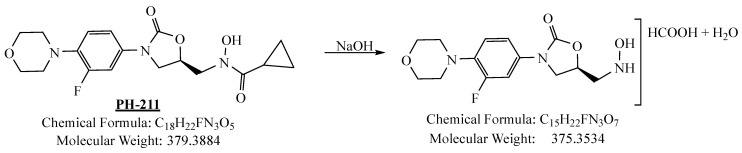
Decomposition product of PH-211 after adding 1 N NaOH.

**Figure 9:**
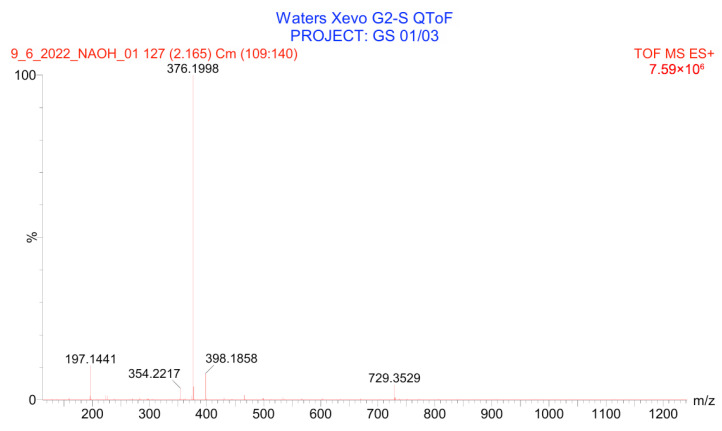
LC-QToF-MS analysis of PH-211 after exposure to basic degradation.

**Figure 10:**
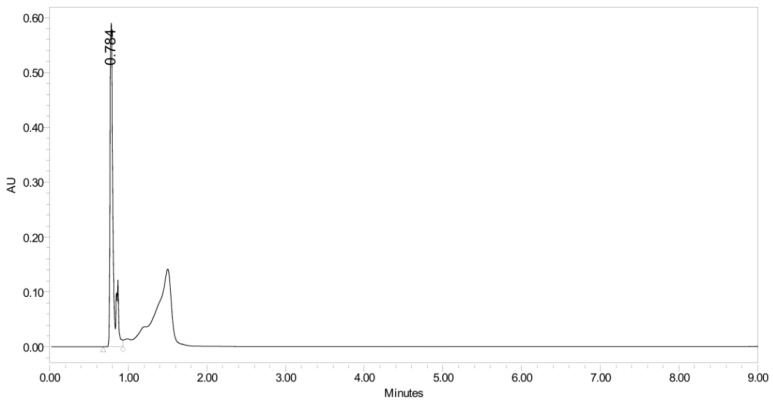
UHPLC-UV chromatogram for the oxidative degradation product of PH-211.

**Figure 11:**
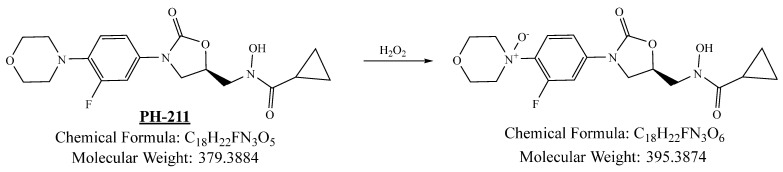
Degradation product of PH-211 after adding 1 N H_2_O_2_.

**Figure 12:**
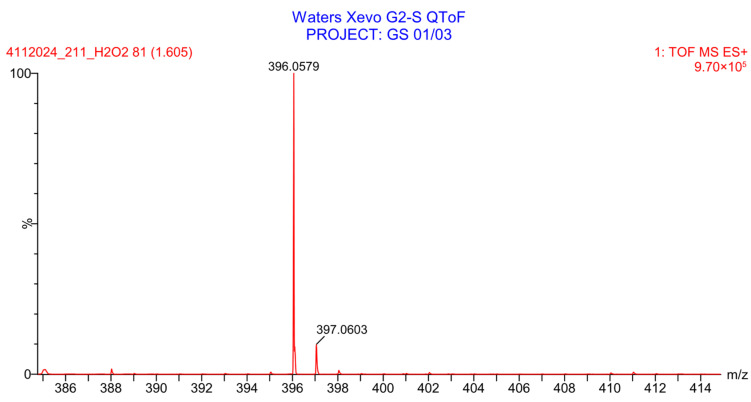
LC-QToF-MS analysis of PH-211 after subjection to oxidative degradation.

**Figure 13:**
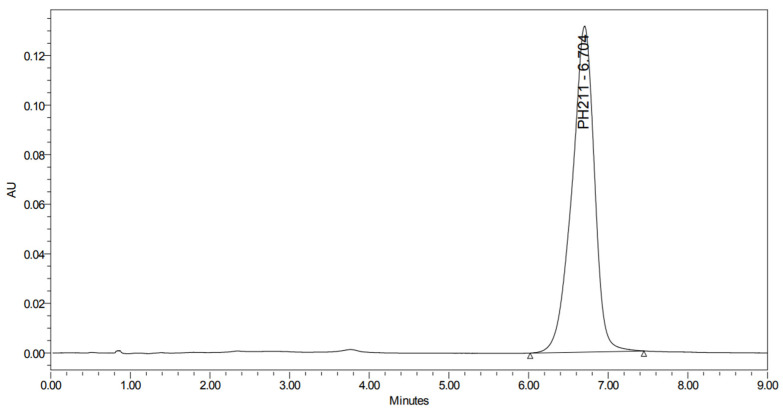
UHPLC-UV chromatogram for the thermal decomposition products of PH-211.

PH-211, having a molecular weight of 379.38 g/mol, was exposed to forced degradation under acidic, basic, oxidative, and thermal conditions. Acidic degradation using 1 N hydrochloric acid (HCl) at 90 °C for 90 min resulted in a single major degradation product detected through UHPLC-UV and was eluted in about 1.76 min, as shown in Figure 4. The acidic degradation product for PH-211 resulted from the acidic hydrolysis of the *N*-hydroxyl amide bond to produce the N-hydroxylamine, M + 1 = 312, as the degradant, as shown in Figure 5 and Figure 6. This is similar to the acidic degradation product reported for the instability of linezolid [28,31]. Similarly, basic degradation using 1 N sodium hydroxide (NaOH) at the same temperature and duration was conducted and resulted in one major degradation product detected through UHPLC-UV and was eluted in about 2.15 min, as presented in Figure 7. The product of the basic hydrolysis also corresponded to the basic hydrolysis of the *N*-hydroxyl amide bond to produce the N-hydroxylamine, which produced a mass of M + HCOOH + H_2_O + 1 = 376, as the degradant, as shown in Figure 8 and Figure 9. Moreover, PH-211 was exposed to oxidative stress conditions by adding 1 N hydrogen peroxide (H_2_O_2_) at 90 °C for 90 min. One major decomposition product was noticed through UHPLC-UV and was eluted in about 0.78 min, as shown in Figure 10. The oxidative decomposition product for PH-211 resulted in the N-oxidation of the morpholine nitrogen, M + 1 = 396, as shown in Figure 11 and Figure 12. This is similar to was reported for the degradation of linezolid in H_2_O_2_ [28,31]. The degradation products were identified and confirmed by liquid chromatography–quadrupole time-of-flight mass spectrometry (LC-QToF-MS) for all the degradation conditions. On the other hand, PH-211 was stable after heating at 90 °C for 90 min, and no degradants were found, as shown in Figure 13.

### 2.3. Method Validation for PH-247

#### 2.3.1. Linearity and Sensitivity

In the concentration range of 0.005–0.08 mg/mL, a linear relationship was established between the relative peak areas and the concentrations of PH-247. The method demonstrated excellent linearity, with a correlation coefficient (r) of 0.999. The calibration curve was achieved in triplicate, with the slope and correlation coefficient showing strong consistency, which demonstrates the reliability of the standard curve over the concentration range studied. The LOQ was determined to be 0.005 mg/mL with a %RSD of 1.35. While the LOD for PH-247 was revealed to be 0.0015 mg/mL, using 5 µL as an injection volume, as presented in Table 5.

#### 2.3.2. Precision and Accuracy

The accuracy results were expressed as the accuracy (%) of PH-247 in the samples. The aggregate results reveal that the proposed UHPLC-UV method for the analysis of PH-247 is accurate. Regarding precision, the values of %RSD for the intra-day and inter-day variation are presented in Table 6 and Table 7. The results show that in both respects, the %RSD values fell within the acceptable 2% limit, confirming the repeatability of the developed method.

#### 2.3.3. Estimation of PH-247 Extraction and Stability in Human Plasma

To assess the effectiveness of the extraction method for PH-247, the extraction recovery percentage was computed. This recovery was obtained by comparing the relative peak areas of PH-247 in plasma and the mobile phase. The extraction method was able to extract about 85.40% of PH-247 from human plasma. PH-247 displayed good stability in human plasma for 90 min at a temperature of 37 °C, and no decomposition products were detected. The recovered amount of PH-247 was found to be equivalent to 0.017 mg/mL from an initial spiked amount of 0.02 mg/mL

#### 2.3.4. Stability Study

**Figure 14:**
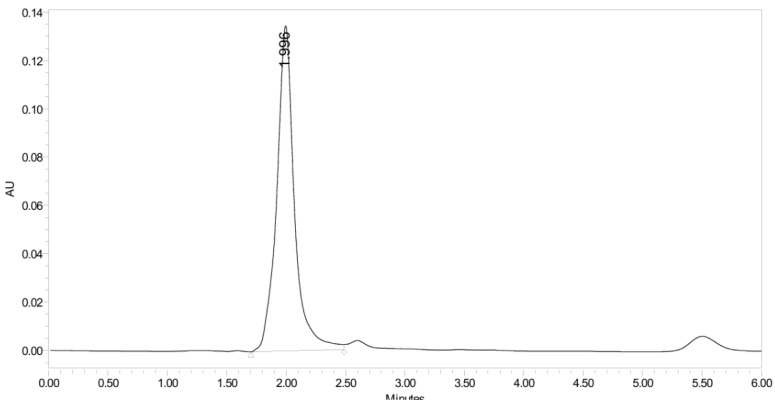
UHPLC-UV chromatogram for the acidic decomposition product of PH-247.

**Figure 15:**
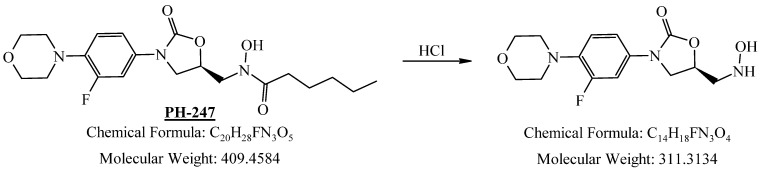
Decomposition product of PH-247 after adding 1 N HCl.

**Figure 16:**
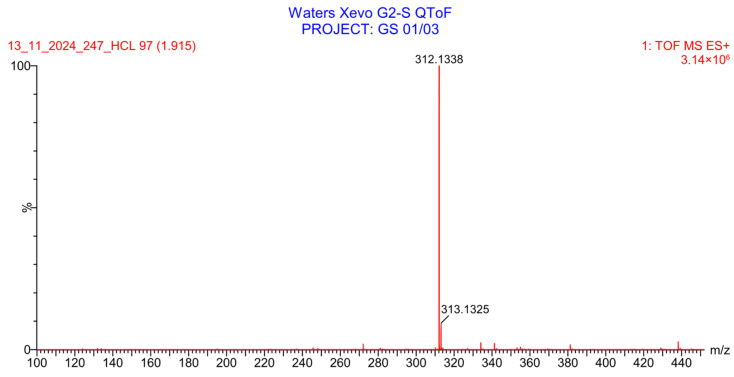
LC-QToF-MS analysis of PH-247 after subjection to acidic degradation.

**Figure 17:**
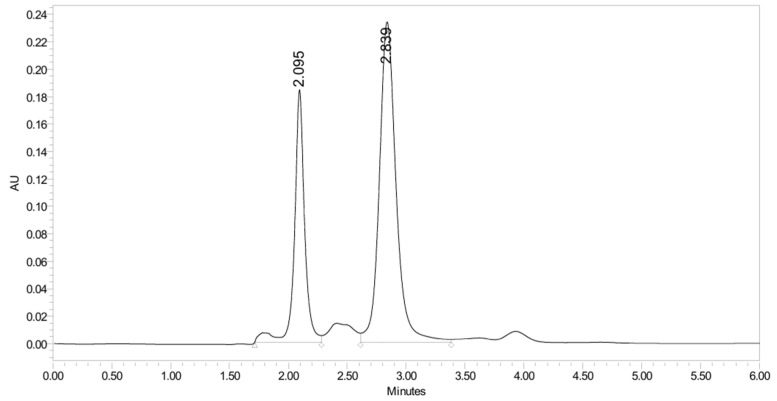
UHPLC-UV chromatogram for the basic decomposition products of PH-247.

**Figure 18:**
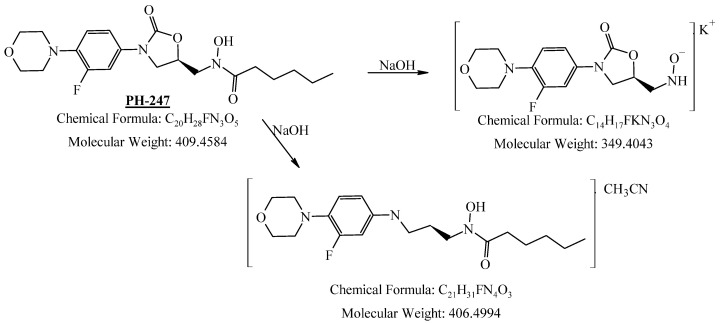
Decomposition products of PH-247 after adding 1 N NaOH.

**Figure 19:**
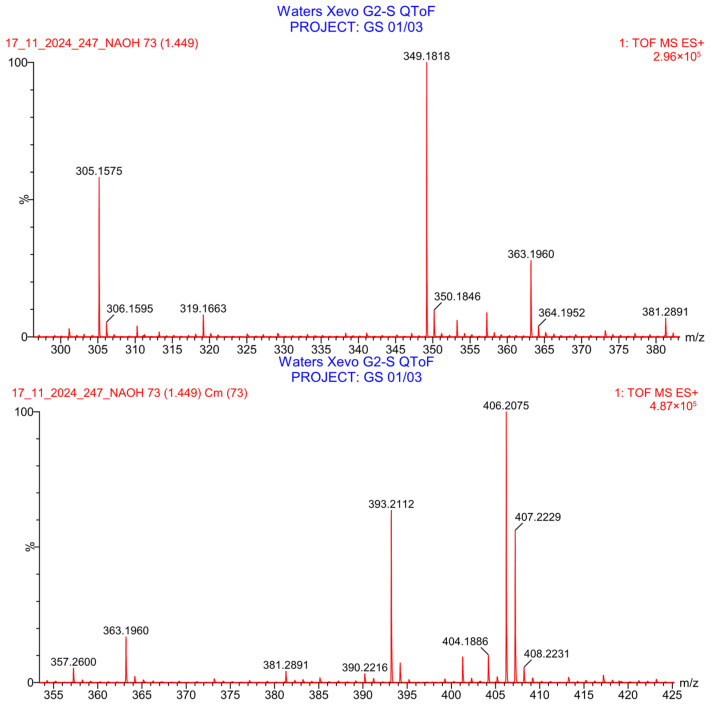
LC-QToF-MS analysis of PH-247 after subjection to basic degradation.

**Figure 20:**
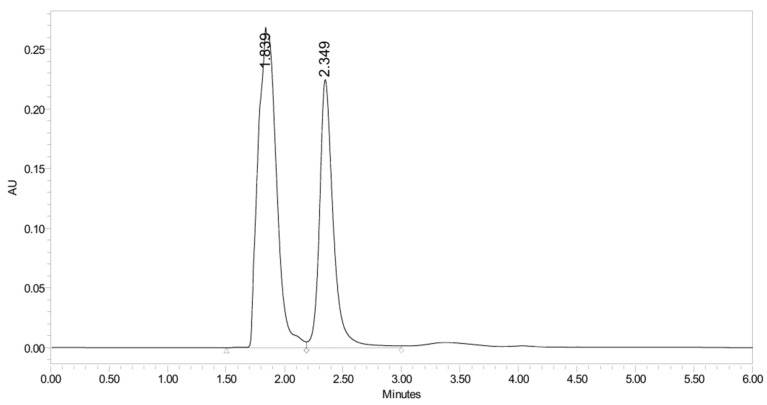
UHPLC-UV chromatogram for the oxidative decomposition products of PH-247.

**Figure 21:**
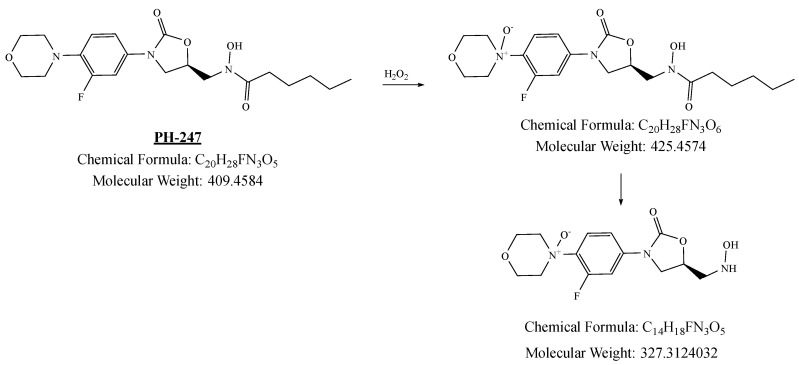
Decomposition products of PH-247 after adding 1 N H_2_O_2_.

**Figure 22:**
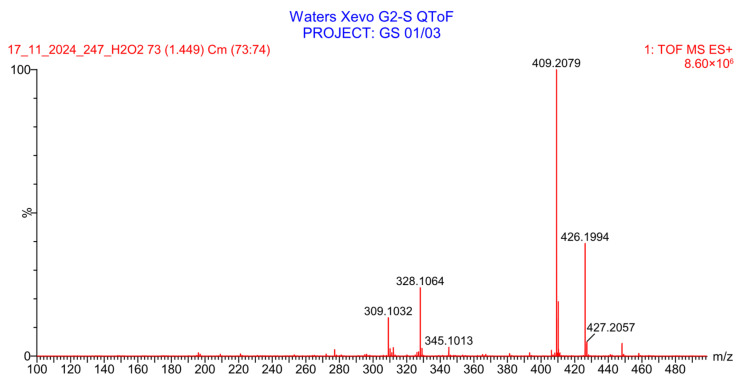
LC-QToF-MS analysis of PH-247 after subjection to oxidative degradation.

**Figure 23:**
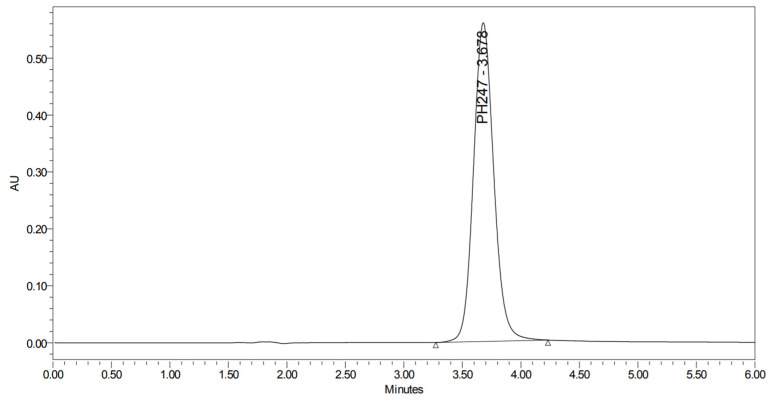
UHPLC-UV chromatogram for the thermal decomposition products of PH-247.

PH-247 with a molecular weight of 409.45 g/mol was exposed to forced decomposition under acidic, basic, oxidative, and thermal conditions. Acidic degradation using 1 N HCl at 90 °C for 90 min resulted in one main degradation product detected through UHPLC-UV and was eluted by around 1.99 min, as shown in Figure 14. The acidic degradation product for PH-247 resulted from the acidic hydrolysis of the *N*-hydroxyl amide bond to produce the N-hydroxylamine, M + 1 = 312, as the degradant, as shown in Figure 15 and Figure 16. This is similar to the acidic degradation product reported for the instability of linezolid [28,31]. In addition, a basic decomposition by 1 N NaOH at 90 °C for 90 min was conducted. Two major degradation products were observed through UHPLC-UV and were eluted in about 2.09 and 2.84 min, as shown in Figure 17. The two products of the basic hydrolysis corresponded to the basic hydrolysis of the *N*-hydroxyl amide bond to produce the N-hydroxylamine as a potassium salt [M + 1 = 350] and the oxazolidinone ring cleave product, which resulted in a mass of M + CH3CN + 1 = 407, as shown in Figure 18 and Figure 19. Similar ring-opened degradants have been reported for the instability of linezolid [28,31], sutezolid [32], and tedizolid [33]. Moreover, PH-247 was exposed to oxidative stress conditions by adding 1 mL of 1 N H_2_O_2_ and heating at 90 °C for 90 min. Two main degradation products were detected through UHPLC-UV and were eluted around 1.84 and 2.35 min, as shown in Figure 20. Oxidative degradation products for PH-247 included the N-oxidation of the morpholine nitrogen of PH-247, M + 1 = 426, and the N-oxidated N-hydroxylamine degradant M + 1 = 328, as shown in Figure 21 and Figure 22. This is similar to what was reported for the oxidative degradation of linezolid in H_2_O_2_ [28,31]. The degradation products were identified and confirmed by LC-QToF-MS for all the degradation conditions. On the other hand, PH-247 was stable after heating at 90 °C for 90 min, and no degradants were found, as shown in Figure 23.

### 2.4. Method Validation for PH-249

#### 2.4.1. Linearity and Sensitivity

In the concentration range of 0.01–0.1 mg/mL, a linear correlation was established between the relative peak areas and the concentrations of PH-249. The method demonstrated excellent linearity, with a correlation coefficient (r) of 0.999. The calibration curve was achieved in triplicate, with the slope and correlation coefficient showing strong consistency, which demonstrates the reliability of the standard curve over the concentration range studied. The LOQ was revealed to be 0.01 mg/mL, with a %RSD of 0.13. While the LOD for PH-249 was revealed to be 0.003 mg/mL, using 5 µL as the injection volume, as presented in Table 8.

#### 2.4.2. Precision and Accuracy

The accuracy results were stated as the accuracy (%) of PH-249 in the samples. The aggregate results indicate that the proposed UHPLC-UV method for the analysis of PH-249 is accurate. Regarding precision, the values of %RSD for the intra-day and inter-day variation are presented in Table 9 and Table 10. The results show that in both respects, the %RSD values fell within the acceptable 2% limit, confirming the repeatability of the developed method.

#### 2.4.3. Evaluation of PH-249 Extraction and Stability in Human Plasma

To assess the effectiveness of the extraction method for PH-249, the extraction recovery percentage was computed. This recovery was obtained by comparing the relative peak areas of PH-249 in plasma and the mobile phase. The extraction method was capable of extracting about 90.20% of PH-249 from human plasma. PH-249 showed good stability in human plasma for 90 min at a temperature of 37 °C, and no degradants were detected. The recovered amount of PH-249 was found to be equivalent to 0.036 mg/mL from an initial spiked amount of 0.04 mg/mL.

#### 2.4.4. Stability Study

**Figure 24:**
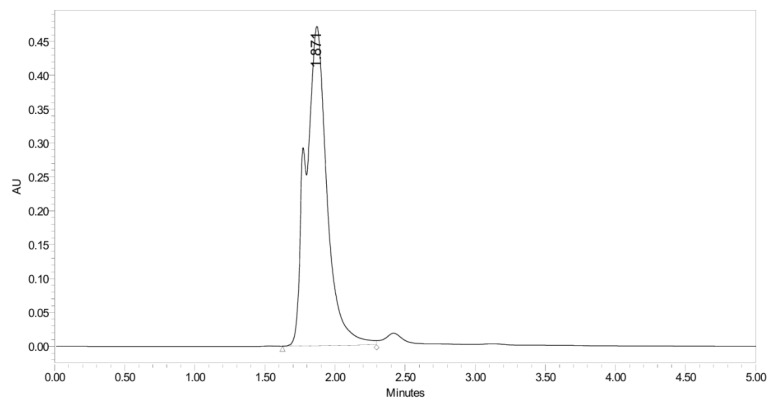
UHPLC-UV chromatogram for the acidic degradation product of PH-249.

**Figure 25:**
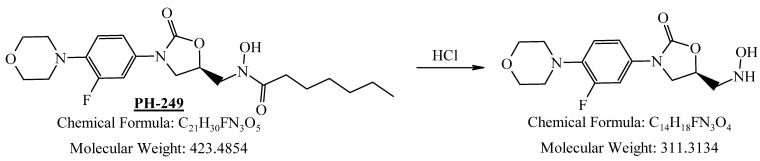
Degradation product of PH-249 after adding 1 N HCl.

**Figure 26:**
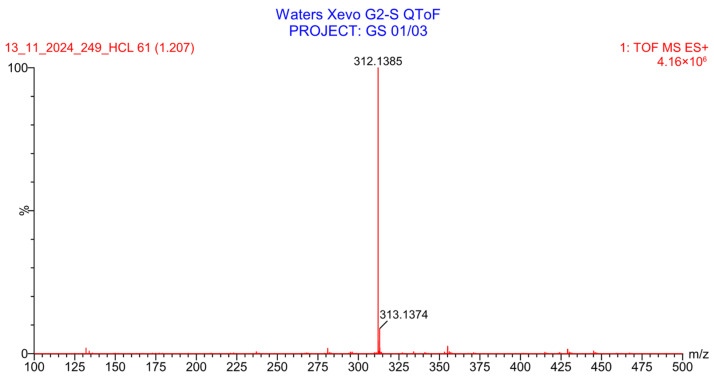
LC-QToF-MS analysis of PH-249 after subjection to acidic decomposition.

**Figure 27:**
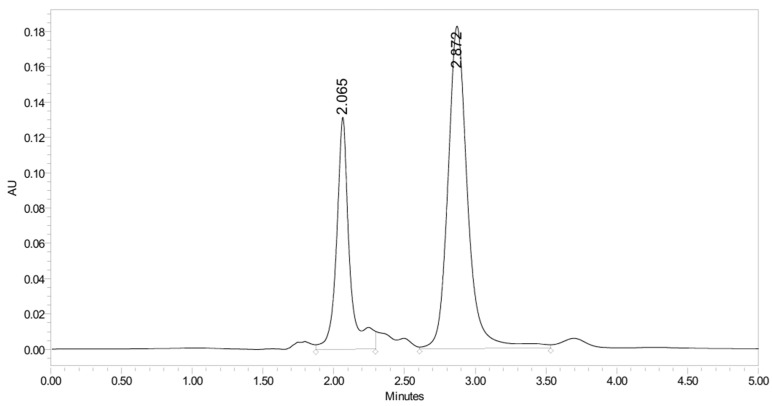
UHPLC-UV chromatogram for the basic decomposition products of PH-249.

**Figure 28:**
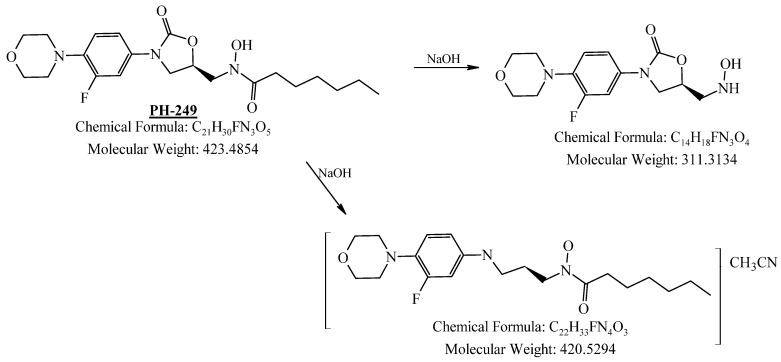
Decomposition products of PH-249 after adding 1 N NaOH.

**Figure 29:**
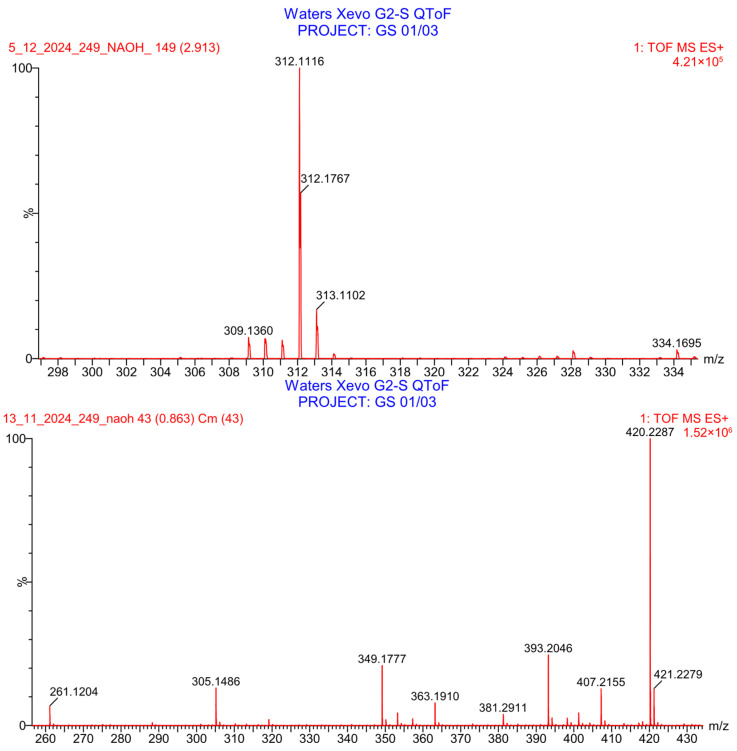
LC-QToF-MS analysis of PH-249 after exposure to basic degradation.

**Figure 30:**
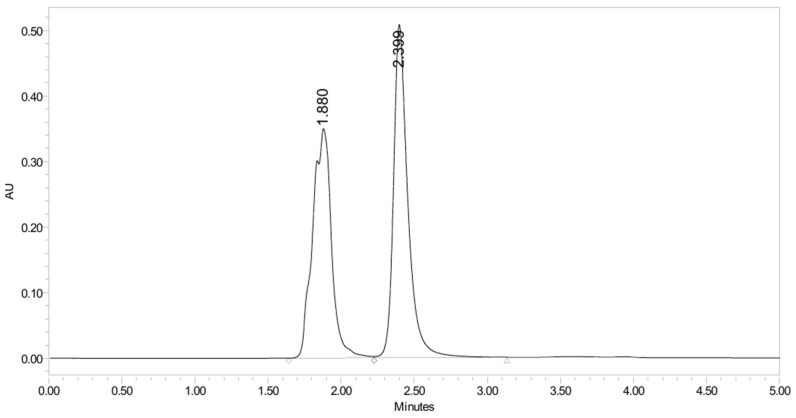
UHPLC-UV chromatogram for the oxidative decomposition products of PH-249.

**Figure 31:**
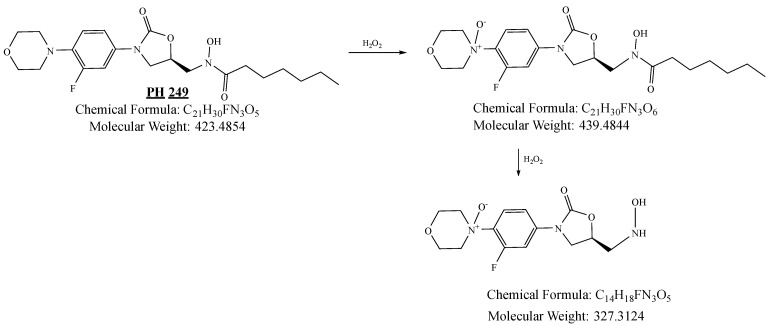
Decomposition products of PH-249 after adding 1 N H_2_O_2_.

**Figure 32:**
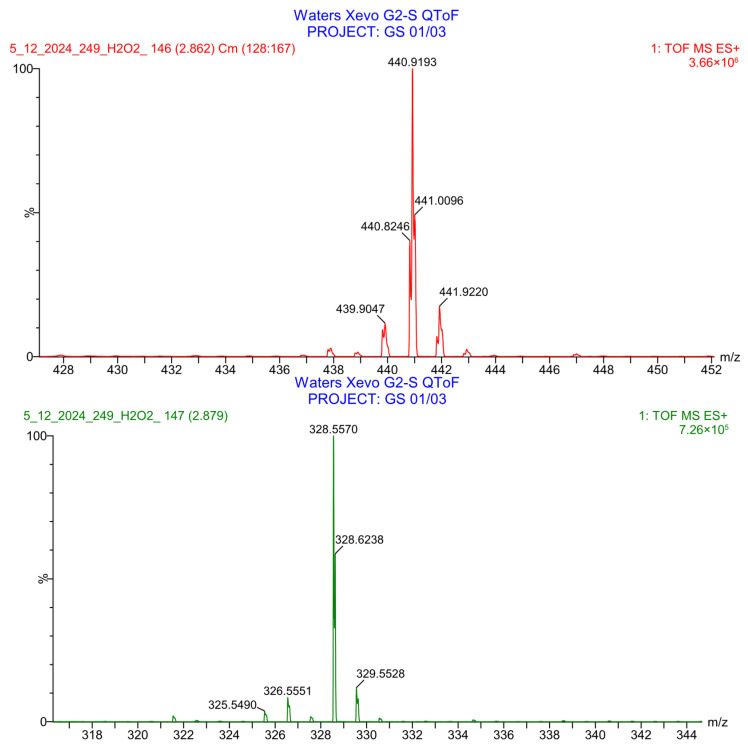
LC-QToF-MS analysis of PH-249 after exposure to oxidative decomposition.

**Figure 33:**
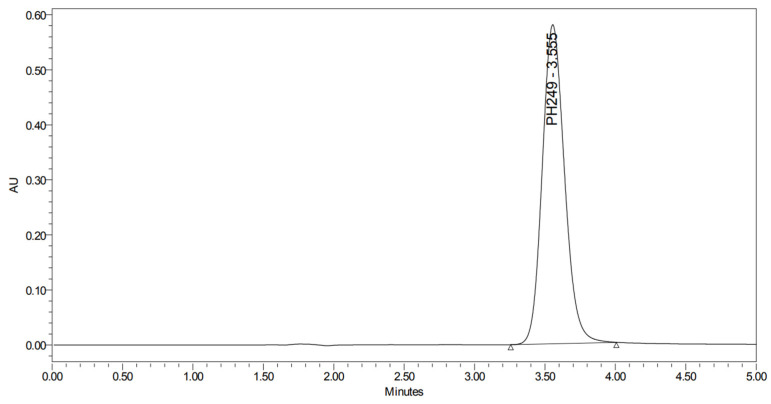
UHPLC-UV chromatogram for the thermal decomposition products of PH-249.

PH-249 with a molecular weight of 423.49 g/mol was exposed to acidic decomposition by 1 N HCl and heating at 90 °C for 90 min. One main decomposition product was noticed through UHPLC-UV and was eluted in about 1.87 min, as shown in Figure 24. The acidic decomposition product for PH-249 resulted from the acidic hydrolysis of the *N*-hydroxyl amide bond to produce the N-hydroxylamine, M + 1 = 312, as the degradant, as shown in Figure 25 and Figure 26. This is similar to the acidic degradation product reported for the instability of linezolid [28,31]. In addition, the basic decomposition by 1 N NaOH and heating at 90 °C for 90 min was conducted. Two major decomposition products were detected through UHPLC-UV and were eluted in about 2.07 and 2.87 min, as shown in Figure 27. The two products of the basic hydrolysis for PH-249 are due to the basic hydrolysis of the *N*-hydroxyl amide bond to produce the N-hydroxylamine [M + 1 = 312] and the oxazolidinone ring cleave product, which resulted in a mass of M + CH3CN + 1 = 421, as shown in Figure 28 and Figure 29. Similar ring-opened degradants have been reported for the instability of linezolid [28,31], sutezolid [32], and tedizolid [33] and observed for PH-247. Moreover, PH-249 was exposed to oxidative stress conditions by adding 1 mL of 1 N H_2_O_2_ and heating at 90 °C for 90 min. Two major degradation products were detected through UHPLC-UV and were eluted around 1.88 and 2.40 min, as shown in Figure 30. The oxidative degradants obtained for PH-249 are similar to those for PH-247 and included the N-oxidation of the morpholine nitrogen of PH-249, M + 1 = 440, and the N-oxidated N-hydroxylamine degradant M + 1 = 328 as shown in Figure 31 and Figure 32. This is similar to what was reported for the oxidative degradation of linezolid in H_2_O_2_ [28,31]. The degradation products were identified and confirmed by LC-QToF-MS for all the degradation conditions. On the other hand, PH-249 was stable after heating at 90 °C for 90 min, and no degradants were found, as shown in Figure 33.

### 2.5. Method Validation for PH-251

#### 2.5.1. Linearity and Sensitivity

In the concentration range of 0.01–0.1 mg/mL, a linear relationship was obtained between the relative peak areas and the concentrations of PH-251. The method demonstrated excellent linearity, with a correlation coefficient (r) of 0.999. The calibration curve was achieved in triplicate, with the slope and correlation coefficient showing strong consistency, which demonstrates the reliability of the standard curve over the concentration range calculated. The LOQ was revealed to be 0.01 mg/mL, with a %RSD of 0.17. While the LOD for PH-211 was revealed to be 0.003 mg/mL, using 5 µL as the injection volume, as presented in Table 11.

#### 2.5.2. Precision and Accuracy

The accuracy results were stated as the accuracy (%) of PH-251 in the samples. The aggregate results indicate that the proposed UHPLC-UV method for the analysis of PH-251 is accurate. Regarding precision, the values of %RSD for the intra-day and inter-day variation are presented in Table 12 and Table 13. The results show that in both respects, the %RSD values fell within the acceptable 2% limit, confirming the repeatability of the developed method.

#### 2.5.3. Assessment of PH-251 Extraction and Stability in Human Plasma

To assess the effectiveness of the extraction method for PH-251, the extraction recovery percentage was computed. This recovery was obtained by comparing the relative peak areas of PH-251 in plasma and the mobile phase. The extraction method was able to extract about 82.20% of PH-251 from human plasma. PH-251 displayed good stability in human plasma for 90 min at a temperature of 37 °C, and no decomposition products were noticed. The recovered quantity of PH-251 was found to be equivalent to 0.033 mg/mL from an initial spiked amount of 0.04 mg/mL.

#### 2.5.4. Stability Study

**Figure 34:**
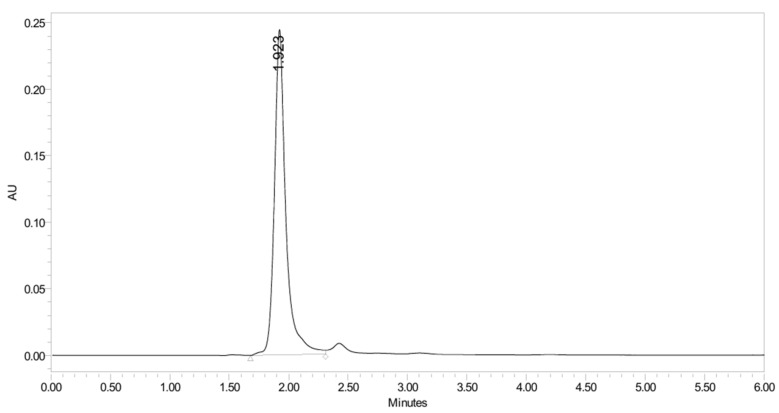
UHPLC-UV chromatogram for the acidic degradation product of PH-251.

**Figure 35:**
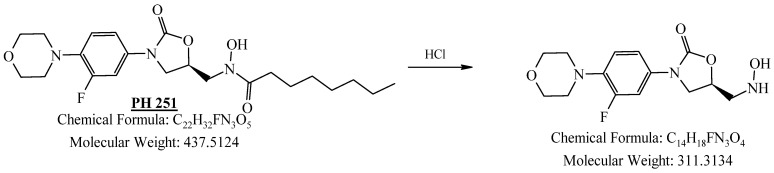
Degradation product of PH-251 after adding 1 N HCl.

**Figure 36:**
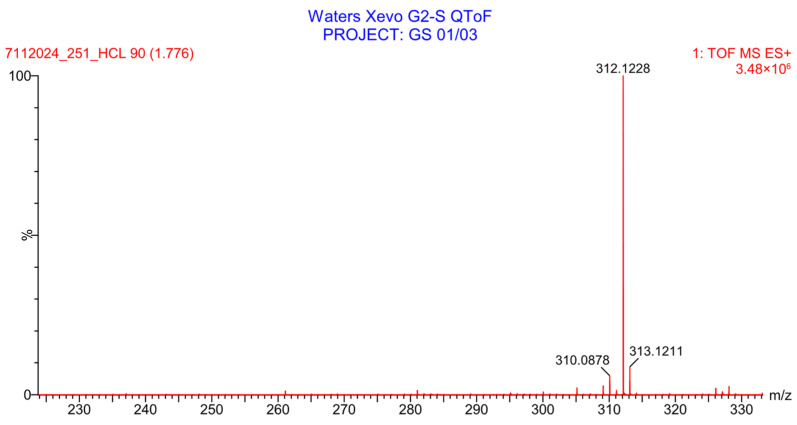
LC-QToF-MS analysis of PH-251 after subjection to acidic decomposition.

**Figure 37:**
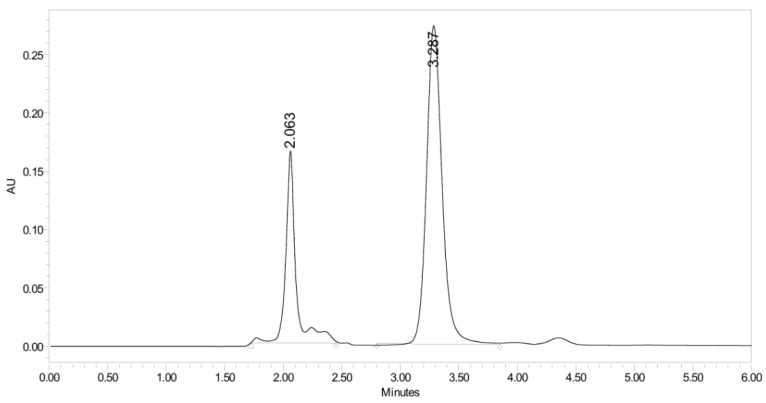
UHPLC-UV chromatogram for the basic decomposition products of PH-251.

**Figure 38:**
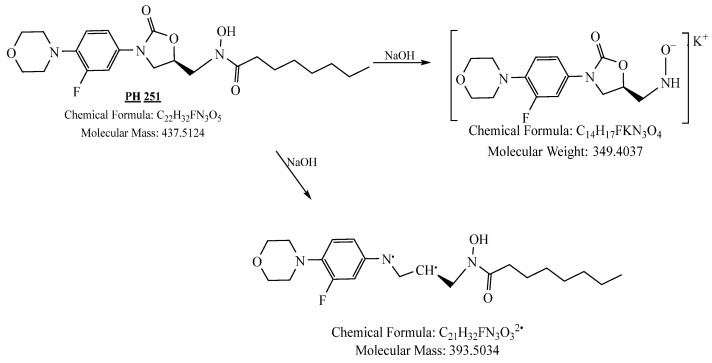
Decomposition products of PH-251 after adding 1 N NaOH.

**Figure 39:**
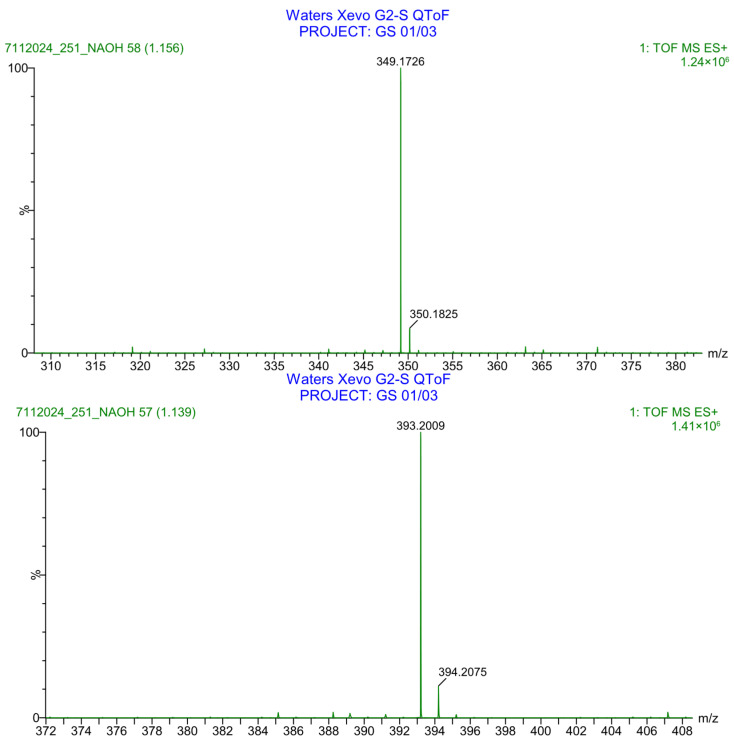
LC-QToF-MS analysis of PH-251 after subjection to basic decomposition.

**Figure 40:**
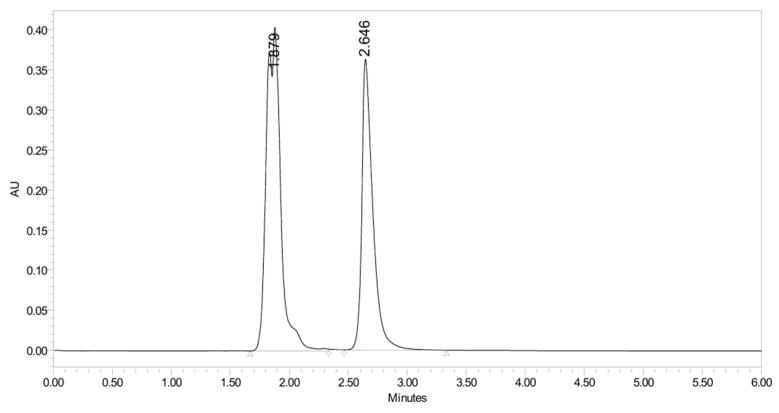
UHPLC-UV chromatogram for the oxidative decomposition products of PH-251.

**Figure 41:**
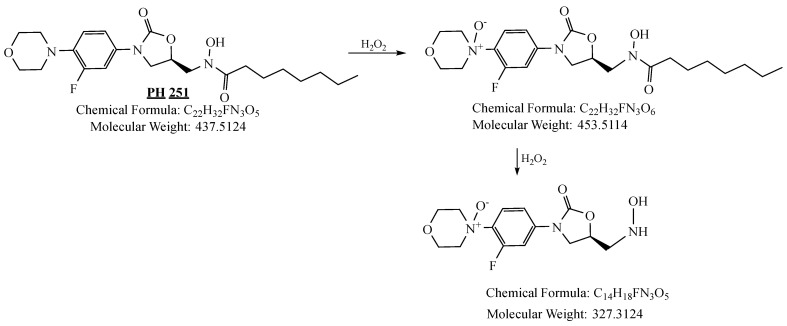
Decomposition products of PH-251 after adding 1 N H_2_O_2_.

**Figure 42:**
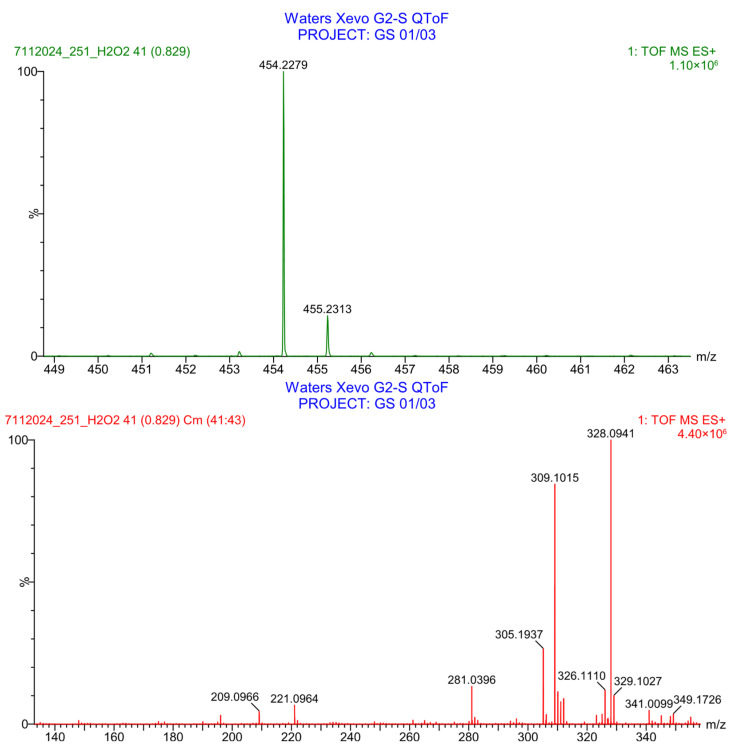
LC-QToF-MS analysis of PH-251 after subjection to oxidative decomposition.

**Figure 43:**
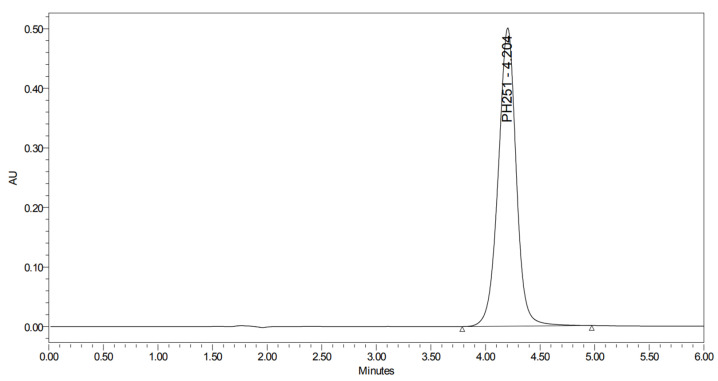
UHPLC-UV chromatogram for the thermal decomposition products of PH-251.

PH-251 with a molecular weight of 437.51 g/mol was exposed to acidic decomposition by 1 N HCl and heating at 90 °C for 90 min. One major decomposition product was detected through UHPLC-UV and was eluted in about 1.92 min, as presented in Figure 34. The acidic decomposition product for PH-251 resulted from the acidic hydrolysis of the *N*-hydroxyl amide bond to produce the *N*-hydroxylamine, M + 1 = 312, as the degradant, as shown in Figure 35 and Figure 36. This is similar to the acidic degradation product reported for the instability of linezolid [28,31]. In addition, the basic decomposition by 1 N NaOH and heating at 90 °C for 90 min was conducted. Two major decomposition products were noticed through UHPLC-UV and were eluted in about 2.06 and 3.29 min, as shown in Figure 37. The basic hydrolysis of PH-251 produced the basic hydrolysis of the *N*-hydroxyl amide bond to produce the N-hydroxylamine as a potassium salt [M + 1 = 350] and the oxazolidinone ring cleave product, which resulted in a mass of M + 1 = 394, as shown in Figure 38 and Figure 39. Similar ring-opened degradants have been reported for the instability of linezolid [28,31], sutezolid [32], and tedizolid [33] and also obtained for PH-247 and PH-249 in this study. Moreover, PH-251 was exposed to oxidative stress conditions by adding 1 mL of 1 N H_2_O_2_ and heating at 90 °C for 90 min. Two major decomposition products were noticed through UHPLC-UV and were eluted in about 1.88 and 2.65 min, as shown in Figure 40. The oxidative degradants obtained for PH-251 are similar to those for PH-247 and PH-249 and included the N-oxidation of the morpholine nitrogen of PH-251, M + 1 = 454, and the N-oxidated N-hydroxylamine degradant M + 1 = 328, as shown in Figure 41 and Figure 42. This is similar to what was reported for the oxidative degradation of linezolid in H_2_O_2_ [28,31]. The degradation products were identified and confirmed by LC-QToF-MS for all the degradation conditions. On the other hand, PH-251 was stable after heating at 90 °C for 90 min, and no degradants were found, as shown in Figure 43.

## 3. Experimental Section

### 3.1. Materials and Methods

#### 3.1.1. Chemistry

The oxazolidinone hydroxamic acid derivatives (PH-211, PH-251, and the IS) were prepared according to previously reported experimental methods, as outlined in the chemical reaction sequence shown in Figure 3 [16,17,30]. All solvents and reagents used in the synthesis of the oxazolidinone hydroxamic acid derivatives were purchased from Merck Sigma-Aldrich, Germany, such as acetone, acetonitrile (ACN), ethyl acetate, methanol, diethyl ether, hexane, dimethyl formamide (DMF), trifluoroacetic acid (TFA), dichloromethane (DCM), and anhydrous tetrahydrofuran (THF). 3,4-difluoronitrobenzene and morpholine were used as the starting materials for the synthesis. Several others were also used for the synthesis, such as n-butyllithium, triethylamine (TEA), and sodium hydride. Thin-layer chromatography (TLC) was performed using 0.25 mm pre-coated silica gel plates. Some of the compounds were purified by silica gel column chromatography using silica gel (Kieselgel 60, 70–230 mesh; Sigma-Aldrich, Darmstadt, Germany). A Stuart Scientific melting point apparatus (SMP1, Stuart, Stone, UK) was used for determining the melting point, and the results reported were uncorrected. Structural characterization and elucidation were conducted using appropriate spectroscopic methods. Infrared (IR) spectra were recorded on JASCO FT-IR-6300 (JASCO, Tokyo, Japan) Spectrometer. For ^1^H nuclear magnetic resonance (NMR) spectra, dimethyl sulfoxide (DMSO) was used, and signals were recorded on Bruker Avance AV Neo 400 and 600 MHz NMR spectrometers; ^13^C NMR spectra of representative final compounds was recorded on Bruker Avance AV Neo 400. Mass spectra data were recorded using Waters QToF-Mass Spectrometer (Waters Corporation, Milford, MA, USA). Elemental analyses were conducted using an Elementar Vario Micro Cube CHN Analyser apparatus (Elementar, Langenselbold, Germany), and the experimental values were found to be within ±0.4% of the theoretical values.

#### 3.1.2. Chemicals

PH-211, PH-251, and the IS were synthesized and characterized as previously mentioned. PH-247 and PH-249 were already available in the laboratory in adequate quantities, and their purity was also confirmed by TLC and spectrometric methods. Drug-free human plasma was obtained from Kuwait Blood Bank, Al Jabriyah, Kuwait. All solvents used in the method were of analytical grade, namely, acetonitrile (ACN) and formic acid, and other chemicals were obtained from Sigma-Aldrich, Germany. The water used throughout the study was pure and produced in-house using a MilliQ filter obtained from Millipore, Watford, UK. Syringe membrane filters (20 µm) were purchased from Kinesis Scientific Expert, Cambridgeshire, UK. For solvent filtration, 0.45 µm nylon solvent filters were utilized.

#### 3.1.3. Preparation of (R)-N-((3-(3-Fluoro-4-morpholinylphenyl)-2-oxooxazolidin-5-yl)methyl)-N-hydroxycyclopropanecarboxamide (PH-211)

PH-211 was prepared according to previously reported experimental methods, as outlined in the chemical reaction sequence shown in Figure 3 [16,17,30], and recrystallized from ethyl acetate and obtained as a white solid, 0.51 g, 61% yield. IR (KBr, cm^−1^): *ν* 3181 (OH stretch), 2857 (C-H stretch), 1720 (C=O stretch), 1617 (C=C stretch), 1474 (C-H bend), 1329 (OH bend), 1229 (C-O stretch, C-N stretch), 1112 (C-F stretch), 942 (C-H bend). ^1^H NMR (DMSO-d_6_, 400 MHz): δ 10.18 (s, 1H, exchangeable with D_2_O, NOH), δ 7.49 (dd, 1H, *J* = 2.5 Hz, *J* = 15.0 Hz, phenyl H), δ 7.17–7.19 (m, 1H, phenyl H), δ 7.06 (t, 1H, *J* = 9.4 Hz, phenyl H), δ 4.87–4.91 (m, 1H, oxazolidinone C^5^H), δ 4.04–4.16 (m, 2H, oxazolidinone C^4^H), δ 3.67–3.77 (m, 6H, morpholine CH_2_OCH_2_ and methylene CH_2_), δ 2.96 (t, 4H, *J* = 4.6 Hz, morpholine CH_2_NCH_2_), δ 2.26 (br. s, 1H, cyclopropyl C^1^H), δ 0.72–0.80 (m, 4H, cyclopropyl (CH_2_)_2_). ^13^C NMR (DMSO-d_6_, 400 MHz): δ 174.22, 155.76, 153.94, 153.33, 135.58, 135.50, 133.45, 133.35, 119.23, 119.19, 114.18, 114.15, 106.84, 106.58, 69.81, 66.12, 50.69, 50.66, 47.53, 9.61, 7.55, 7.52. MS (*m*/*z*): 380.25 (M^+^ + H). Anal. Calcd. for C_18_H_22_FN_3_O_5_: C: 56.99; H: 5.85; N: 11.08; found C: 57.09; H, 5.73; N, 11.22.

#### 3.1.4. Preparation of (R)-N-((3-(3-Fluoro-4-morpholinophenyl)-2-oxooxazolidin-5-yl)methyl)-N-hydroxyoctanamide (PH-251)

PH-251 was prepared according to previously reported experimental methods, as outlined in the chemical reaction sequence shown in Figure 3 [16,17,30], and recrystallized from ethyl acetate–hexane 2:1 to produce a white solid, 0.47 g, 68.72% yield. IR (KBr, cm^−1^): *ν* 3183 (OH stretch), 2956, 2921, 2853 (C-H stretch), 1718 (C=O stretch), 1624 (C=O stretch), 1518 (C=C stretch), 1423 (C-H bend), 1380 (CH_3_ bend), 1330 (OH bend), 1231 (C-O stretch, C-N stretch), 1112 (C-F stretch), 942 (C-H bend), 747 (CH long chain methyl rock). ^1^H NMR (DMSO-d_6_, 400 MHz): δ 9.94 (br. s, 1H, exchangeable with D_2_O, NOH), δ 7.48 (dd, 1H, *J* = 2.5 Hz, *J* = 15.0 Hz, phenyl H), δ 7.17 (dd, 1H, *J* = 2.0 Hz, *J* = 8.8 Hz, phenyl H), δ 7.06 (t, 1H, *J* = 9.4 Hz, phenyl H), δ 4.86–4.88 (m, 1H, oxazolidinone C^5^H), δ 4.12 (t, 1H, *J* = 8.9 Hz, oxazolidinone C^4^H), δ 4.05 (dd, 1H, *J* = 6.7 Hz, *J* = 14.7 Hz, oxazolidinone C^4^H), δ 3.72–3.76 (m, 5H, morpholine CH_2_OCH_2_ and methylene H), δ 3.67 (dd, 1H, *J* = 4.4 Hz, *J* = 14.7 Hz, methylene H), δ 2.96 (t, 4H, *J* = 4.6 Hz, morpholine CH_2_NCH_2_), δ 2.35–2.38 (m, 2H, methylene CH_2_C=O), δ 1.45–1.49 (m, 2H, methylene CH_2_), δ 1.23–1.27 (m, 8H, methylene CH_2_), δ 0.85 (t, 3H, *J* = 6.9 Hz, CH_3_). ^13^C NMR (DMSO-d_6_, 400 MHz): δ 173.95, 155.76, 153.93, 153.34, 135.54, 135.46, 133.47, 133.36, 119.21, 119.17, 114.10, 114.07, 106.77, 106.51, 69.92, 66.12, 50.69, 50.66, 50.27, 47.44, 31.55, 31.11, 28.68, 28.46, 24.09, 22.01, 13.89. MS (*m*/*z*): 438.34 (M^+^ + H). Anal. Calcd. for C_22_H_32_FN_3_O_5_: C: 60.40; H: 7.37; N: 9.60; found C: 60.49; H, 7.20; N, 9.96.

#### 3.1.5. Solutions

Stock standard solutions of the compounds (PH-211, PH-247, PH-249, PH-251, and the IS (HM.05)) were prepared separately by dissolving 10 mg of the compounds in 10 mL of CAN, producing stock concentrations of 1 mg/mL. Working solutions were set up by diluting 1 mL of the stock solution with 9 mL of ACN to produce a concentration of 0.1 mg/mL. Stock solutions were kept in a refrigerator at (4 °C) and remained stable for at least 6 weeks.

#### 3.1.6. Human Plasma Extraction Technique

For each compound, aliquots of 300 µL of human plasma were blended with 500 µL of the compound (0.2 mg/mL), followed by the addition of 200 µL of HM.05 as an internal standard in an Eppendorf tube. The mixture was vortexed thoroughly and then centrifuged at 11,290 revolutions per minute for 10 min. The supernatant was filtered using syringe membrane filters (20 µm) then placed into HPLC vials, producing a final solution prepared for analysis.

### 3.2. Instrumentation

#### 3.2.1. Ultra-High-Performance Liquid Chromatography–Ultraviolet (UHPLC-UV)

The analysis and method validation were conducted using Waters Acquity UHPLC system equipped with a quaternary Solvent Manager (H-Class), Sample Manager, and ACE C4 (150 × 3.0 mm, 3 µm) analytical columns. Isocratic elution and a UV detector were employed. Data obtained was processed and reported using Empower^®^ software version 3.8.1.1.

#### 3.2.2. Chromatographic Conditions

The chromatographic conditions were optimized using a mobile phase consisting of filtered and degassed 0.1% formic acid in ACN (Solvent A) and 0.1% formic acid in water (Solvent B) in the following proportions (PH-211 15:85 *v*/*v*, flow rate 1 mL/min), (PH-247 45:55 *v*/*v*, flow rate 0.4 mL/min), (PH-249 50:50 *v*/*v*, flow rate 0.4 mL/min), and (PH-251 50:50 *v*/*v*, flow rate 0.4 mL/min). The temperature of the column was set to 50 °C throughout the study, and 5 µL of the sample was processed and analyzed at a wavelength of 254 nm.

#### 3.2.3. Ultra-High-Performance Liquid Chromatography–Mass Spectrometry (UHPLC-MS)

Waters Xevo G2-S linked with Waters Acquity UHPLC system with binary Solvent Manager (I-Class) via the quadrupole time-of-flight (QToF) interface was utilized. The operating parameters in the positive ion mode were used. The capillary and sampling cone voltages were set at 3.5 V and 115 V, respectively. The source temperature was maintained at 120 °C, with the desolvation temperature set at 350 °C.

### 3.3. Method Validation

The methods were validated following the guidelines established by the International Council for Harmonisation (ICH) [34].

#### 3.3.1. Calibration Curve

Accurately measured amounts of the compounds and the internal standard were transferred from freshly prepared stock solution (0.1 mg/mL) into the HPLC vials and filled to volume with ACN. Five concentration levels (0.01–0.1 mg/mL) were used to construct the calibration curve for PH-211, PH-251, and PH-249, while 0.005–0.08 mg/mL was used for PH-247. The samples were processed separately into the column using a flow rate of 1 mL/min for PH-211 and 0.4 mL/min for PH-247, PH-249, and PH-251. The relative peak area of each compound was plotted versus its concentration, the linearity of the curves was plotted, and regression equations were calculated.

#### 3.3.2. Accuracy and Precision

The accuracy of the results was indicated by calculating the accuracy (%) of three replicates of the lowest, middle, and highest points of each compound using (0.01, 0.04, and 0.1 mg/mL) for PH-211, PH-249, and PH-251, while (0.005, 0.02, and 0.08 mg/mL) for PH-247 covering the linearity. For assessing the precision of the UHPLC-UV method, the lowest, middle, and highest points (0.01, 0.04, and 0.1 mg/mL) were taken to assess the precision of PH-211, PH-249, and PH-251, while (0.005, 0.02, and 0.08 mg/mL) for PH-247, within the range of linearity. Each concentration was processed in triplicate on a single day and five uninterrupted days for PH-211, PH-247, PH-249, and PH-251 to determine the intra-day and inter-day precision of the developed method, respectively, by calculating the relative standard deviation percentage (%RSD).

#### 3.3.3. Extraction Recovery and Matrix Effect

For PH-211, PH-249, and PH-251, two sets of standards containing 0.01, 0.02, 0.04, 0.08, and 0.1 mg/mL of the compound were set. One set was arranged in human plasma and the other set in the mobile phase. While for PH-247, two sets of standards containing 0.005, 0.01, 0.02, 0.04, and 0.08 mg/mL of the compound were prepared. One set was arranged in human plasma and the other set in the mobile phase. The plasma standards were blended with 0.2 mg/mL of internal standard and extracted as mentioned previously, while the standards of the other set, which represent 100% recovery, were directly injected after mixing with internal standard (non-extracted samples). The extraction recoveries were indicated based on the slopes of the standard curves of the compounds in the human plasma and in the mobile phase. Moreover, absolute recoveries of the compounds and internal standard were indicated by comparing the absolute values of the relative peak areas of the compounds and internal standard in extracted and non-extracted samples.

#### 3.3.4. Evaluation of PH-211, PH-247, PH-249, and PH-251 Extraction and Stability in Human Plasma

A sample composed of 0.2 mg/mL of each compound separately was spiked in human plasma and positioned in the oven for 90 min at 37 °C. The sample was then centrifuged and extracted from human plasma by liquid–liquid extraction and mixed with 0.2 mg/mL of the internal standard, followed by filtration by syringe membrane filters (20 µm) kinesis.

#### 3.3.5. Limit of Detection (LOD) and Limit of Quantification (LOQ)

The LOD and LOQ for the compounds were determined at signal-to-noise ratios of 3:1 and 10:1, respectively.

### 3.4. Forced Degradation Studies

Stability tests were performed according to the ICH guidelines to understand how a drug substance or drug product behaves under extreme conditions, beyond normal storage or usage conditions. These studies help to establish the chemical stability profile of the compounds by identifying potential degradation pathways and degradant products. In the current study, stress stability studies were performed under acidic, basic, oxidizing, and elevated temperature conditions. Furthermore, degradants were determined using liquid chromatography–quadrupole time-of-flight mass spectrometry (LC-QToF-MS).

#### 3.4.1. Acidic Degradation

A total of 2 mg of each compound was placed in a vial and dissolved with 1 mL of ACN then exposed to acidic stress surroundings by adding 1 mL of 1 N HCl. The sample was heated at 90 °C for 90 min, cooled for 15 min, and then was processed for analysis and the existence of interfering peak(s) eluted at/or near the retention time of each compound. Degradants were identified by means of LC-QToF-MS.

#### 3.4.2. Basic Degradation

A total of 2 mg of each compound was placed in a vial and dissolved with 1 mL of ACN then subjected to basic stress conditions by adding 1 mL of 1 N NaOH. The sample was heated at 90 °C for 90 min, cooled for 15 min, and then was injected for analysis and the presence of interfering peak(s) eluted at/or near the retention time of each compound. Degradants were identified by means of LC-QToF-MS.

#### 3.4.3. Oxidation Degradation

A total of 2 mg of each compound was placed in a vial and dissolved with 1 mL of ACN then subjected to oxidative stress conditions by adding 1 mL of 1 N H_2_O_2_. The sample was heated at 90 °C for 90 min, cooled for 15 min, and then was injected for analysis and the presence of interfering peak(s) eluted at/or near the retention time of each compound. Degradants were identified by means of LC-QToF-MS.

#### 3.4.4. Thermal Degradation

A total of 2 mg of each compound was placed in a vial and dissolved with 1 mL of ACN then subjected to thermal stress conditions by heating the sample at 90 °C for 90 min, cooled for 15 min, and then was processed for analysis and the existence of interfering peak(s) eluted at/or near the retention time of each compound. Degradants were identified by means of LC-QToF-MS.

## 4. Conclusions

The innovative oxazolidinone hydroxamic acid derivatives (PH-211, PH-247, PH-249, and PH-251) demonstrated potent LT inhibitory activity according to previous studies. Two of these derivatives (PH-211 and PH-251) and the IS were synthesized in this reported study. The structural elucidation was conducted using spectroscopic methods, such as IR, NMR, LC-MS, and an elemental CHN analysis. TLC was used to check their purity. In case further purification was needed, recrystallization or column chromatography was performed depending on the state of the final product.

In addition, this study presents simple, rapid, and reliable UHPLC-UV methods established for the quantification and qualification of these new compounds. These methods, validated according to the ICH guidelines, were used to determine the stability of the compounds in human plasma and under various forced degradation conditions, such as acidic, basic, oxidative, and thermal conditions. This study concluded that all the compounds were stable in the human plasma with high recovery percentages. Moreover, the compounds were stable under thermal conditions but unstable in acidic, basic, and oxidative degradation conditions. The results of the forced degradation conditions show that all four compounds degraded the same way under acidic degradation conditions, which is the hydrolysis of the amide bond in the hydroxamic acid moiety, giving rise to the same degradation product. While under basic degradation conditions, the main mechanisms were amide hydrolysis and the opening of the oxazolidinone ring. However, ring opening was not seen with PH-211. Moreover, the oxidation of the morpholine ring’s nitrogen was noticed under the oxidation degradation conditions, with further the hydrolysis of the *N*-hydroxyamide bonds except for PH-211, which only underwent the oxidation of the ring nitrogen. Therefore, these slight differences noticed with the PH-211 degradation profile make it a good candidate for further development. These findings also show that our compounds share a similar degradation behavior with linezolid and other oxazolidinone-containing drug molecules to some extent, as they all share a similar chemical structure [28,31]. With regard to the oxazolidinone ring structure, compounds with this ring system were found to degrade through ring opening [32,33].

## Figures and Tables

**Table 1:** Chemical structures, molecular weights, and formulas of zileuton and representative novel oxazolidinone hydroxamic acid derivatives.

Compound ID	Structure	Mol. Wt.	Mol. Form.
Zileuton	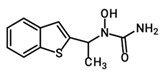	236.29	C_11_H_12_FN_2_O_2_S
PH-211	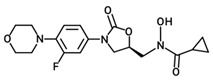	379.38	C_18_H_22_FN_3_O_5_
PH-247	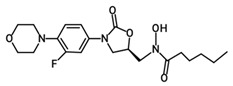	409.46	C_20_H_28_FN_3_O_5_
PH-249	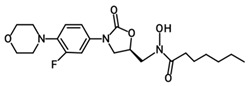	423.49	C_21_H_30_FN_3_O_5_
PH-251	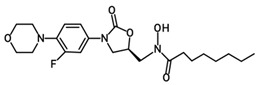	437.51	C_22_H_32_FN_3_O_5_

**Table 2:** Validation parameters of the developed method.

Parameters	PH-211
Range (mg/mL)	0.01–0.1
Regression equation	y = 0.0167x − 0.0006
Correlation coefficient (r)	0.999
LOQ (mg/mL)	0.01
LOD (mg/mL)	0.003
Intra-assay precision ^a^	0.22
Inter-assay precision ^a^	0.16
Accuracy ^b^	100.19

^a^ expressed as the RSD. ^b^ expressed as [mean % deviation = mean calculated relative peak area/reference relative peak area X100].

**Table 3:** Intra-assay precision and accuracy statistics for PH-211 using UHPLC-UV.

PH-211 Concentration mg/mL	Relative Peak Area	Mean ± SD (n = 3) Observed/mg/mL	Precision ^a^ (%)	Accuracy ^b^ (%)
0.01	0.556634	0.557696 ± 0.00125436	0.22	100.19
0.04	2.527021	2.53283 ± 0.005082246	0.20	100.23
0.1	6.010906	6.021317 ± 0.009537771	0.16	100.17

^a^ stated as the RSD. ^b^ expressed as [mean % deviation = mean calculated relative peak area/reference relative peak area X100].

**Table 4:** Inter-assay precision and accuracy statistics for PH-211 using UHPLC-UV.

PH-211 Concentration mg/mL	Relative Peak Area	Mean ± SD (n = 3) Observed/mg/mL	Precision ^a^ (%)	Accuracy ^b^ (%)
0.01	0.541716	0.542095 ± 0.000859498	0.16	100.07
0.04	2.500928	2.502639 ± 0.001872342	0.07	100.07
0.1	5.999658	6.007606 ± 0.011840756	0.20	100.13

^a^ stated as the RSD. ^b^ expressed as [mean % deviation = mean calculated relative peak area/reference relative peak area X100].

**Table 5:** Validation parameters of the developed method.

Parameters	PH-247
Range (mg/mL)	0.005–0.08
Regression equation	y = 0.0452x + 0.0014
Correlation coefficient (r)	0.999
LOQ (mg/mL)	0.005
LOD (mg/mL)	0.0015
Intra-assay precision ^a^	1.35
Inter-assay precision ^a^	1.08
Accuracy ^b^	100.90

^a^ expressed as the RSD. ^b^ expressed as [mean % deviation = mean calculated relative peak area/reference relative peak area X100].

**Table 6:** Intra-assay precision and accuracy data for PH-247 findings using UHPLC-UV.

PH-247 Concentration mg/mL	Relative Peak Area	Mean ± SD (n = 3) Observed/mg/mL	Precision ^a^ (%)	Accuracy ^b^ (%)
0.005	0.088609	0.089403 ± 0.001205543	1.35	100.90
0.02	0.405741	0.402656 ± 0.002881902	0.72	99.24
0.08	1.744349	1.724051 ± 0.018591924	1.08	98.84

^a^ stated as the RSD. ^b^ expressed as [mean % deviation = mean calculated relative peak area/reference relative peak area X100].

**Table 7:** Inter-assay precision and accuracy data for PH-247 findings using UHPLC-UV.

PH-247 Concentration mg/mL	Relative Peak Area	Mean ± SD (n = 3) Observed/mg/mL	Precision ^a^ (%)	Accuracy ^b^ (%)
0.005	0.101431	0.100522 ± 0.001089	1.08	99.10
0.02	0.400455	0.402642 ± 0.001895	0.47	100.55
0.08	1.65512	1.663723 ± 0.007615	0.46	100.52

^a^ stated as the RSD. ^b^ expressed as [mean % deviation = mean calculated relative peak area/reference relative peak area X100].

**Table 8:** Validation parameters of the developed method.

Parameters	PH-249
Range (mg/mL)	0.01–0.1
Regression equation	y = 0.0439x − 0.0001
Correlation coefficient (r)	0.999
LOQ (mg/mL)	0.01
LOD (mg/mL)	0.003
Intra-assay precision ^a^	0.13
Inter-assay precision ^a^	0.19
Accuracy ^b^	100.15

^a^ stated as the RSD. ^b^ expressed as [mean % deviation = mean calculated relative peak area/reference relative peak area X100].

**Table 9:** Intra-assay precision and accuracy data for PH-249 findings using UHPLC-UV.

PH-249 Concentration mg/mL	Relative Peak Area	Mean ± SD (n = 3) Observed/mg/mL	Precision ^a^ (%)	Accuracy ^b^ (%)
0.01	0.205913	0.206218 ± 0.000266067	0.13	100.15
0.04	0.904915	0.906149 ± 0.001072511	0.12	100.14
0.1	2.26952	2.271416 ± 0.002031025	0.09	100.08

^a^ expressed as the RSD. ^b^ expressed as [mean % deviation = mean calculated relative peak area/reference relative peak area X100].

**Table 10:** Inter-assay precision and accuracy data for PH-249 findings using UHPLC-UV.

PH-249 Concentration mg/mL	Relative Peak Area	Mean ± SD (n = 3) Observed/mg/mL	Precision ^a^ (%)	Accuracy ^b^ (%)
0.01	0.208052	0.208422082 ± 0.00039268	0.19	100.18
0.04	0.895176	0.892203737 ± 0.00645177	0.72	99.67
0.1	2.248628	2.247660945 ± 0.00329549	0.15	99.96

^a^ expressed as the RSD. ^b^ expressed as [mean % deviation = mean calculated relative peak area/reference relative peak area X100].

**Table 11:** Validation parameters of the developed method.

Parameters	PH-251
Range (mg/mL)	0.01–0.1
Regression Equation	y = 0.0226x + 0.0011
Correlation Coefficient (r)	0.999
LOQ (mg/mL)	0.01
LOD (mg/mL)	0.003
Intra-assay precision ^a^	0.17
Inter-assay precision ^a^	0.10
Accuracy ^b^	99.81

^a^ stated as the RSD. ^b^ expressed as [mean % deviation = mean calculated relative peak area/reference relative peak area X100].

**Table 12:** Intra-assay precision and accuracy data for PH-251 findings using UHPLC-UV.

PH-251 Concentration mg/mL	Relative Peak Area	Mean ± SD (n = 3) Observed/mg/mL	Precision ^a^ (%)	Accuracy ^b^ (%)
0.01	0.392615	0.391862 ± 0.00066	0.17	99.81
0.04	1.692602	1.695633 ± 0.003542	0.21	100.18
0.1	4.370119	4.369349 ± 0.001657	0.04	99.98

^a^ stated as the RSD. ^b^ expressed as [mean % deviation = mean calculated relative peak area/reference relative peak area X100].

**Table 13:** Inter-assay precision and accuracy data for PH-251 findings using UHPLC-UV.

**PH-251** **Concentration** **mg/mL**	**Relative Peak Area**	**Mean ± SD** **(n = 3)** **Observed/mg/mL**	Precision ^a^ (%)	Accuracy ^b^ (%)
0.01	0.383002	0.382554 ± 0.000389	0.10	99.88
0.04	1.672021	1.684208 ± 0.012462	0.74	100.73
0.1	4.330851	4.337159 ± 0.007248	0.17	100.15

^a^ stated as the RSD. ^b^ expressed as [mean % deviation = mean calculated relative peak area/reference relative peak area X100].

## Data Availability

Data is contained within the article and Supplementary Material.

## Supplementary Materials

The following supporting information can be downloaded at https://www.mdpi.com/article/10.3390/ph19010069/s1.
