# Supplementary material for: Stability-Indicating Assay of Novel 5-(Hydroxamic acid)methyl Oxazolidinones with 5-Lipooxygenase Inhibitory Activity
Source: Pharmaceuticals (Basel). 2025 Dec 29;19(1):69. doi: 10.3390/ph19010069 (PMC12844684; doi:10.3390/ph19010069)
Supplement: Supplementary file 1 [file pharmaceuticals-19-00069-s001.zip › pharmaceuticals-3816371-supplementary.pdf]

Supplementary Materials

File S1 IR

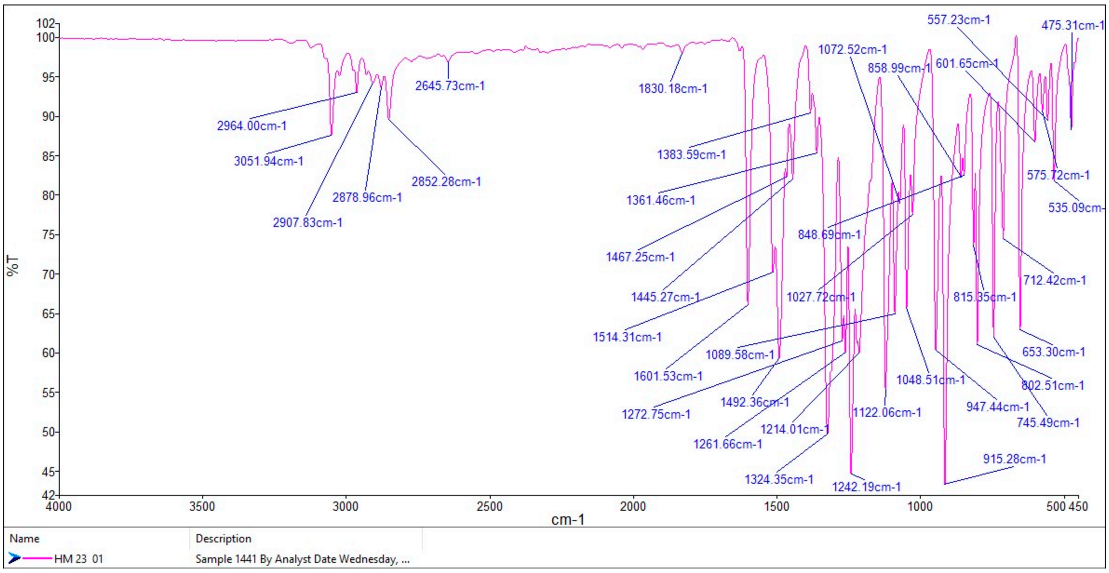

Figure S1: IR spectrum of HM.01

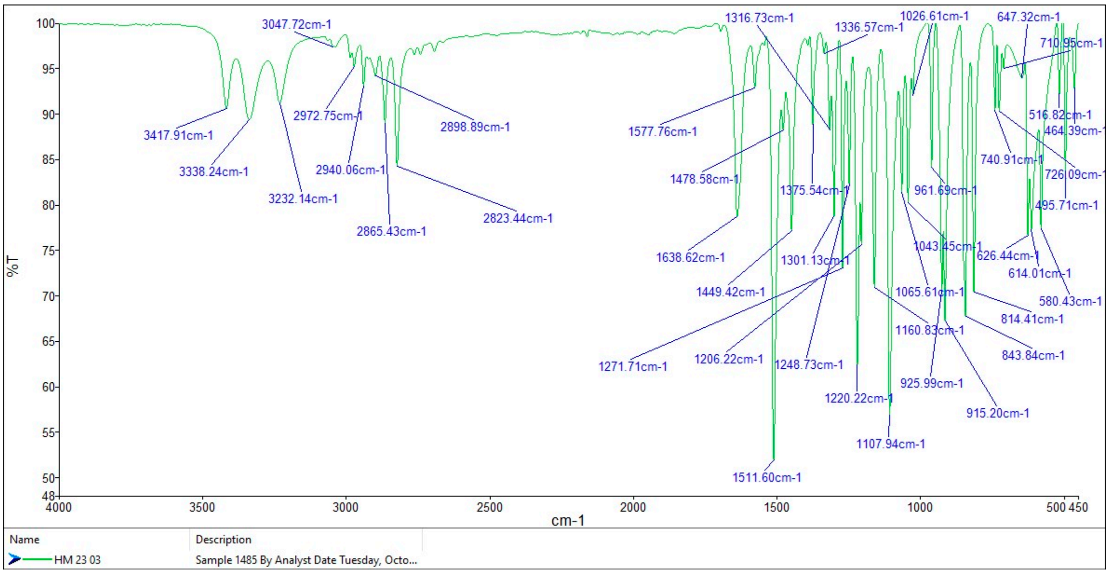

Figure S2: IR spectrum of HM.03

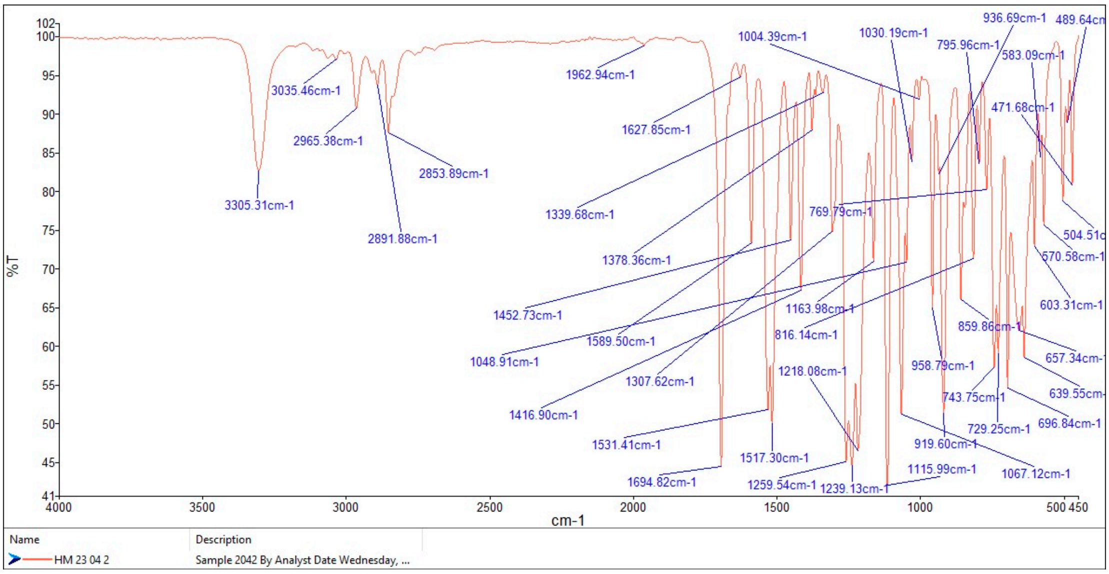

Figure S3: IR spectrum of HM.04

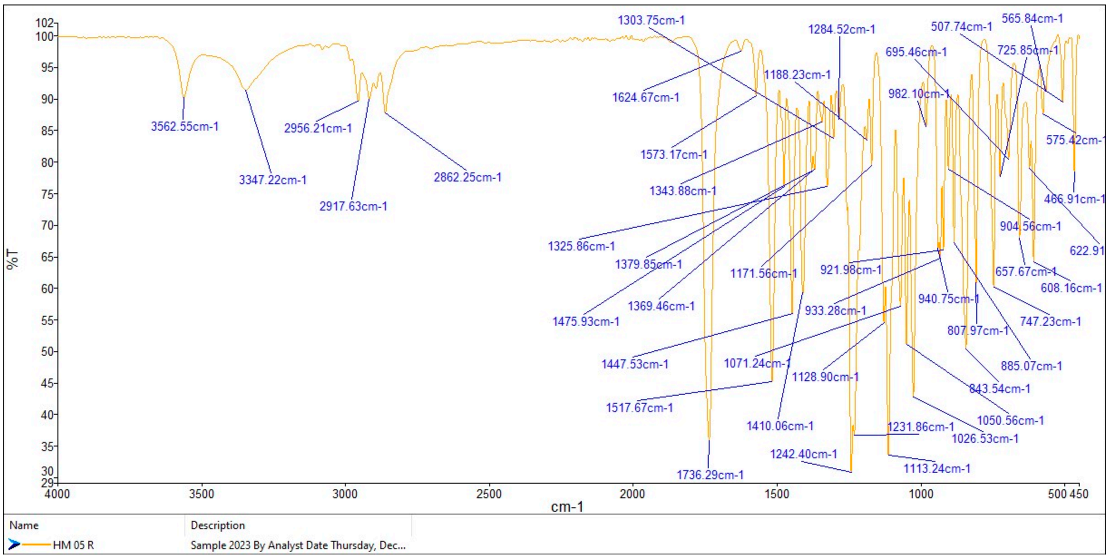

Figure S4: IR spectrum of HM.05

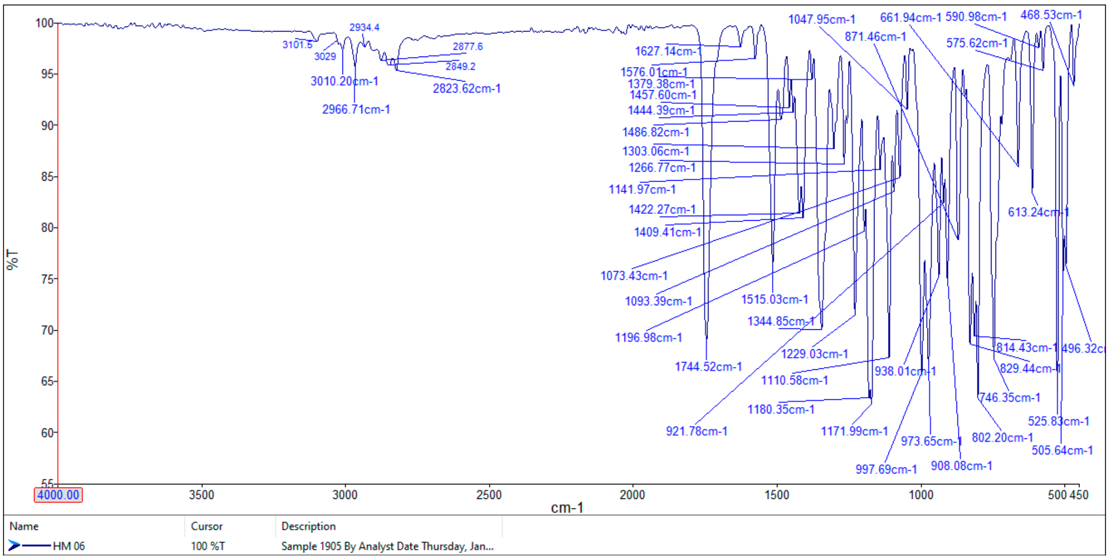

Figure S5: IR spectrum of HM.06

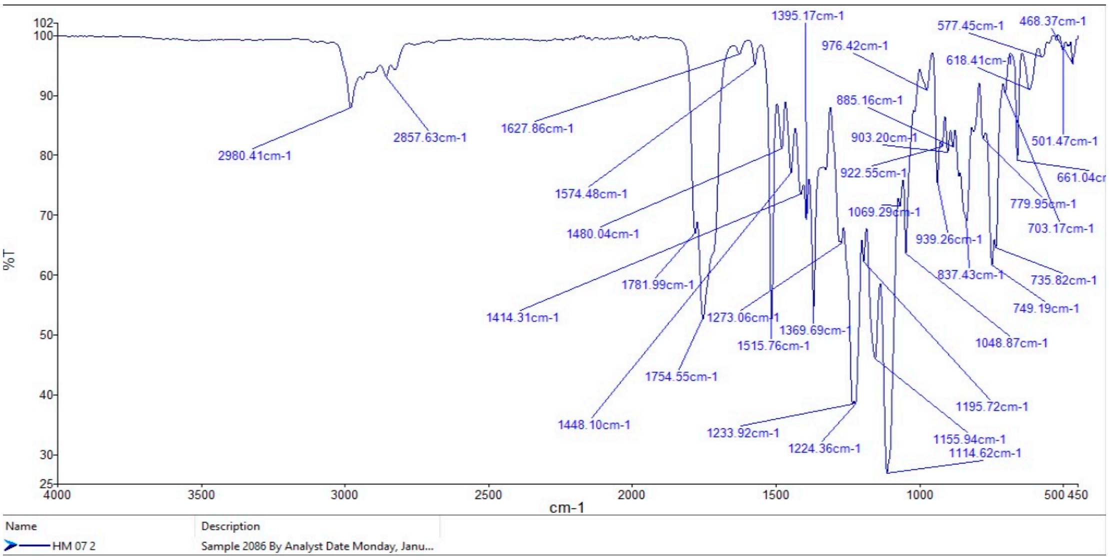

Figure S6: IR spectrum of HM.07

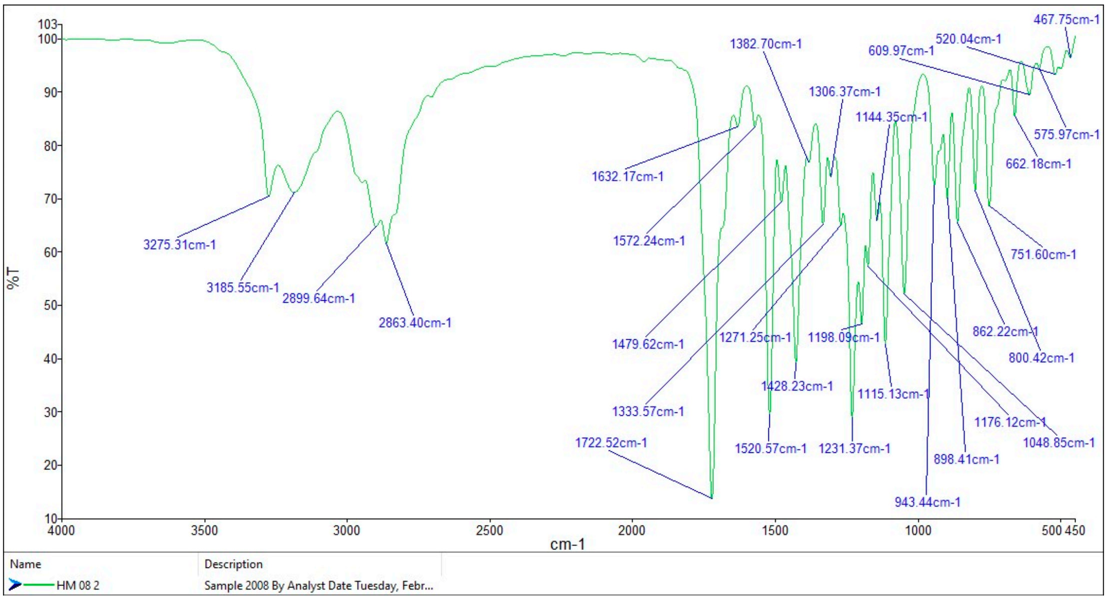

Figure S7: IR spectrum of HM.08

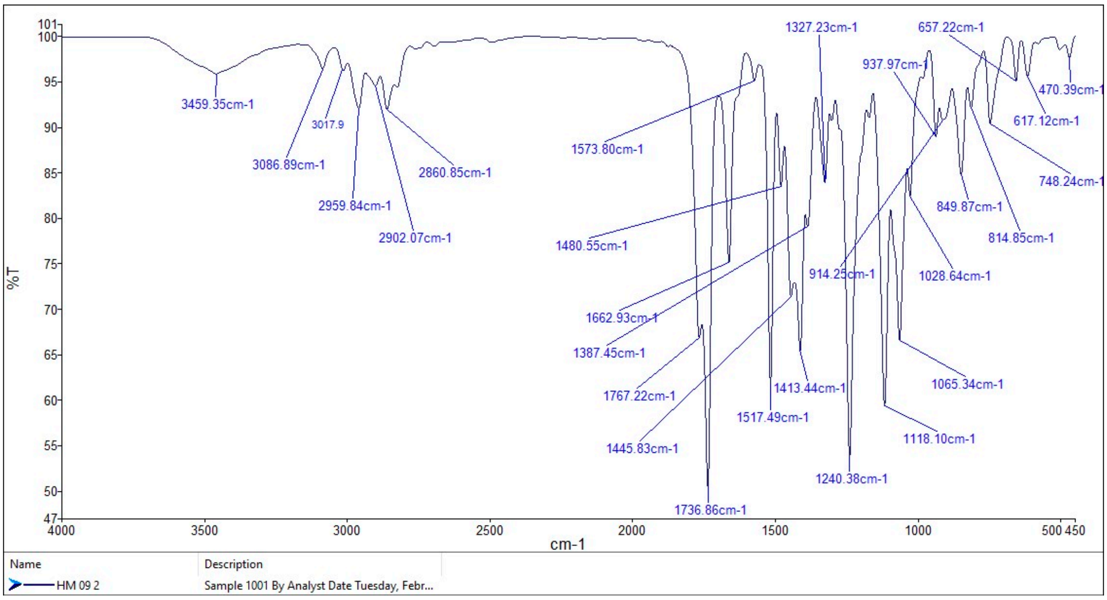

Figure S8: IR spectrum of HM.09

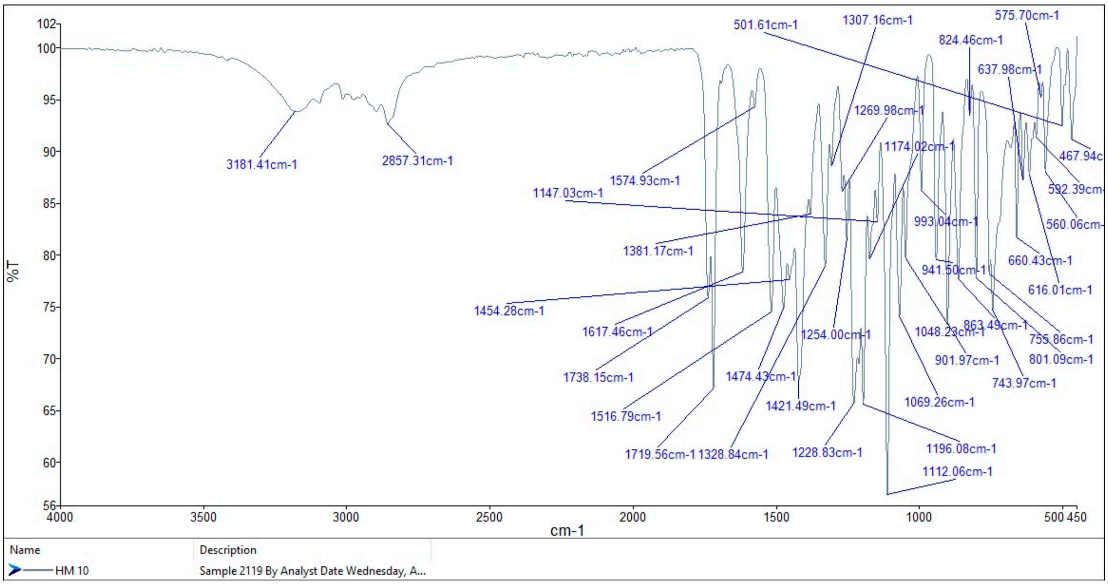

Figure S9: IR spectrum of HM.10

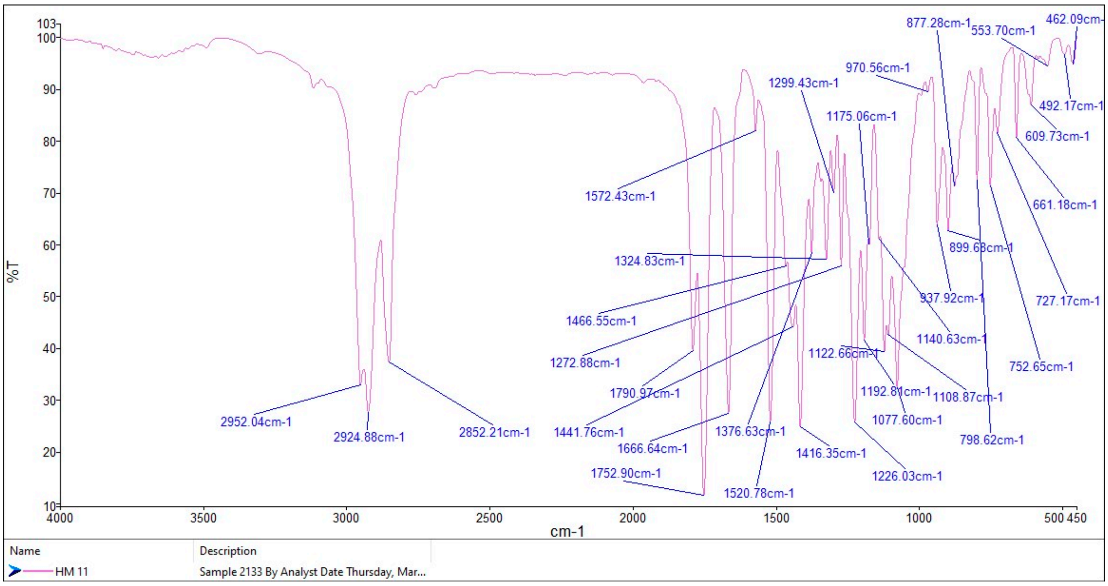

Figure S10: IR spectrum of HM.11

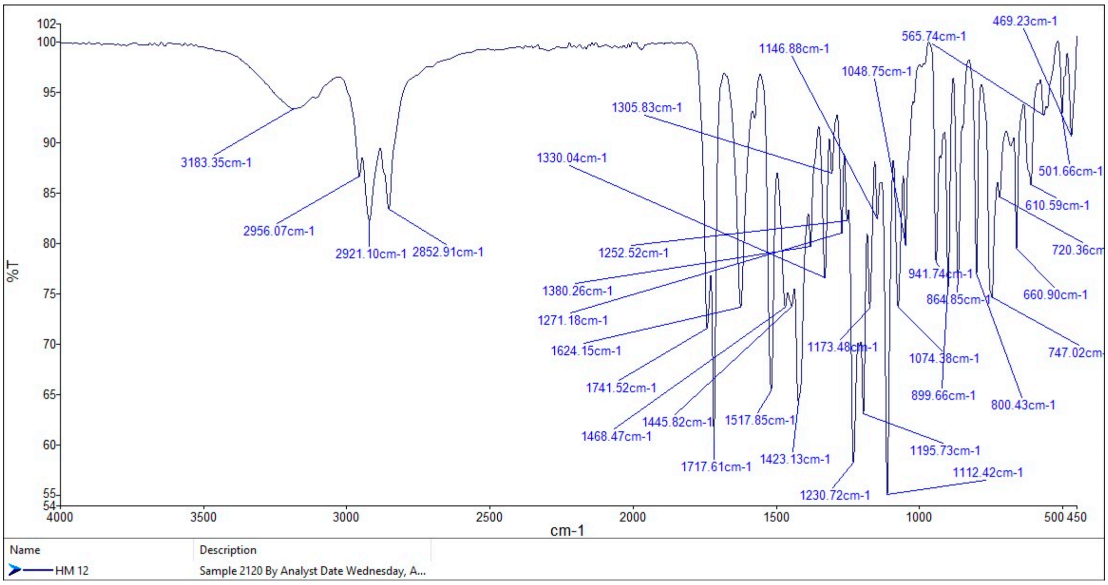

Figure S11: IR spectrum of HM.12

File S2 NMR

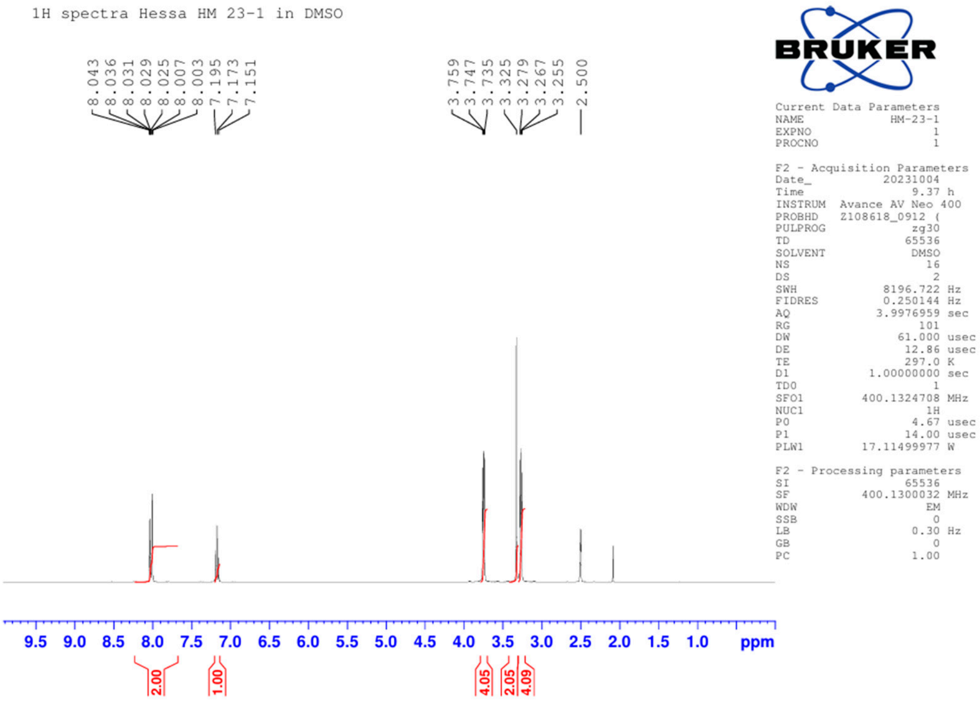

Figure S12: ^{1}H NMR spectrum of HM.01 in DMSO-d6, 400MHz

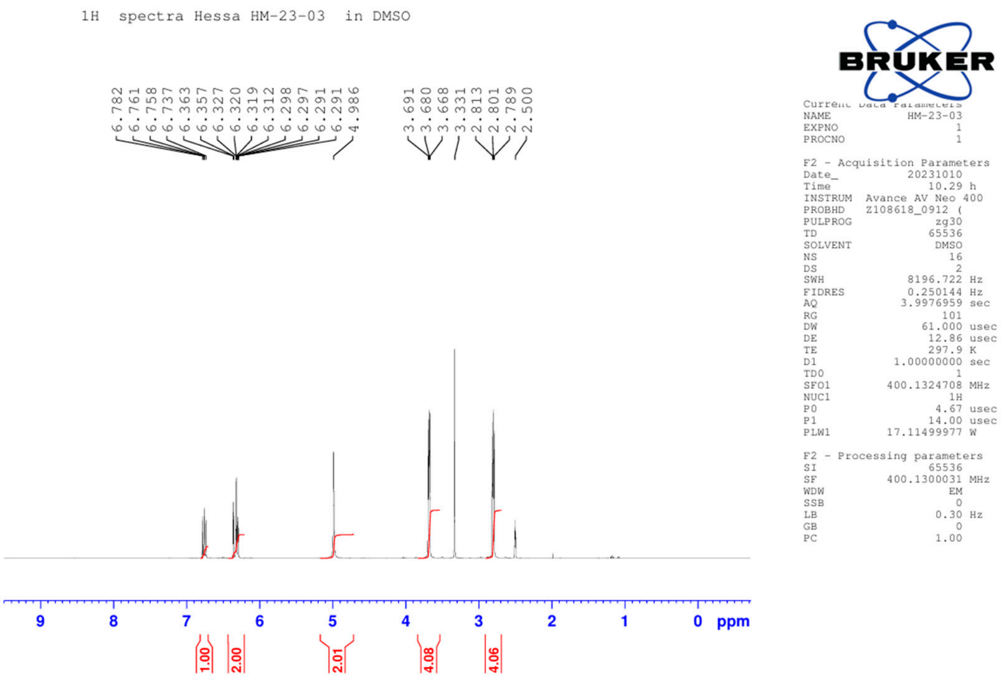

Figure S13: ^{1}H NMR spectrum of HM.03 in DMSO-d6, 400MHz

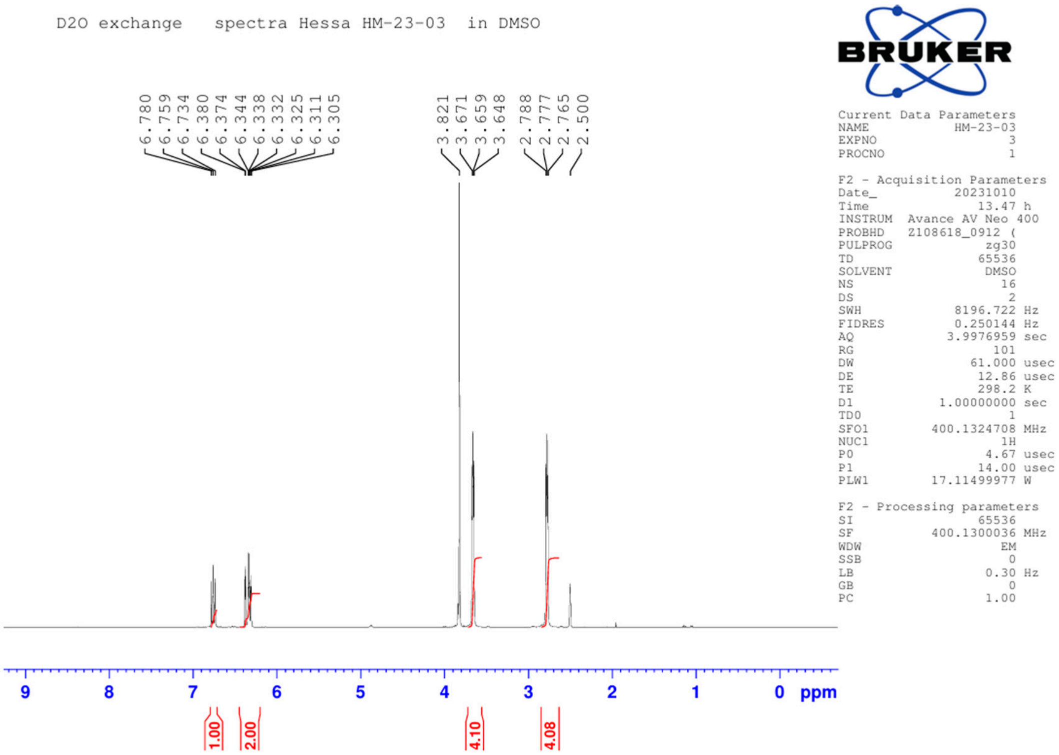

Figure S14: D_2O exchange spectrum of HM.03 in DMSO-d6, 400MHz

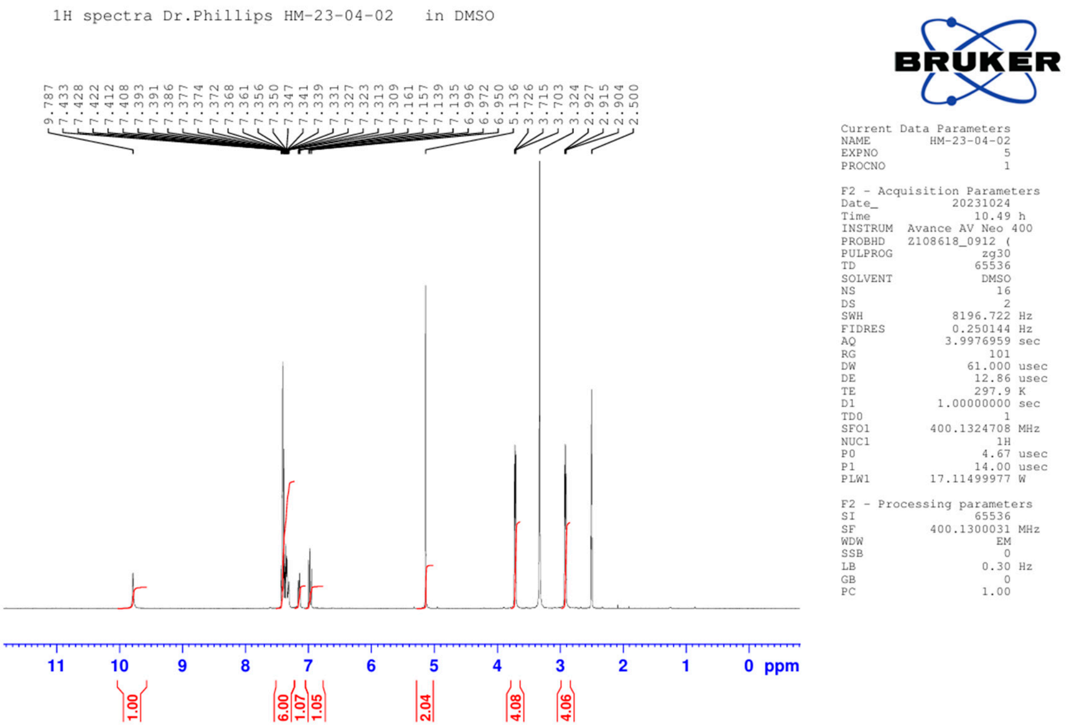

Figure S15: ^{1}H NMR spectrum of HM.04 in DMSO-d6, 400MHz

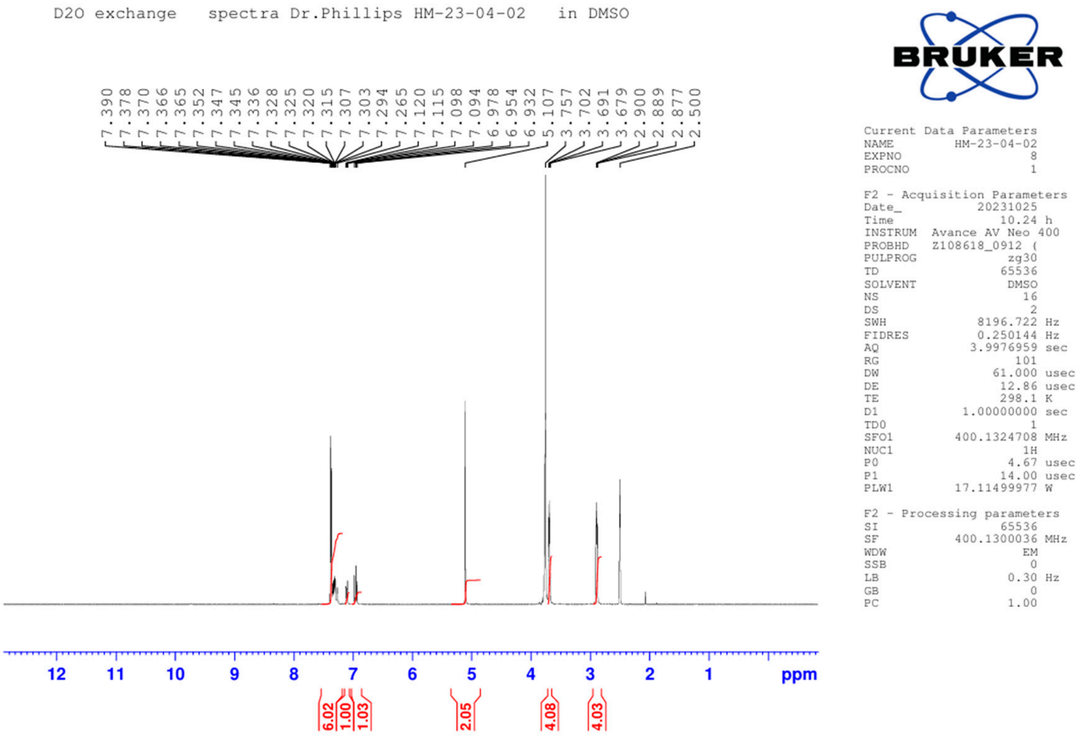

Figure S16: D_2O exchange spectrum of HM.04 in DMSO-d6, 400MHz

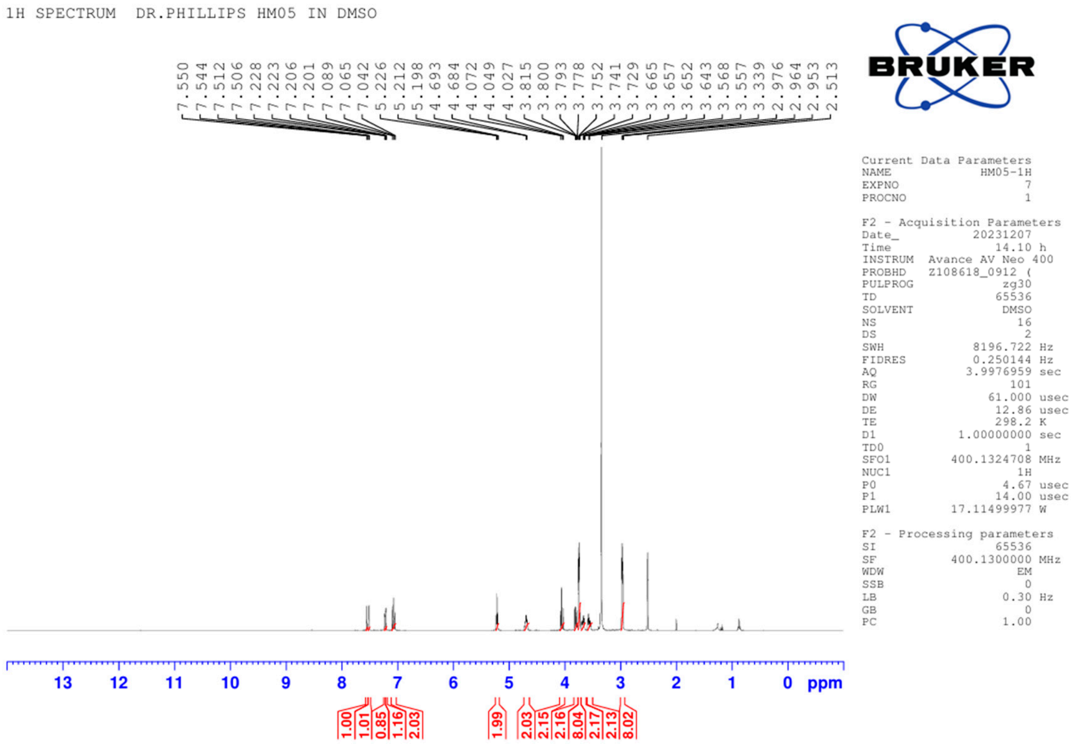

Figure S17: ^{1}H NMR spectrum of HM.05 in DMSO-d6, 400MHz

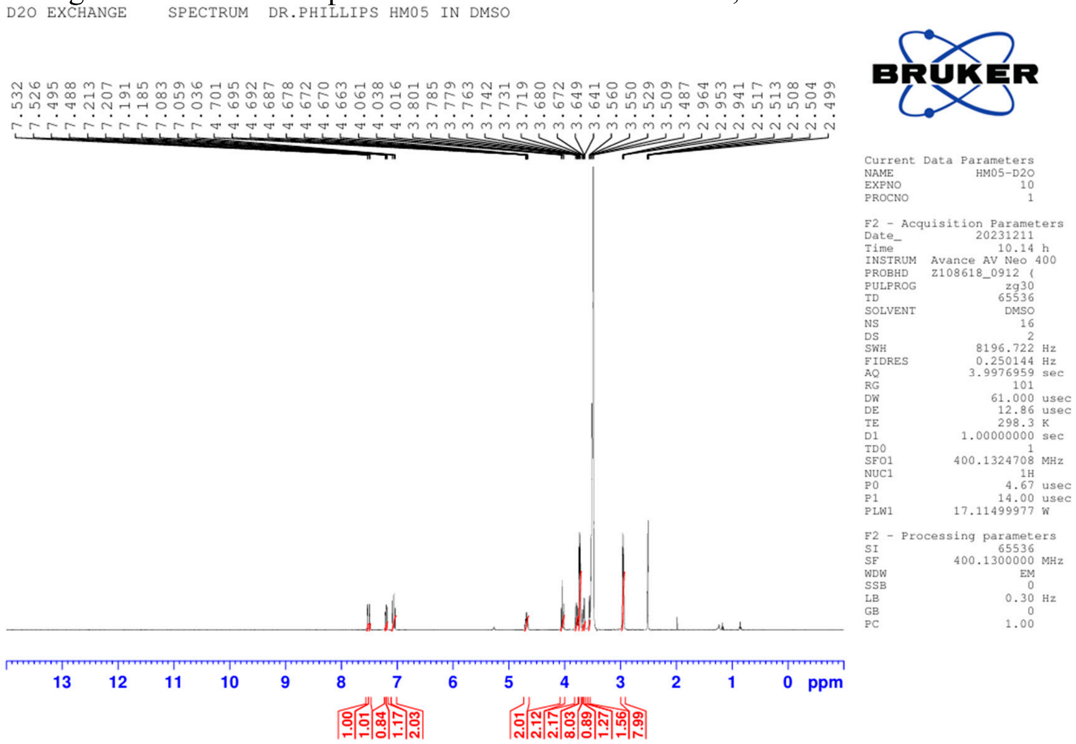

Figure S18: D_2O exchange spectrum of HM.05 in DMSO-d6, 400MHz

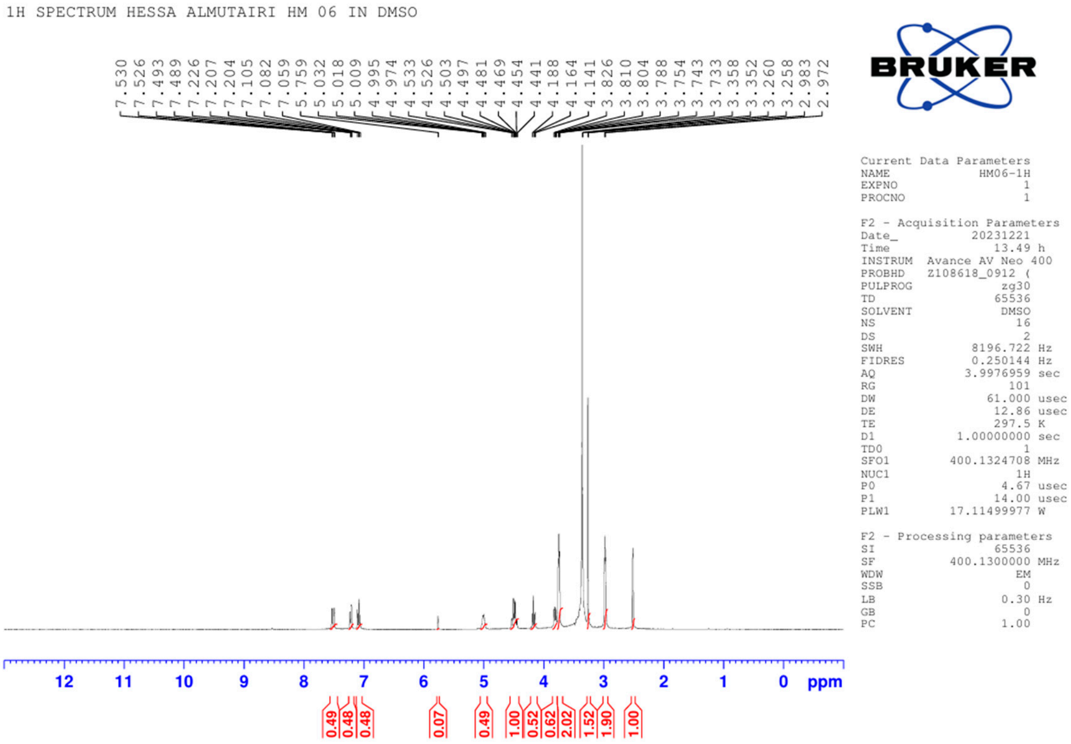

Figure S19: ^{1}H NMR spectrum of HM.06 in DMSO-d6, 400MHz

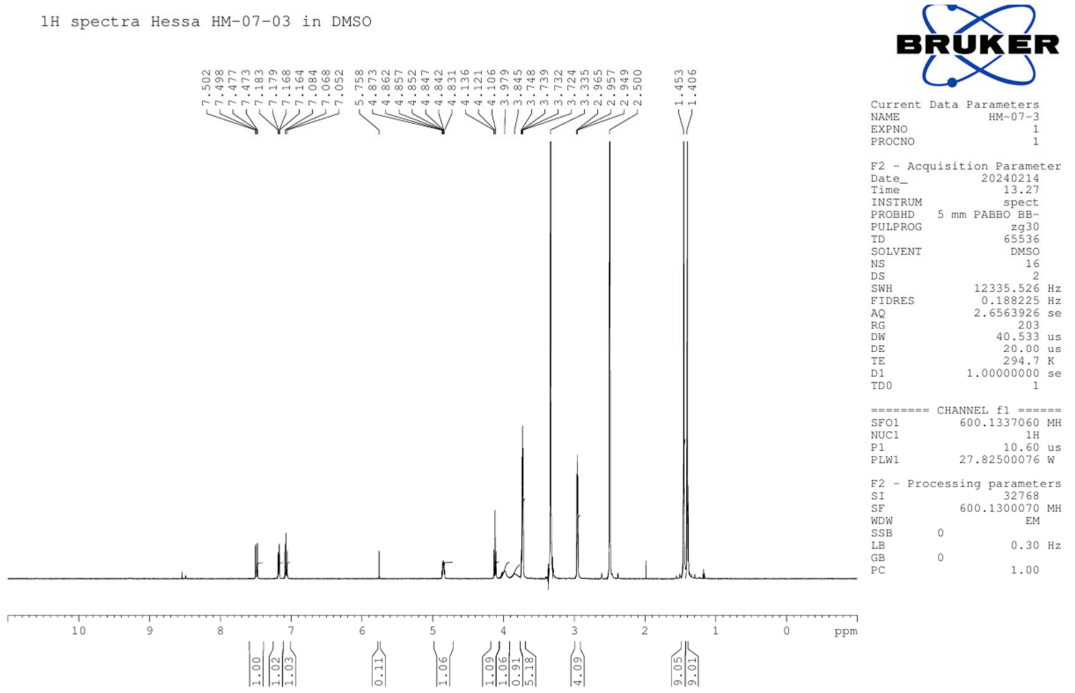

Figure S20: ^{1}H NMR spectrum of HM.07 in DMSO-d6, 600MHz

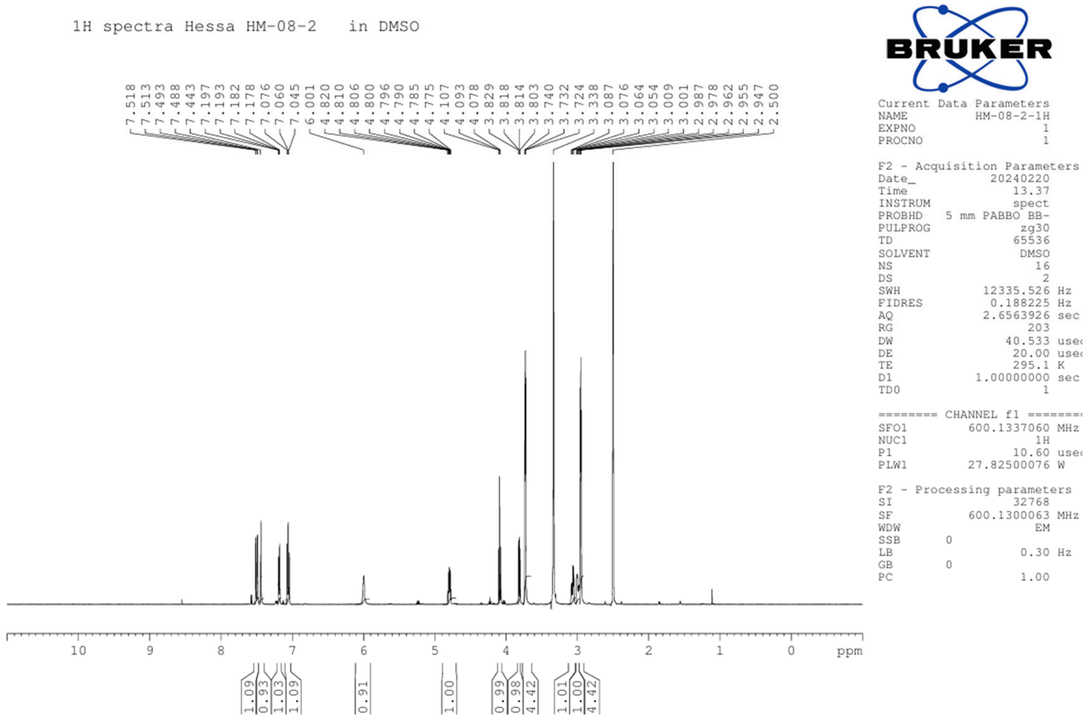

Figure S21: ^{1}H NMR spectrum of HM.08 in DMSO-d6, 600MHz

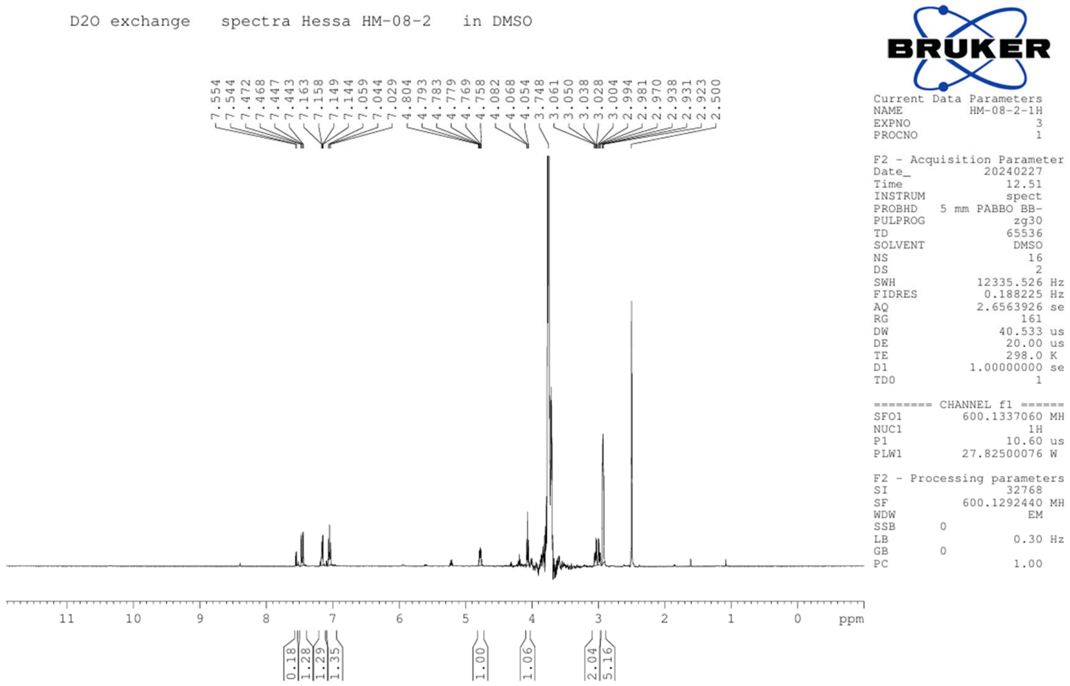

Figure S22: D_2O exchange spectrum of HM.08 in DMSO-d6, 600MHz

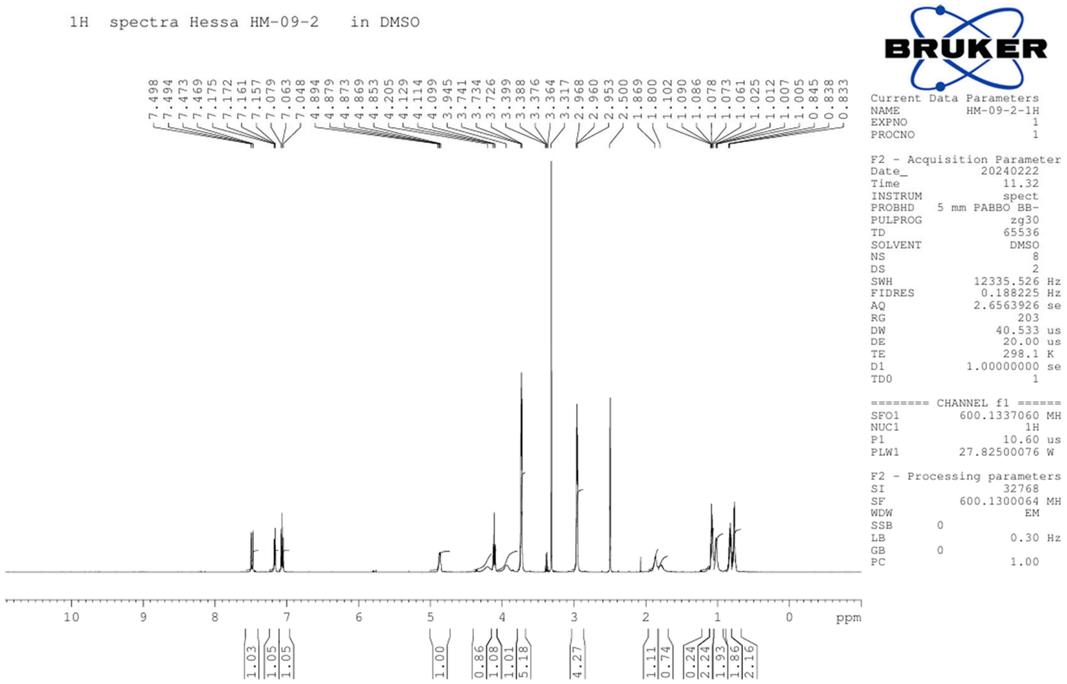

Figure S23: ^{1}H NMR spectrum of HM.09 in DMSO-d6, 600MHz

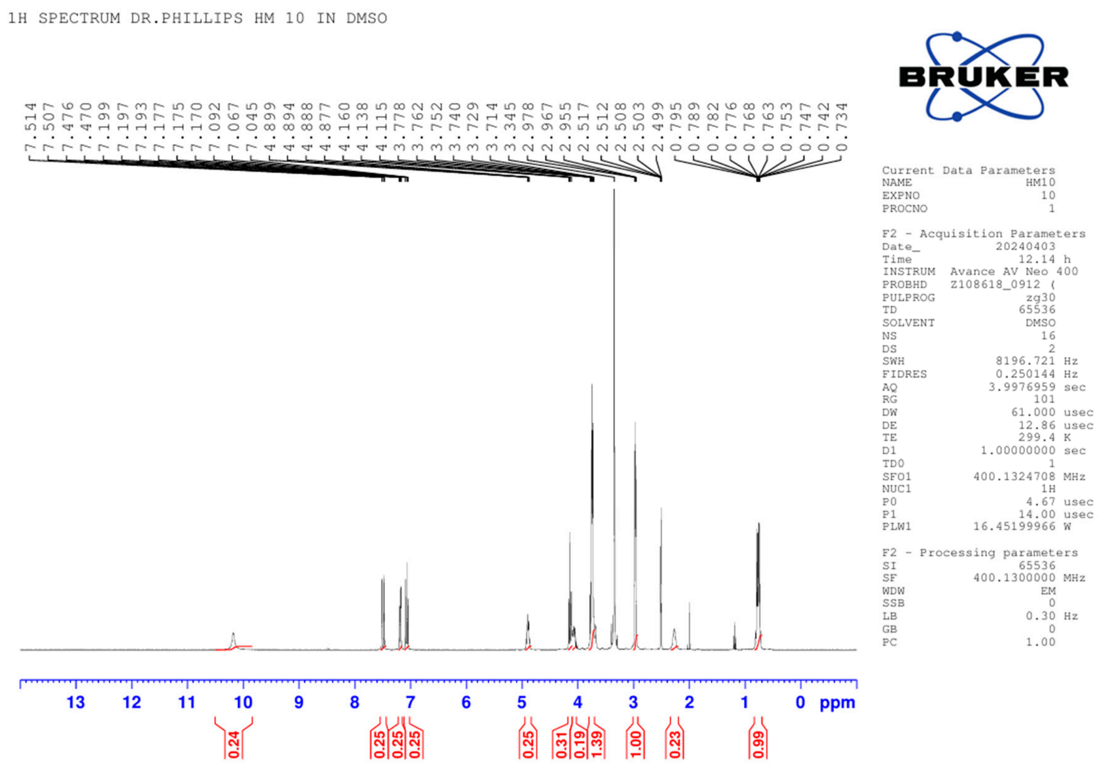

Figure S24: ^{1}H NMR spectrum of HM.10 in DMSO-d6, 400MHz

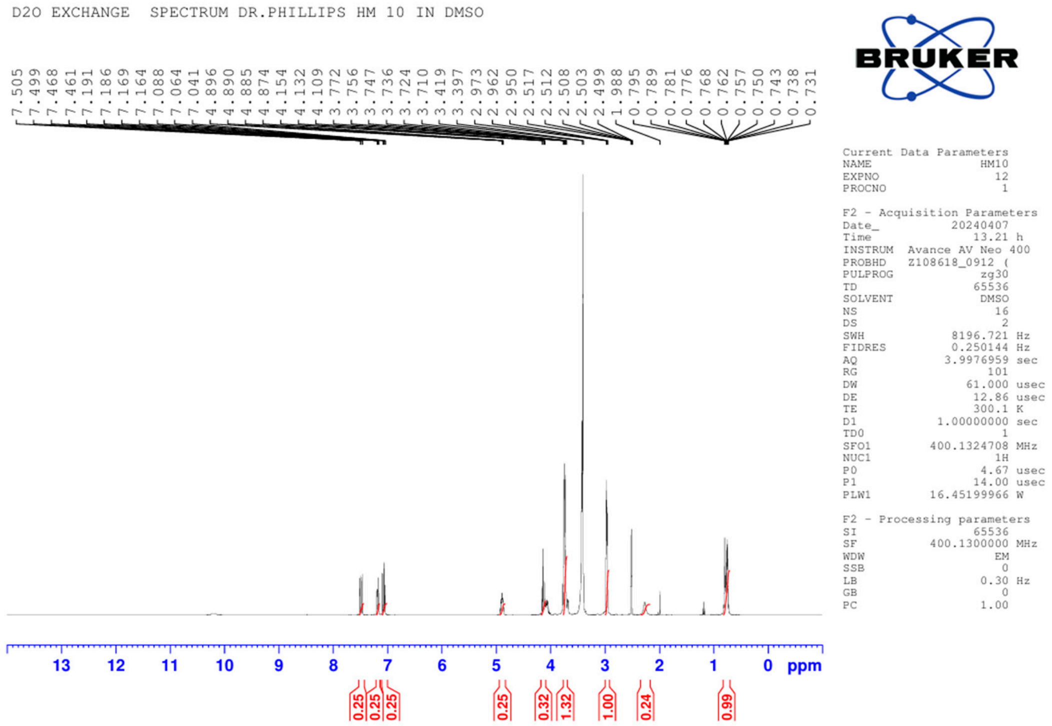

Figure S25: D_2O exchange spectrum of HM.10 in DMSO-d6, 400MHz

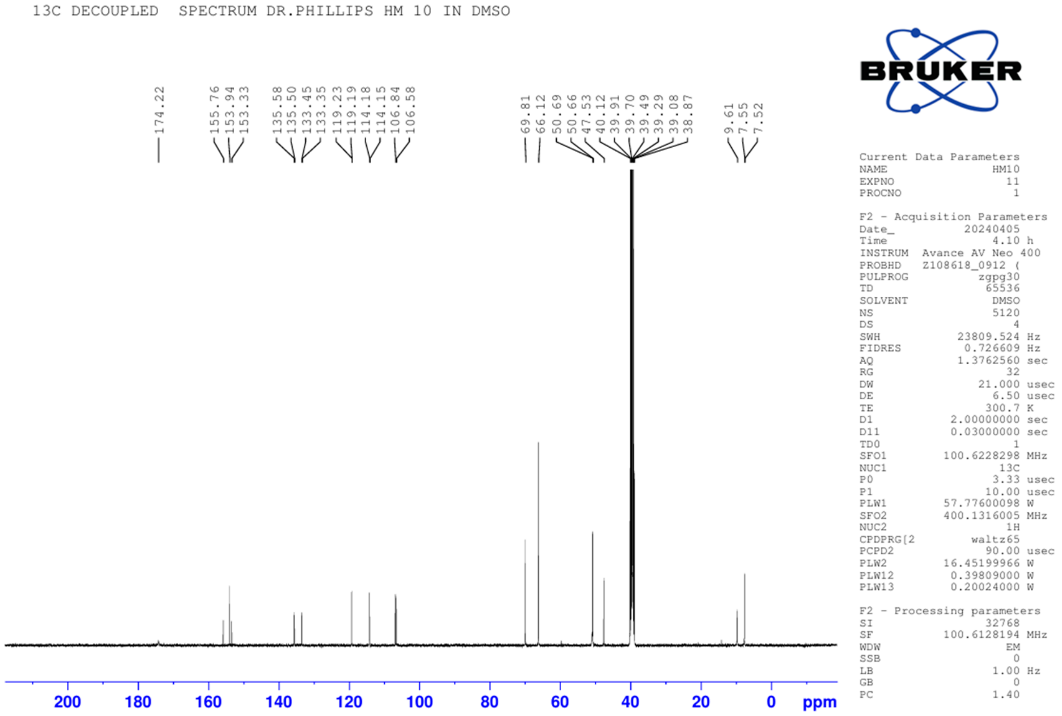

Figure S26: ^{13}C NMR spectrum of HM.10 in DMSO-d6, 400MHz

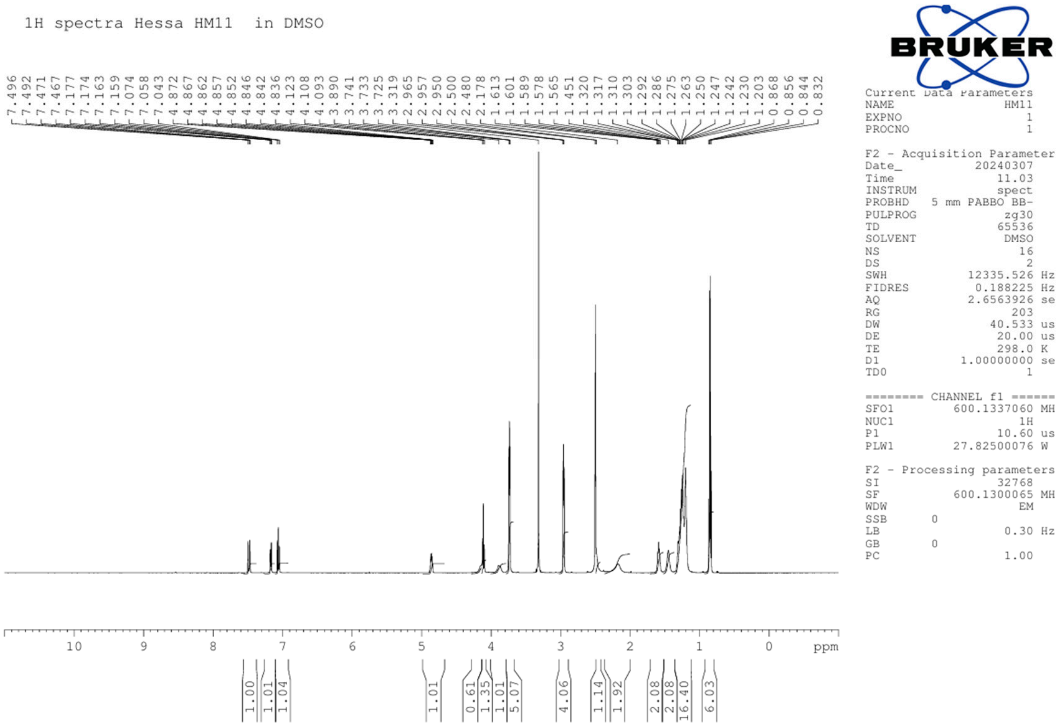

Figure S27: ^{1}H NMR spectrum of HM.11 in DMSO-d6, 600MHz

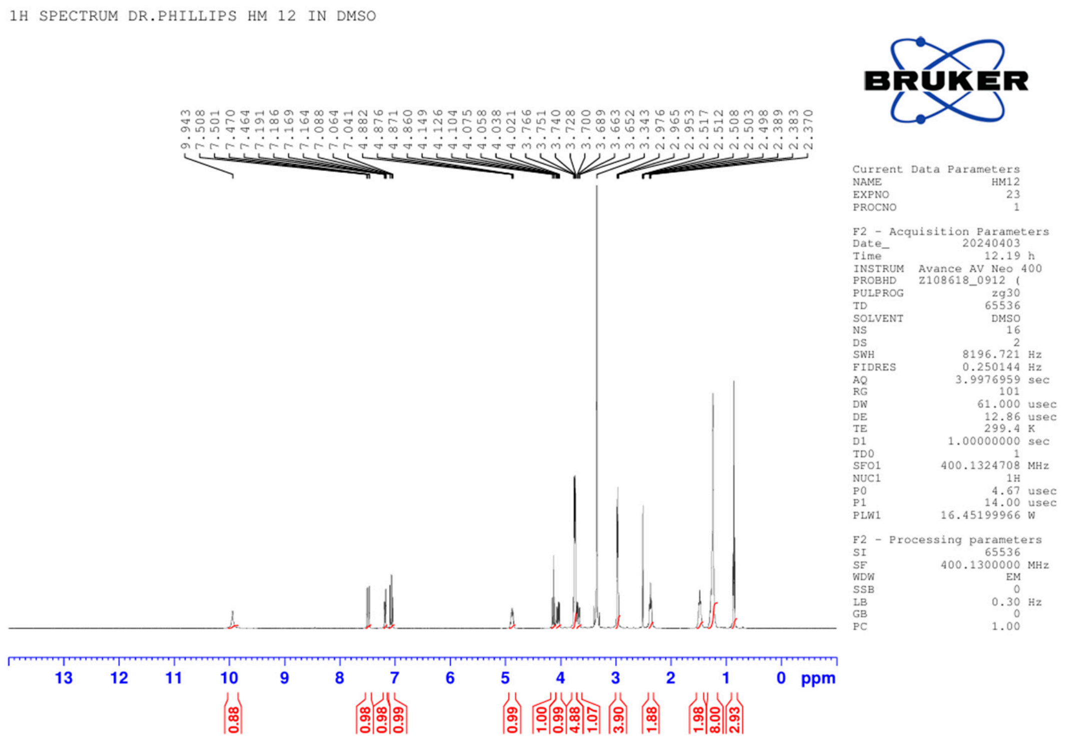

Figure S28: ^{1}H NMR spectrum of HM.12 in DMSO-d6, 400MHz

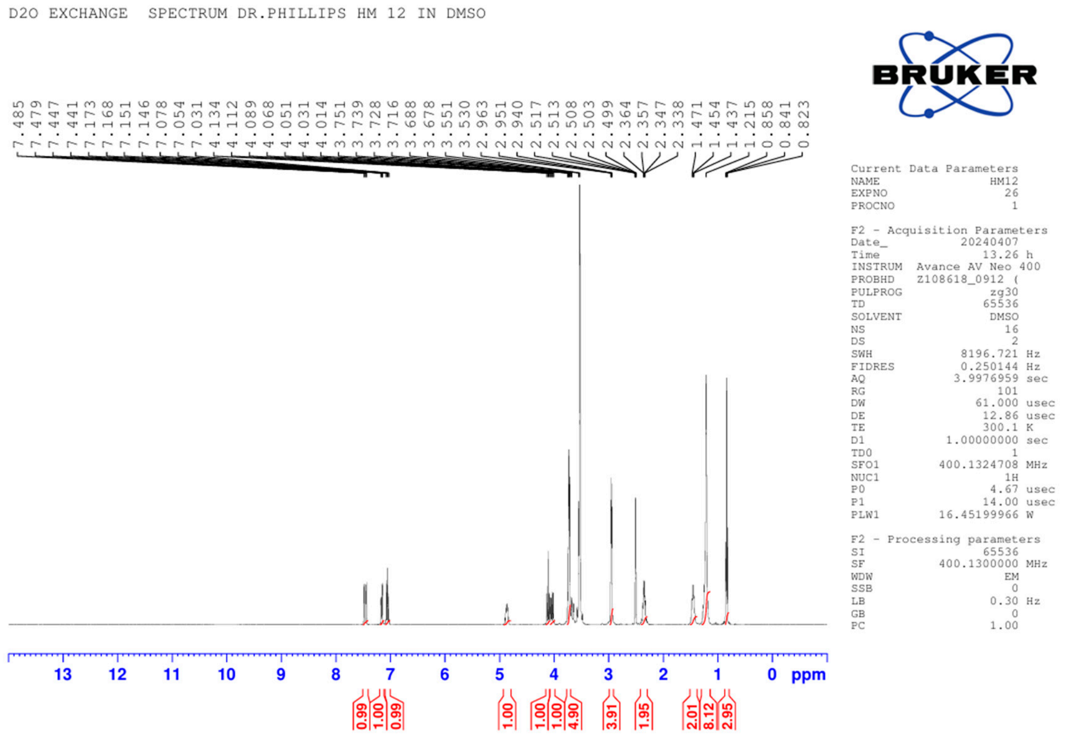

Figure S29: D_2O exchange spectrum of HM.12 in DMSO-d6, 400MHz

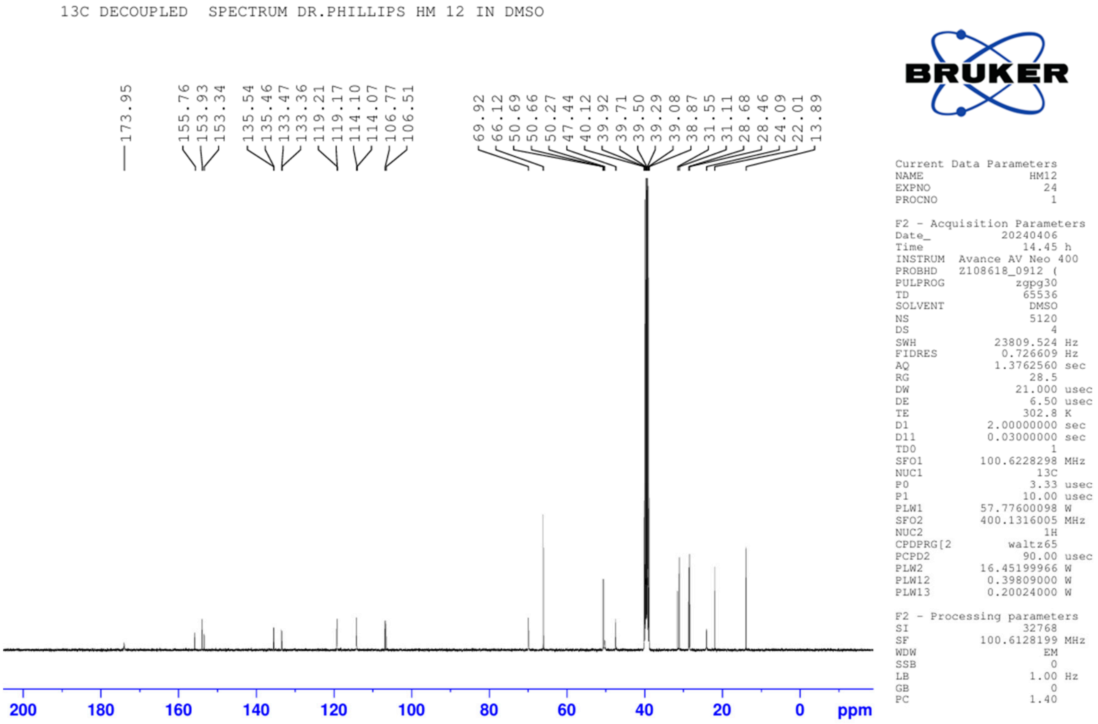

Figure S30: ^{13}C NMR spectrum of HM.12 in DMSO-d6, 400MHz

File S3 LC-MS

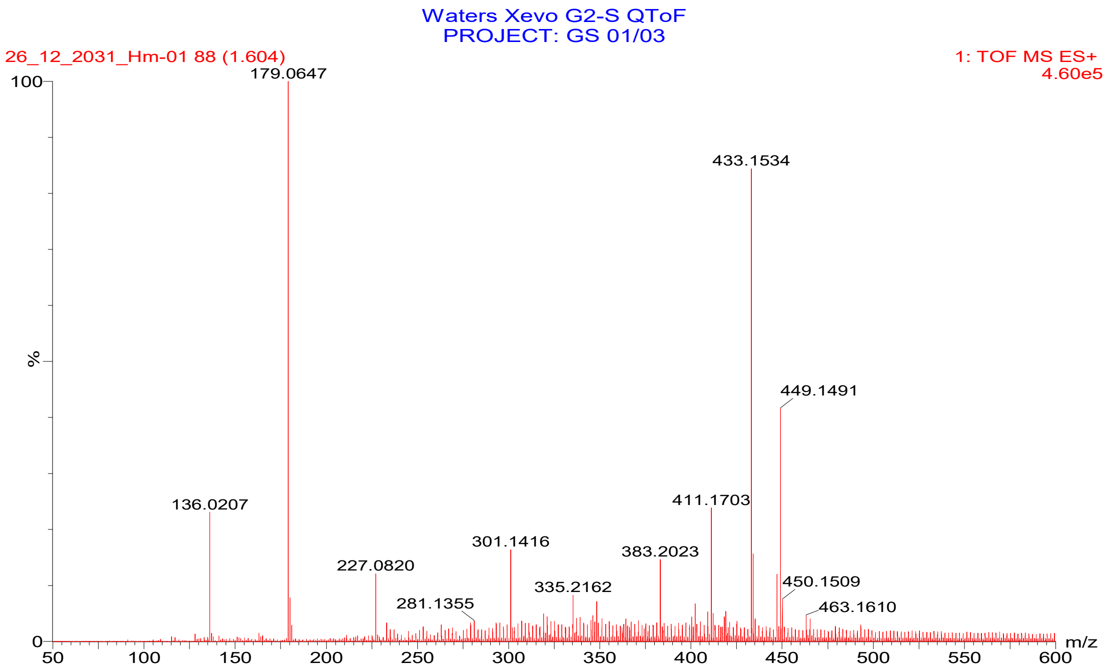

Figure S31: Mass spectrum of HM.01

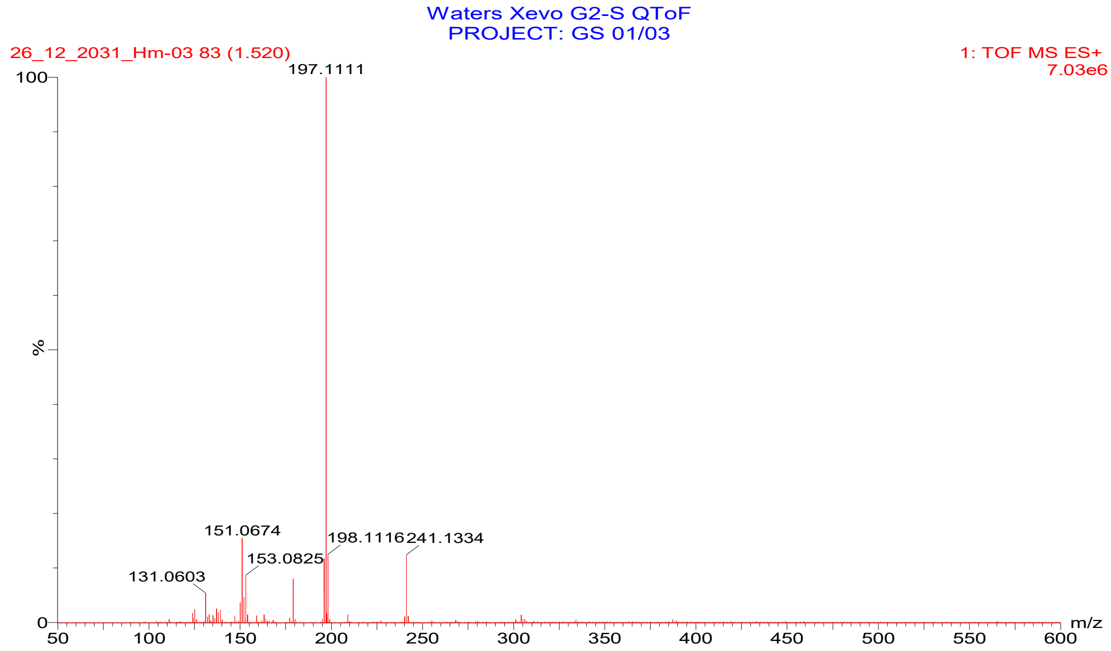

Figure S32: Mass spectrum of HM.03

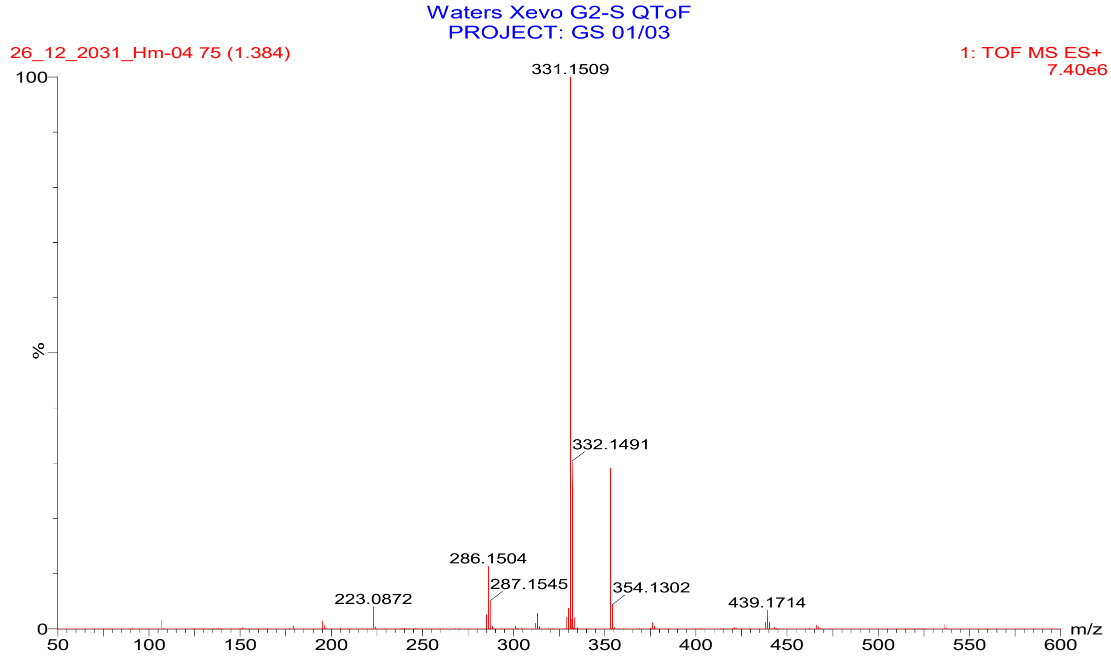

Figure S33: Mass spectrum of HM.04

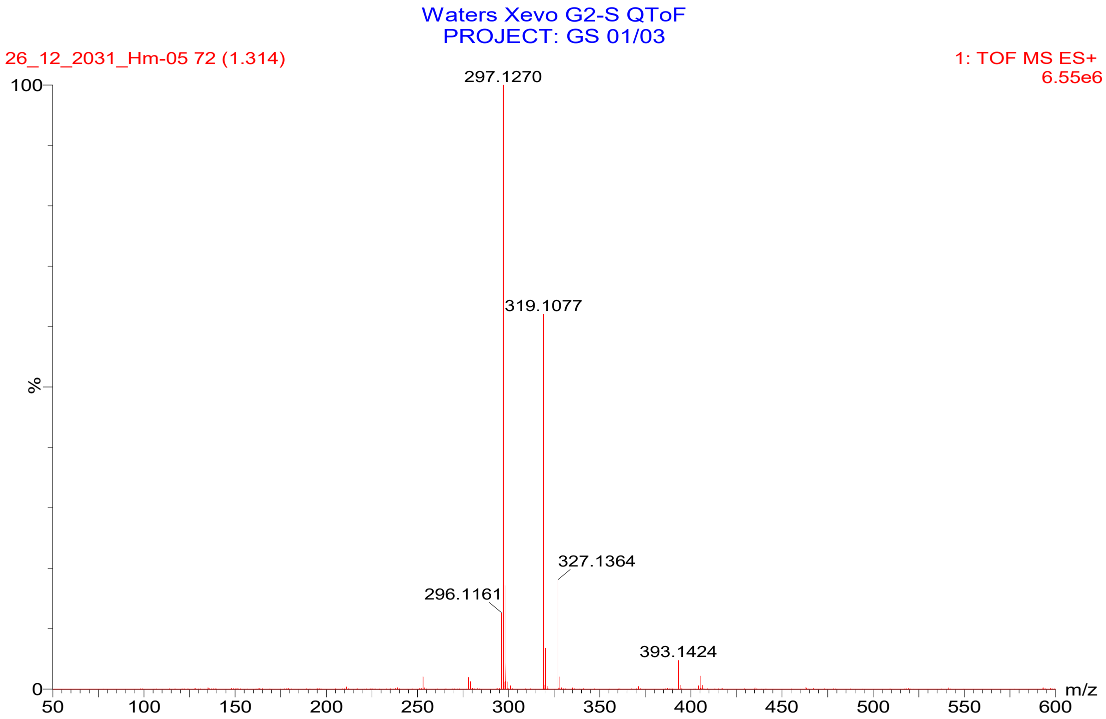

Figure S34: Mass spectrum of HM.05

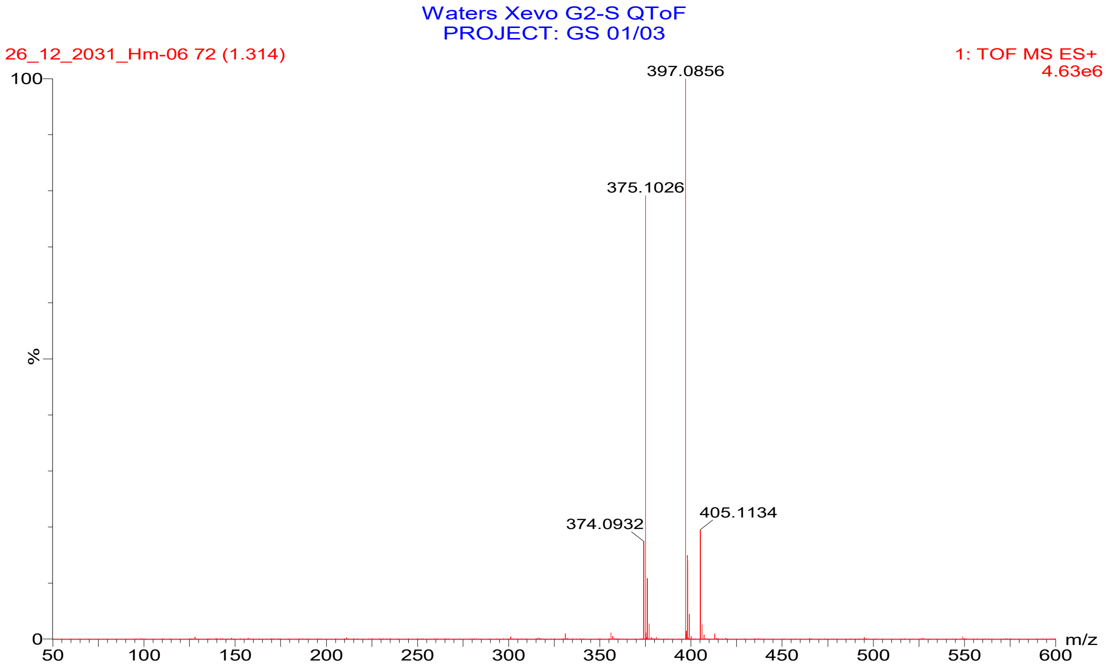

Figure S35: Mass spectrum of HM.06

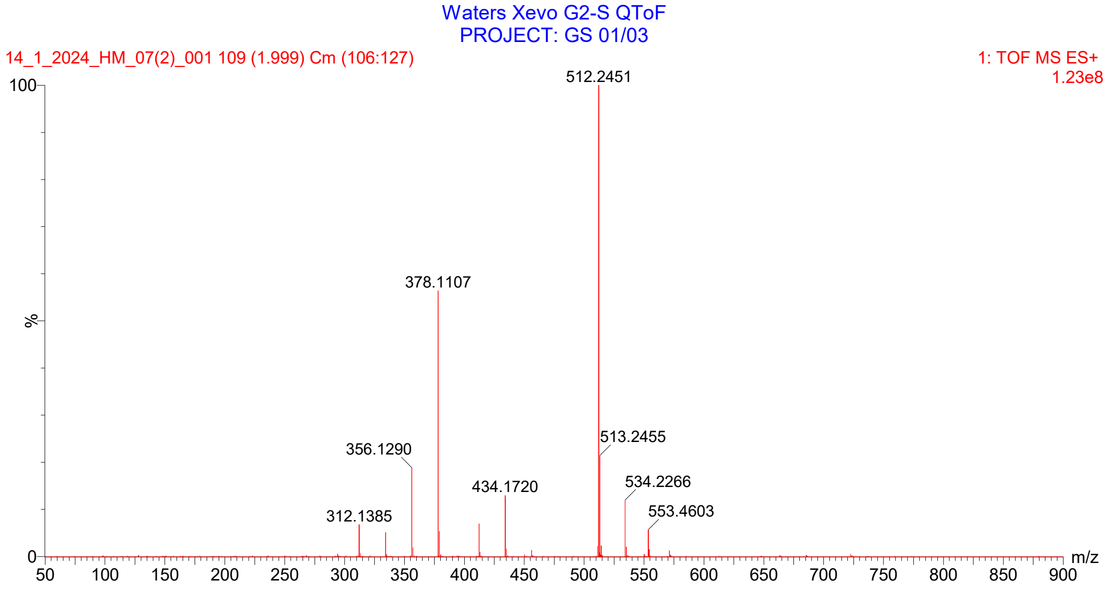

Figure S36: Mass spectrum of HM.07

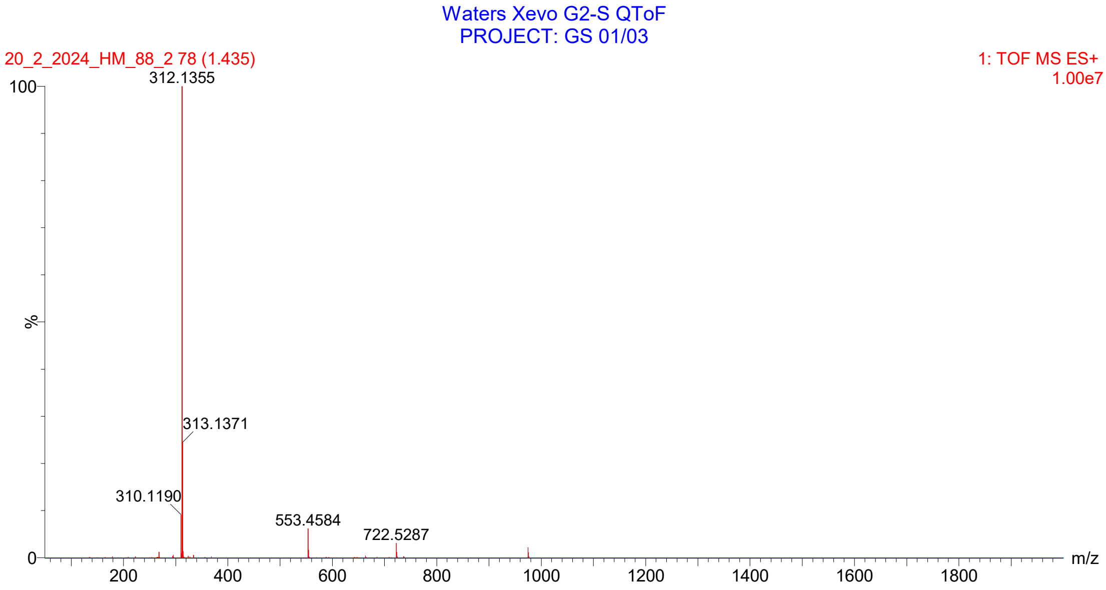

Figure S37: Mass spectrum of HM.08

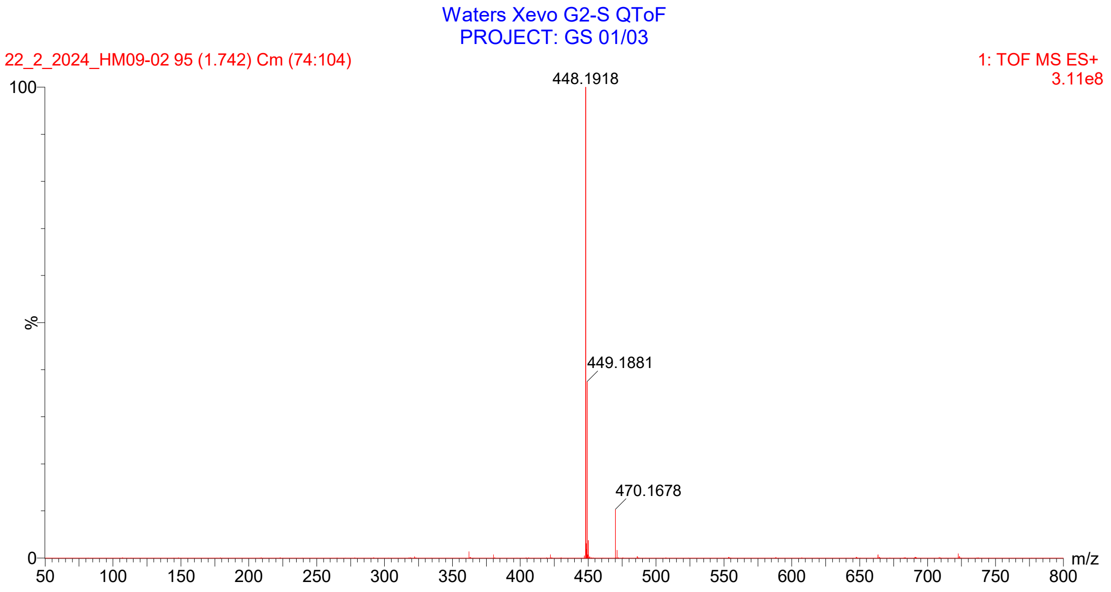

Figure S38: Mass spectrum of HM.09

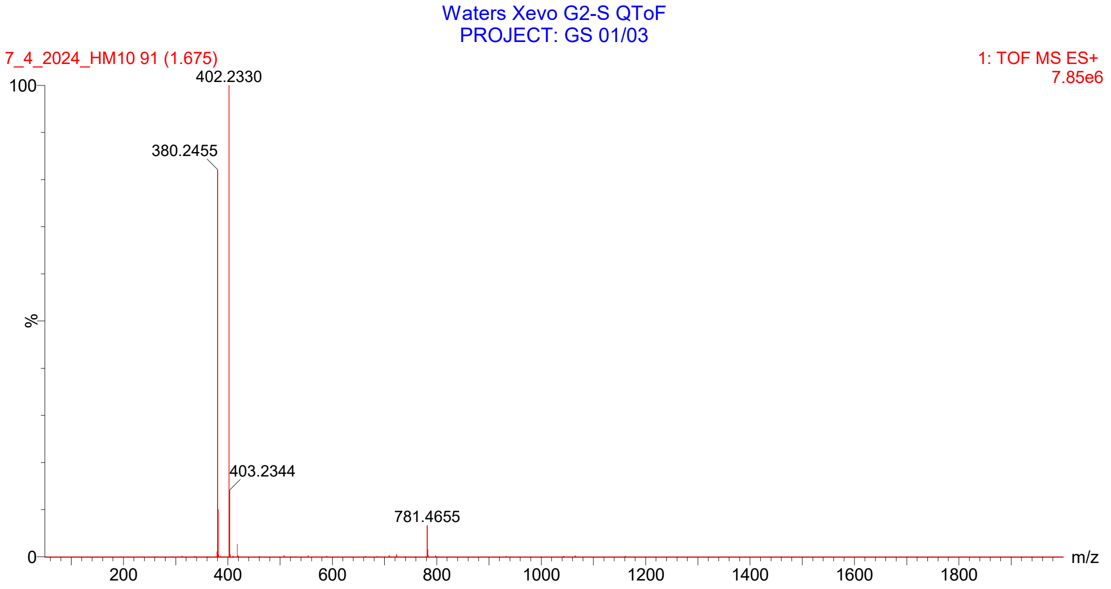

Figure S39: Mass spectrum of HM.10

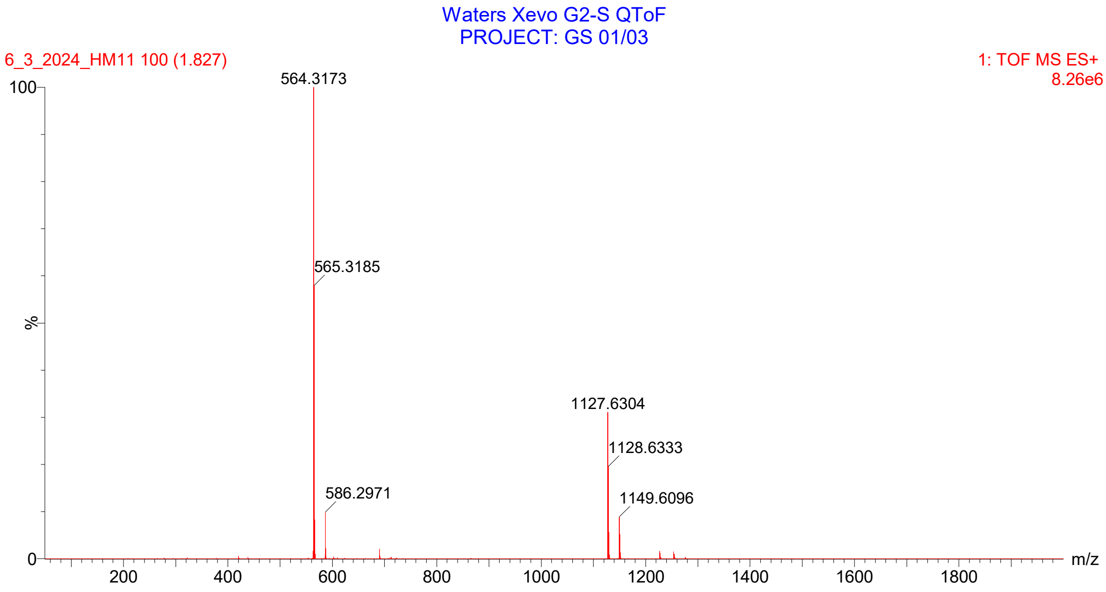

Figure S40: Mass spectrum of HM.11

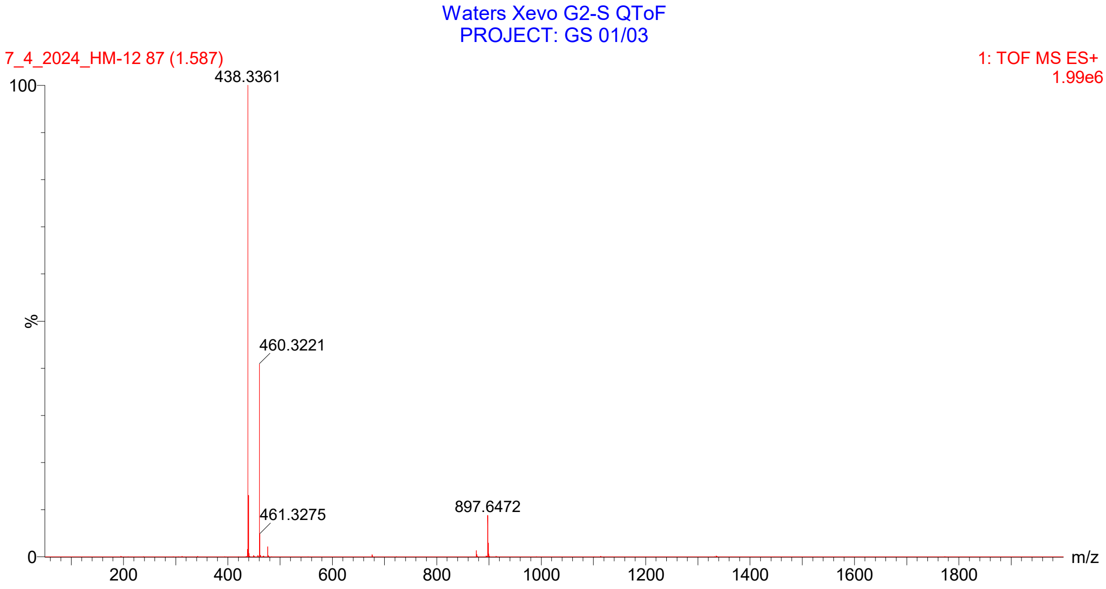

Figure S41: Mass spectrum of HM.12

File S4 UHPLC-UV chromatograms of the calibration curves

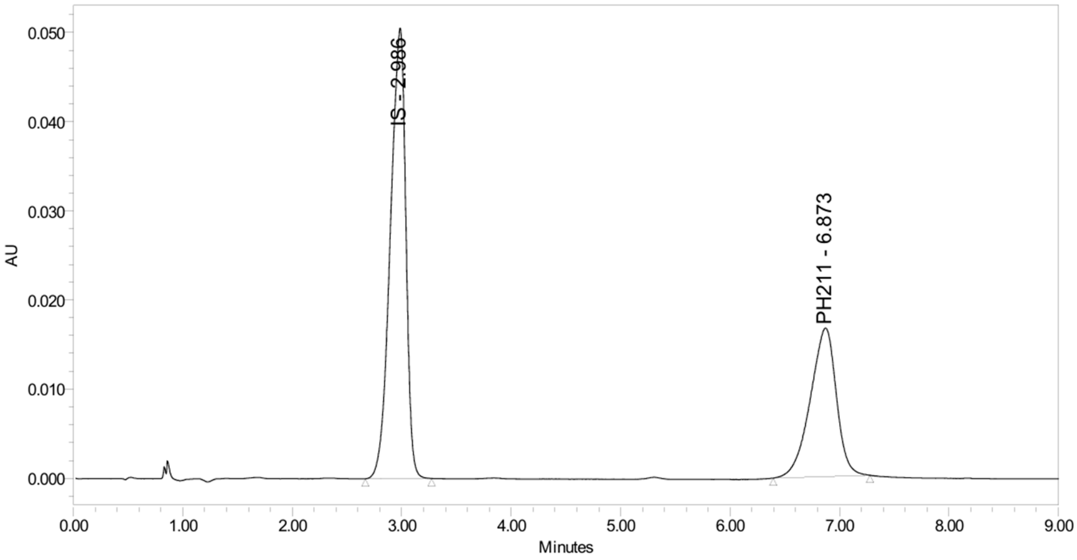

Figure S42: UHPLC-UV chromatogram of 0.01mg/mL of PH-211 and 0.1 mg/mL of the IS

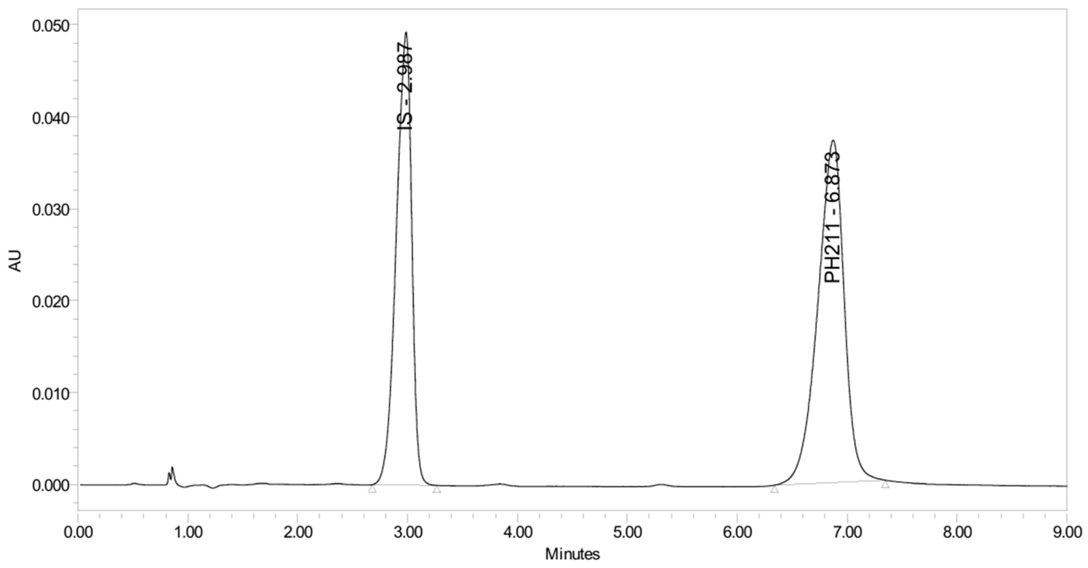

Figure S43: UHPLC-UV chromatogram of 0.02 mg/mL of PH-211 and 0.1 mg/mL of the IS

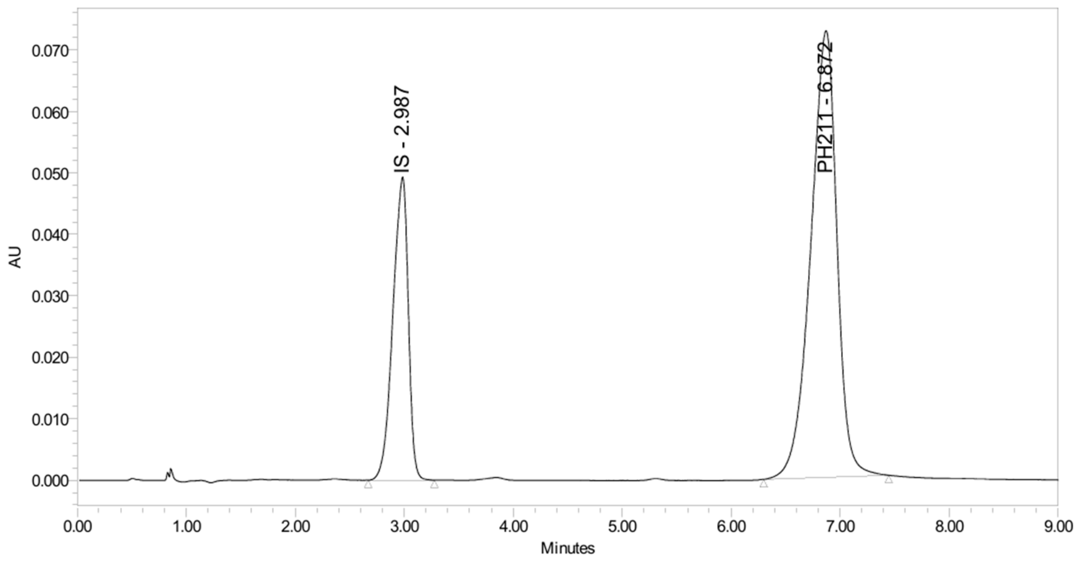

Figure S44: UHPLC-UV chromatogram of 0.04 mg/mL of PH-211 and 0.1 mg/mL of the IS

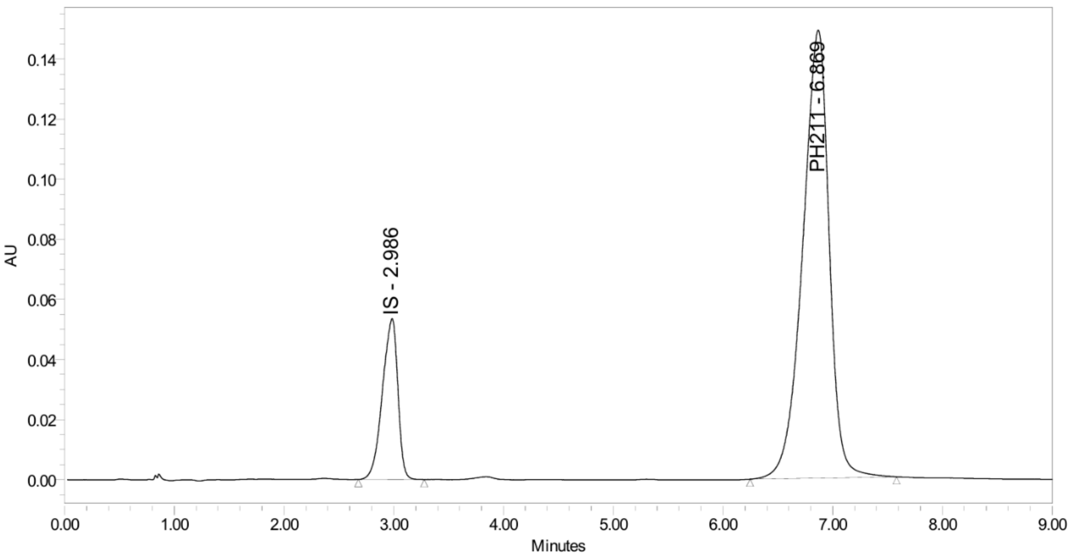

Figure S45: UHPLC-UV chromatogram of 0.08 mg/mL of PH-211 and 0.1 mg/mL of the IS

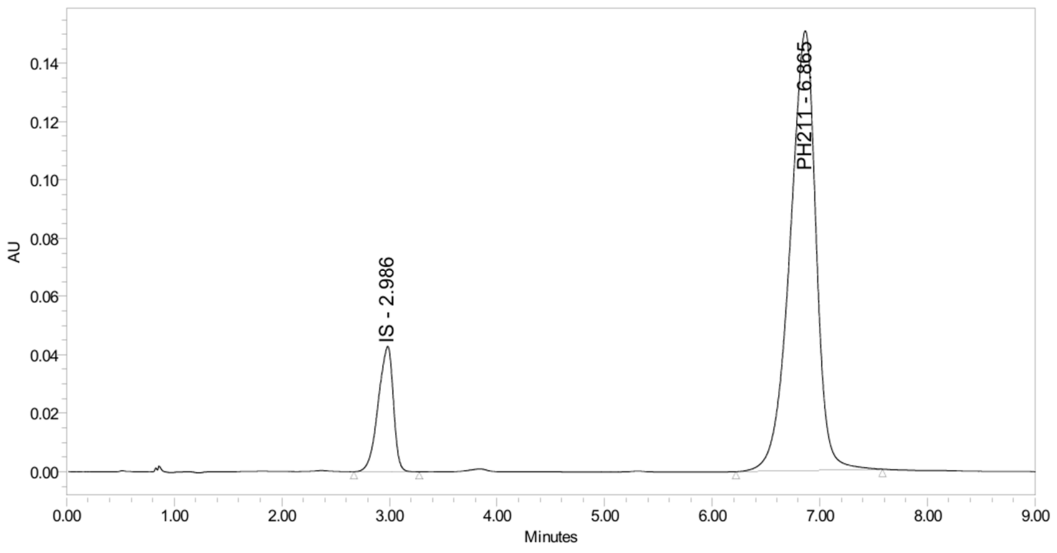

Figure S46: UHPLC-UV chromatogram of 0.1 mg/mL of PH-211 and 0.1 mg/mL of the IS

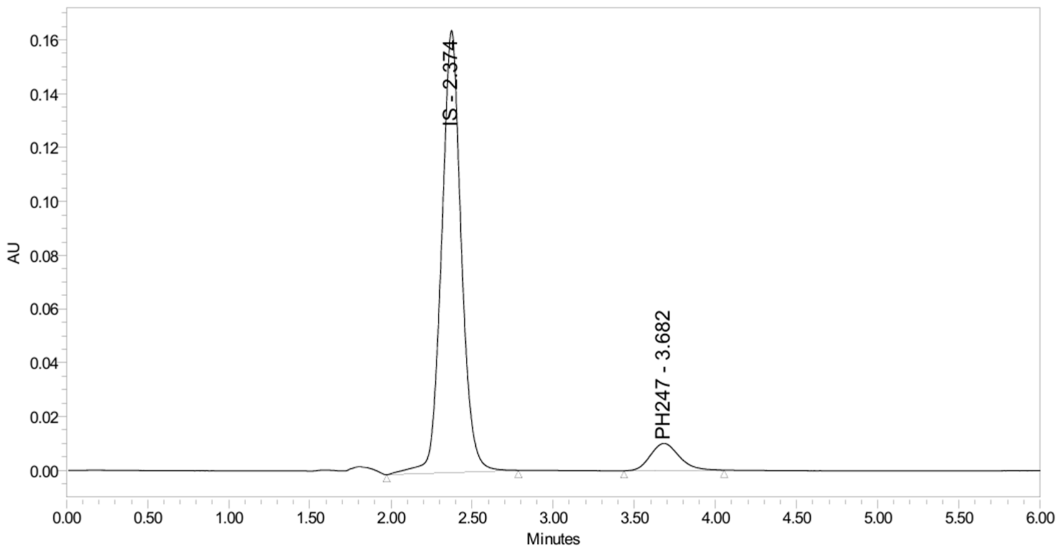

Figure S47: UHPLC-UV chromatogram of 0.005 mg/mL of PH-247 and 0.1 mg/mL of the IS

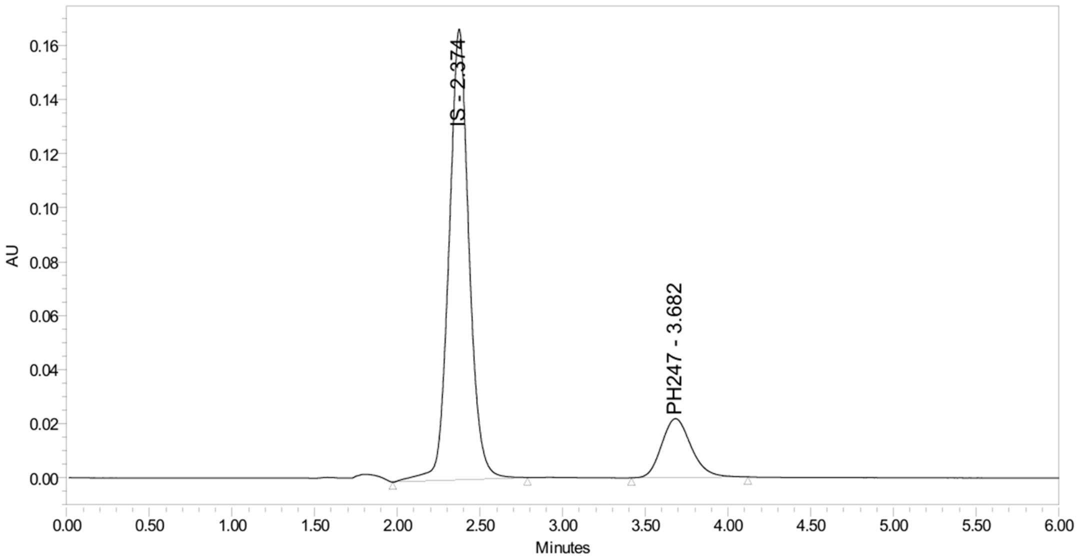

Figure S48: UHPLC-UV chromatogram of 0.01 mg/mL of PH-247 and 0.1 mg/mL of the IS

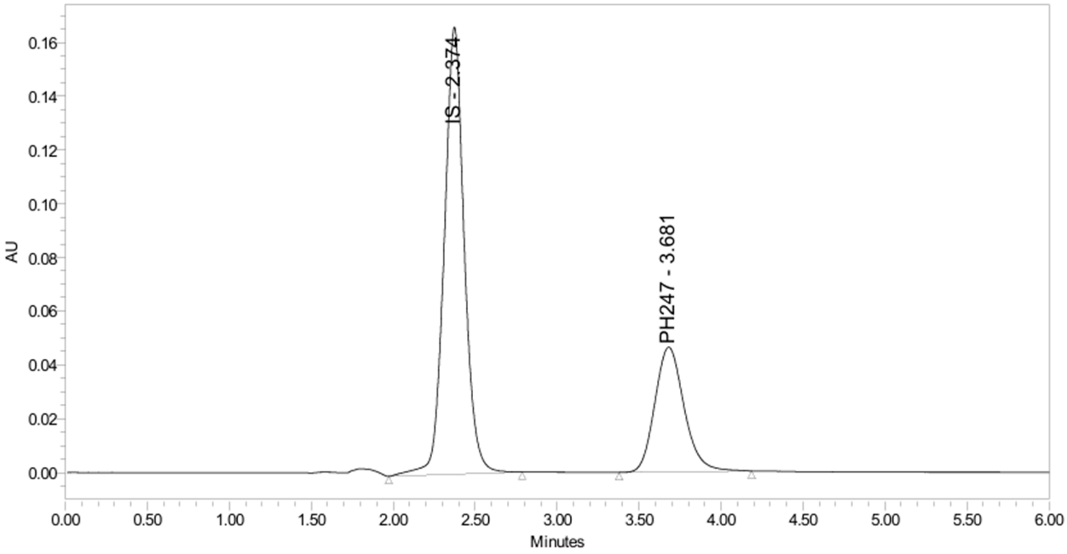

Figure S49: UHPLC-UV chromatogram of 0.02 mg/mL of PH-247 and 0.1 mg/mL of the IS

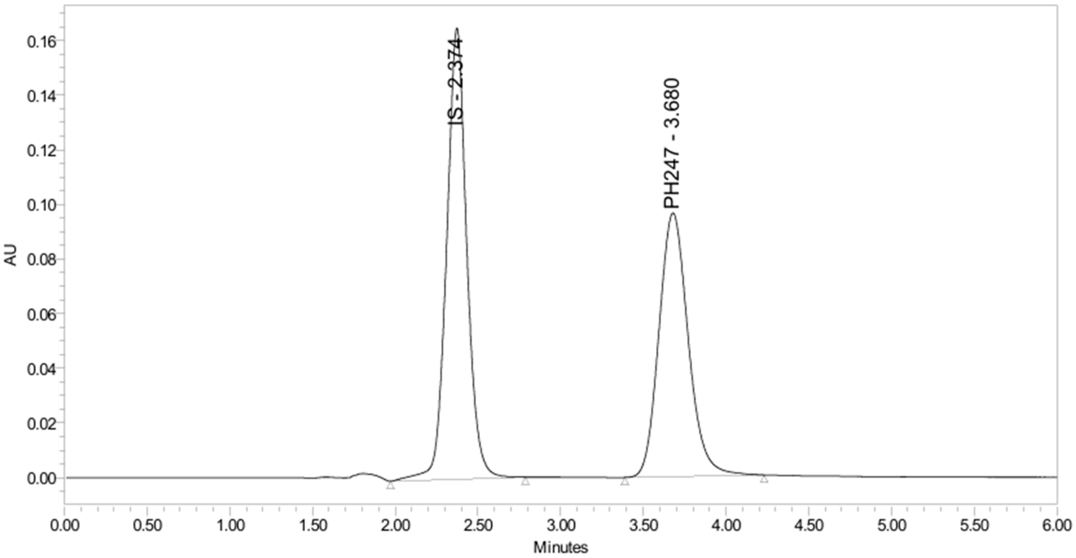

Figure S50: UHPLC-UV chromatogram of 0.04 mg/mL of PH-247 and 0.1 mg/mL of the IS

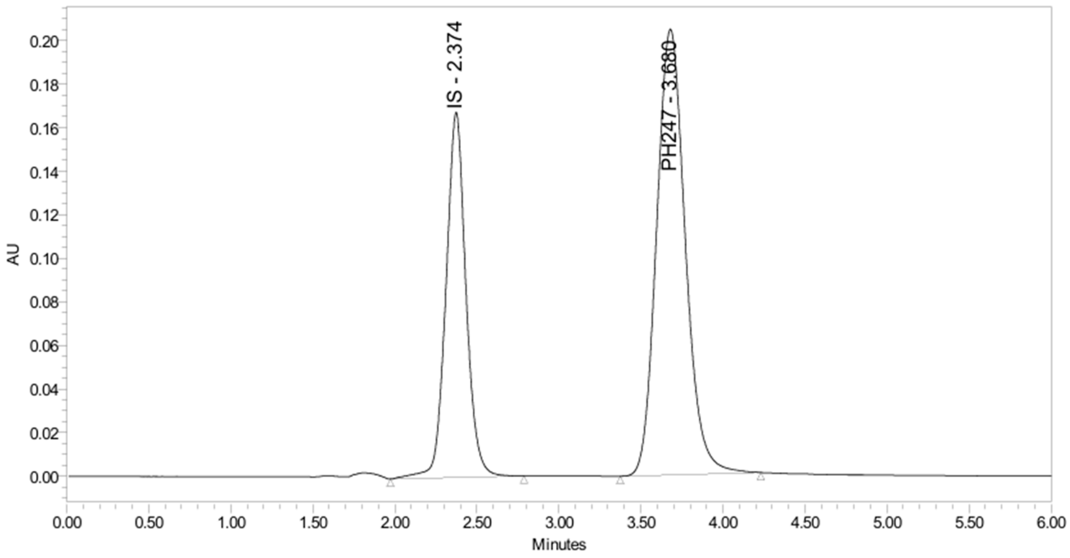

Figure S51: UHPLC-UV chromatogram of 0.08 mg/mL of PH-247 and 0.1 mg/mL of the IS

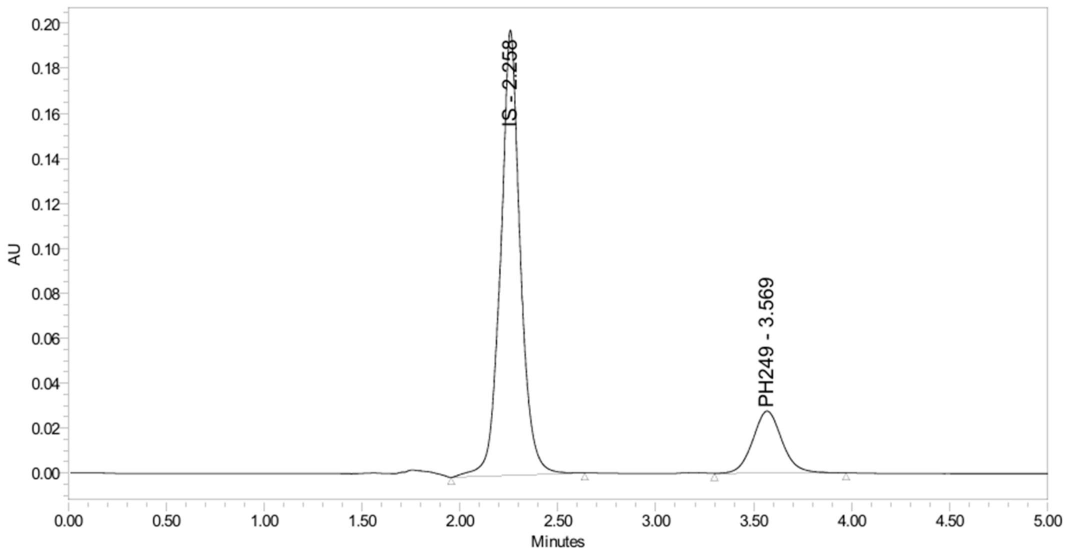

Figure S52: UHPLC-UV chromatogram of 0.01 mg/mL of PH-249 and 0.1 mg/mL of the IS

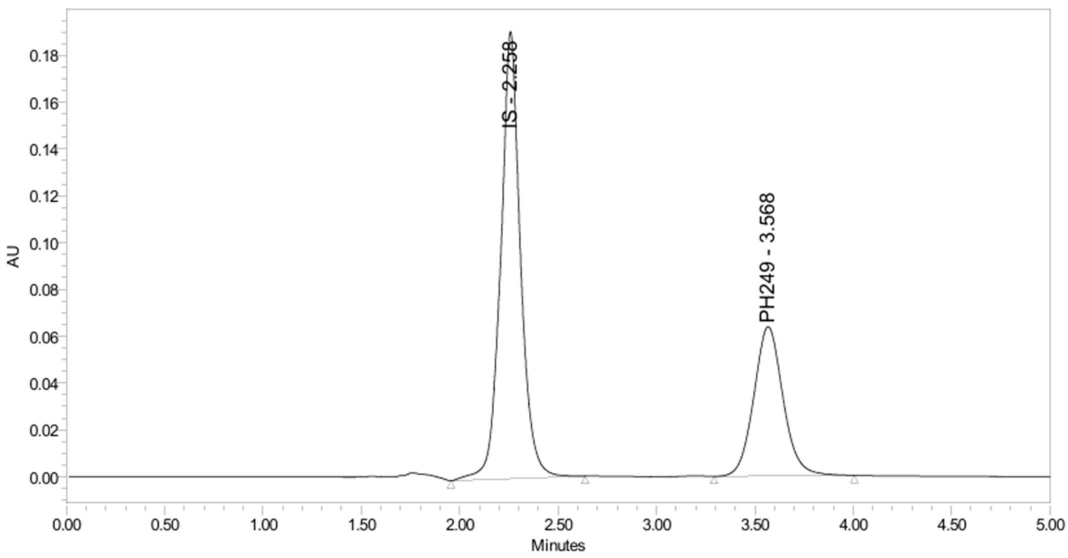

Figure S53: UHPLC-UV chromatogram of 0.02 mg/mL of PH-249 and 0.1 mg/mL of the IS

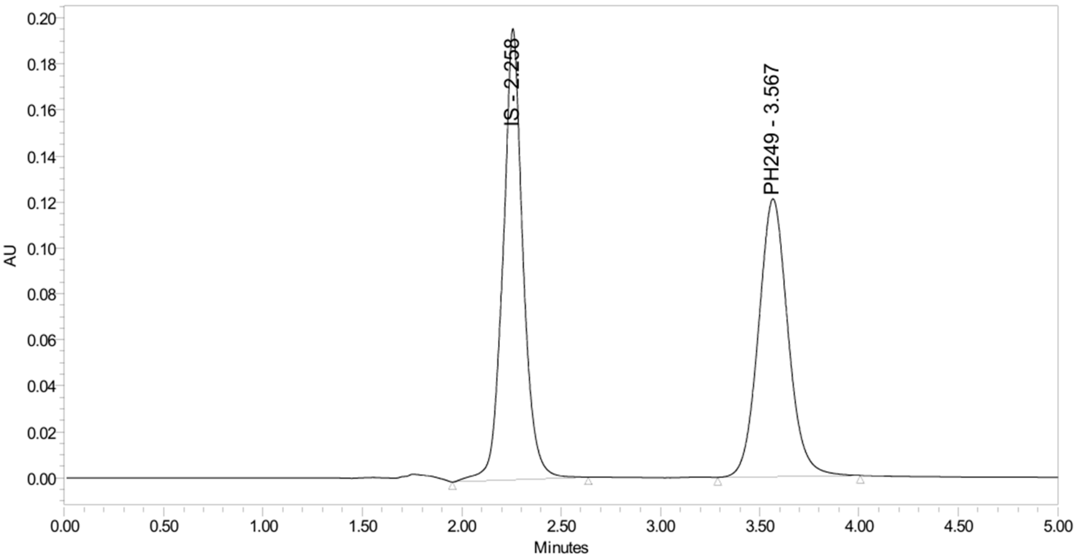

Figure S54: UHPLC-UV chromatogram of 0.04 mg/mL of PH-249 and 0.1 mg/mL of the IS

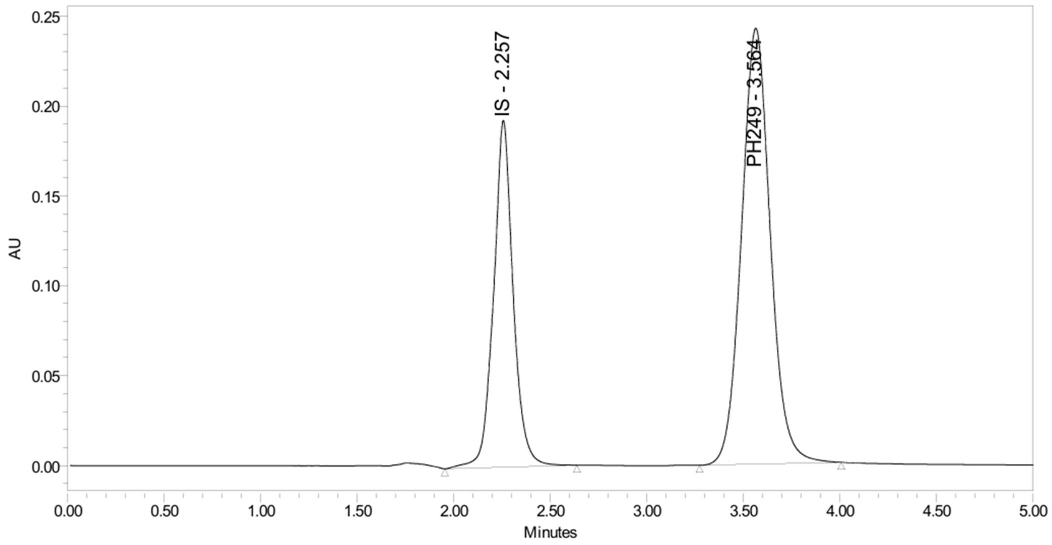

Figure S55: UHPLC-UV chromatogram of 0.08 mg/mL of PH-249 and 0.1 mg/mL of the IS

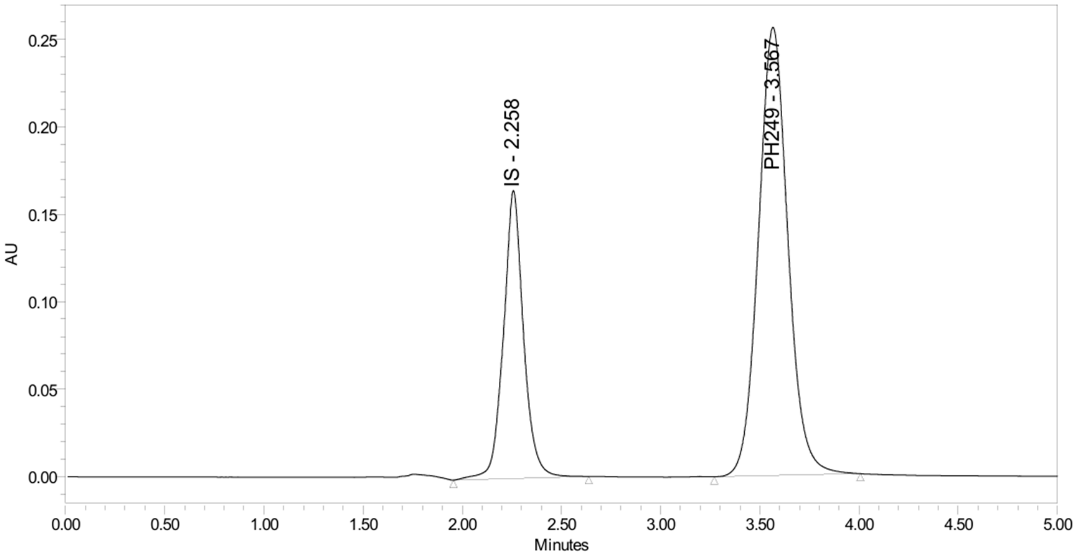

Figure S56: UHPLC-UV chromatogram of 0.1 mg/mL of PH-249 and 0.1 mg/mL of the IS

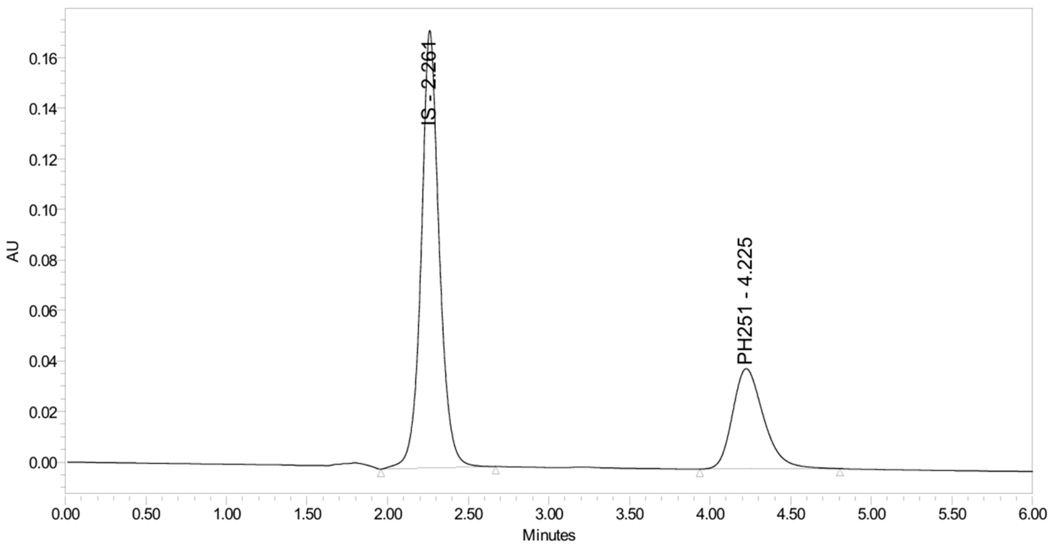

Figure S57: UHPLC-UV chromatogram of 0.01 mg/mL of PH-251 and 0.1 mg/mL of the IS

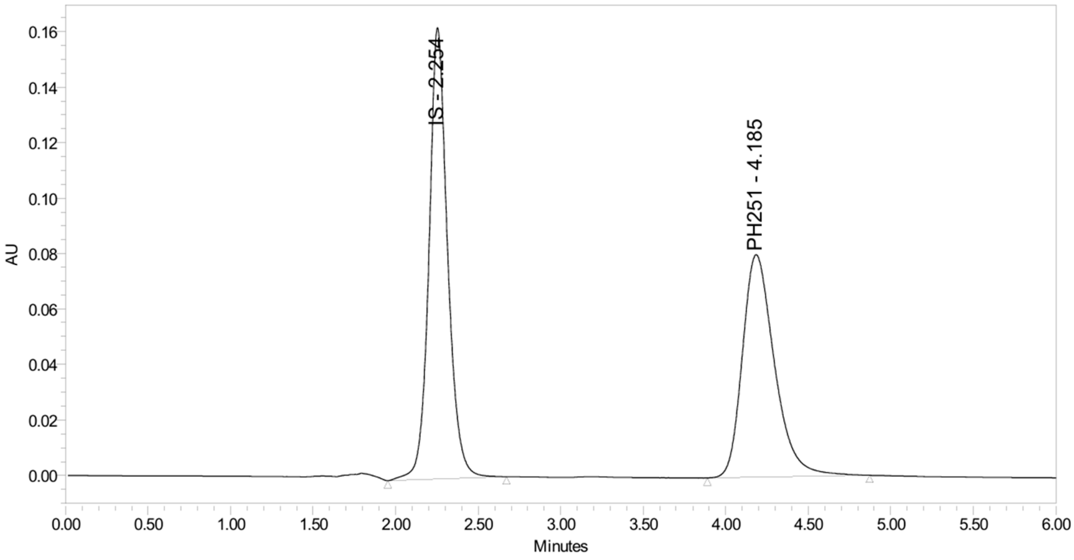

Figure S58: UHPLC-UV chromatogram of 0.02 mg/mL of PH-251 and 0.1 mg/mL of the IS

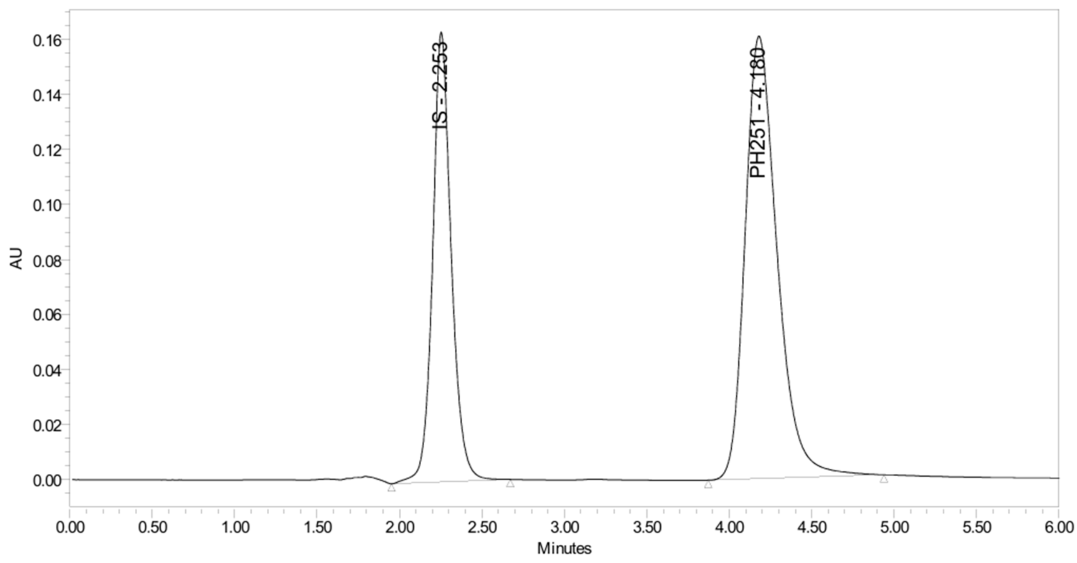

Figure S59: UHPLC-UV chromatogram of 0.04 mg/mL of PH-251 and 0.1 mg/mL of the IS

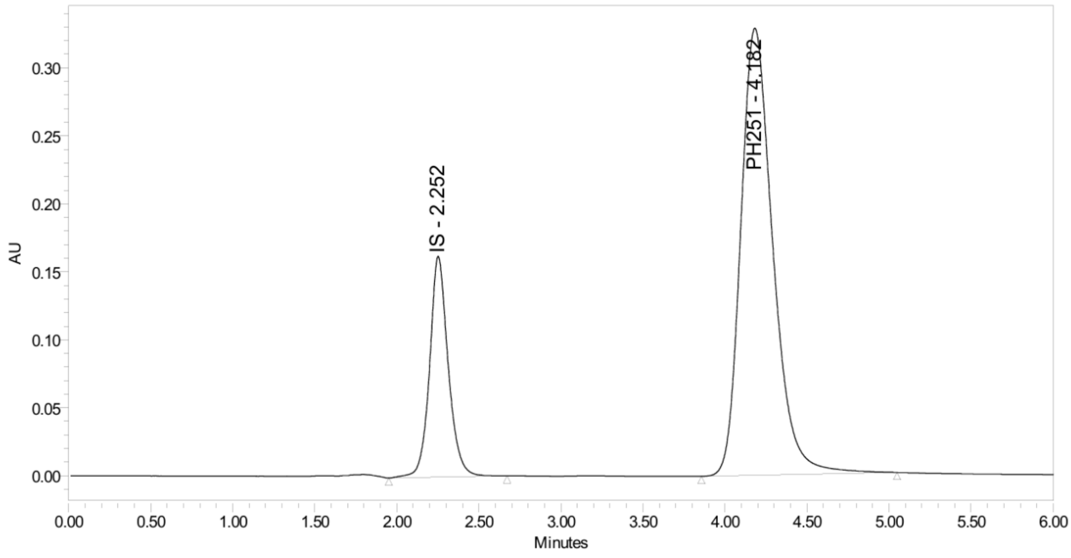

Figure S60: UHPLC-UV chromatogram of 0.08 mg/mL of PH-251 and 0.1 mg/mL of the IS

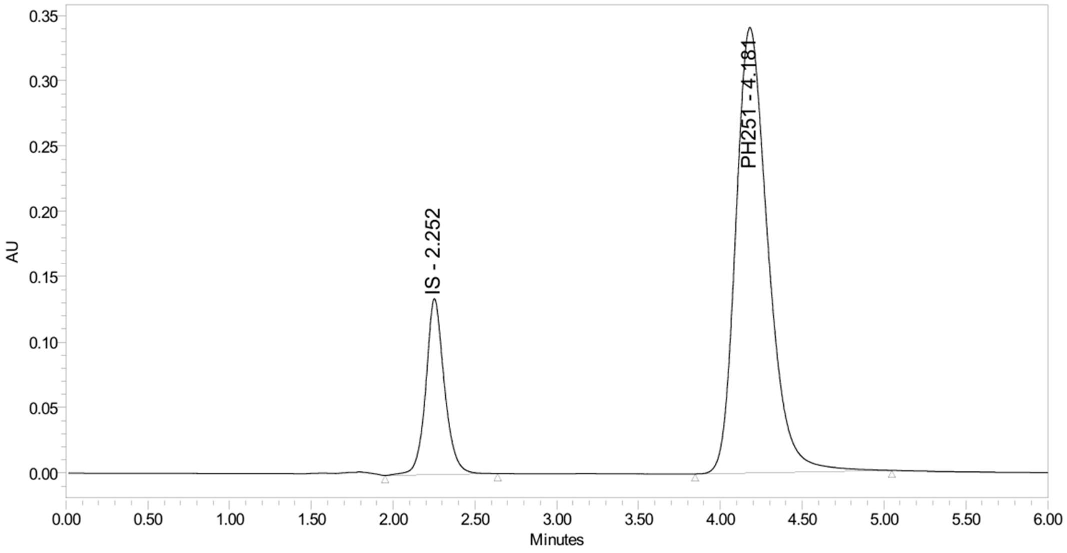

Figure S61: UHPLC-UV chromatogram of 0.1 mg/mL of PH-251 and 0.1 mg/mL of the IS

File S5 Calibration curves

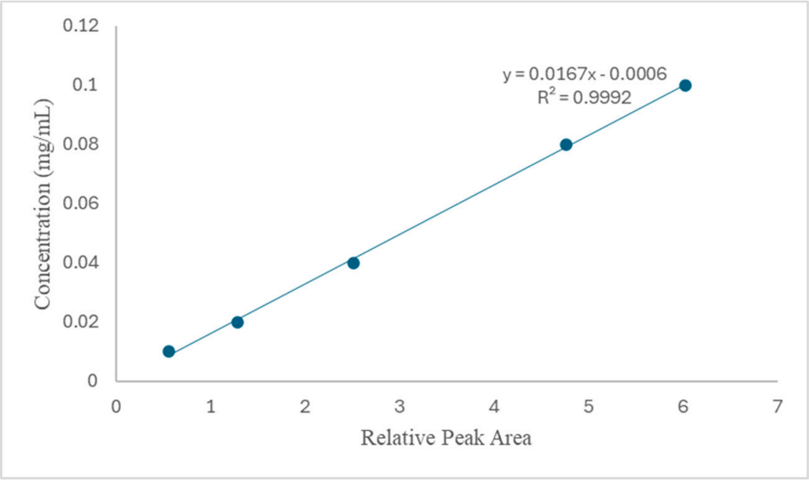

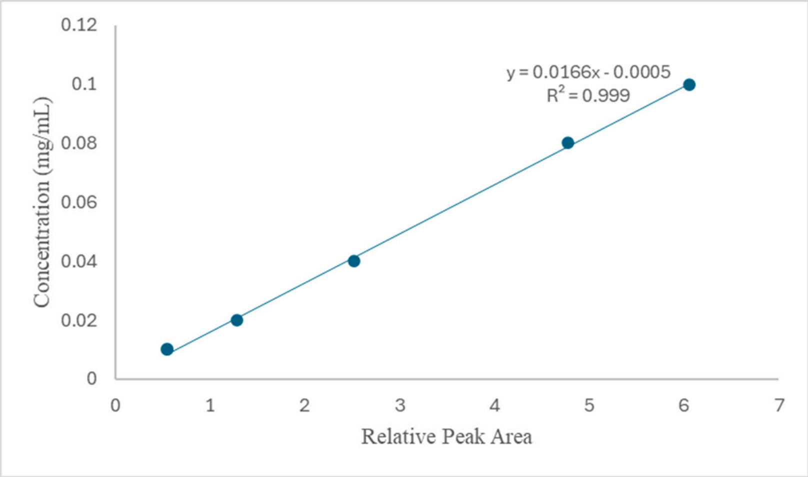

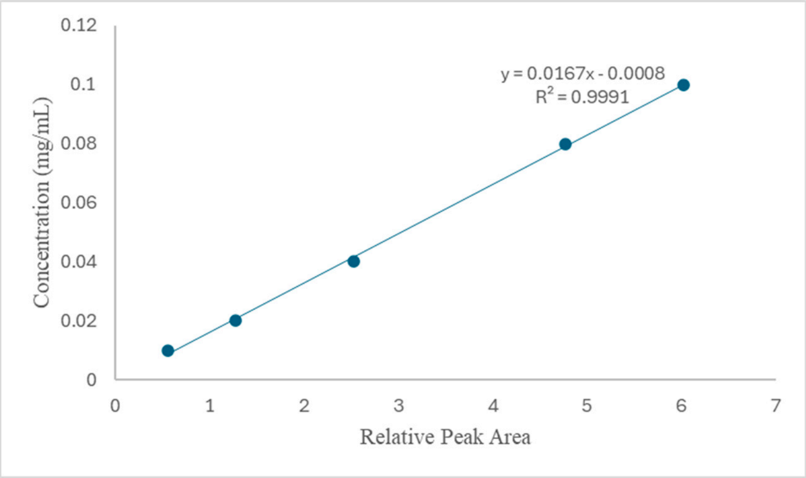

Figure S62: Calibration curves of PH-211

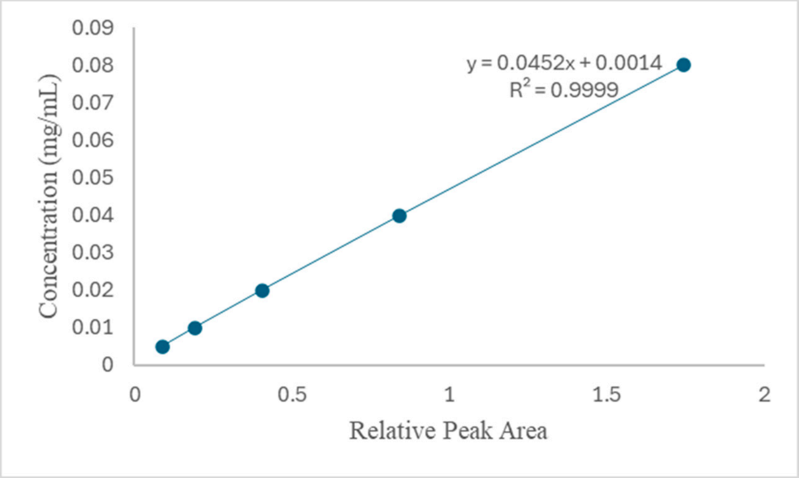

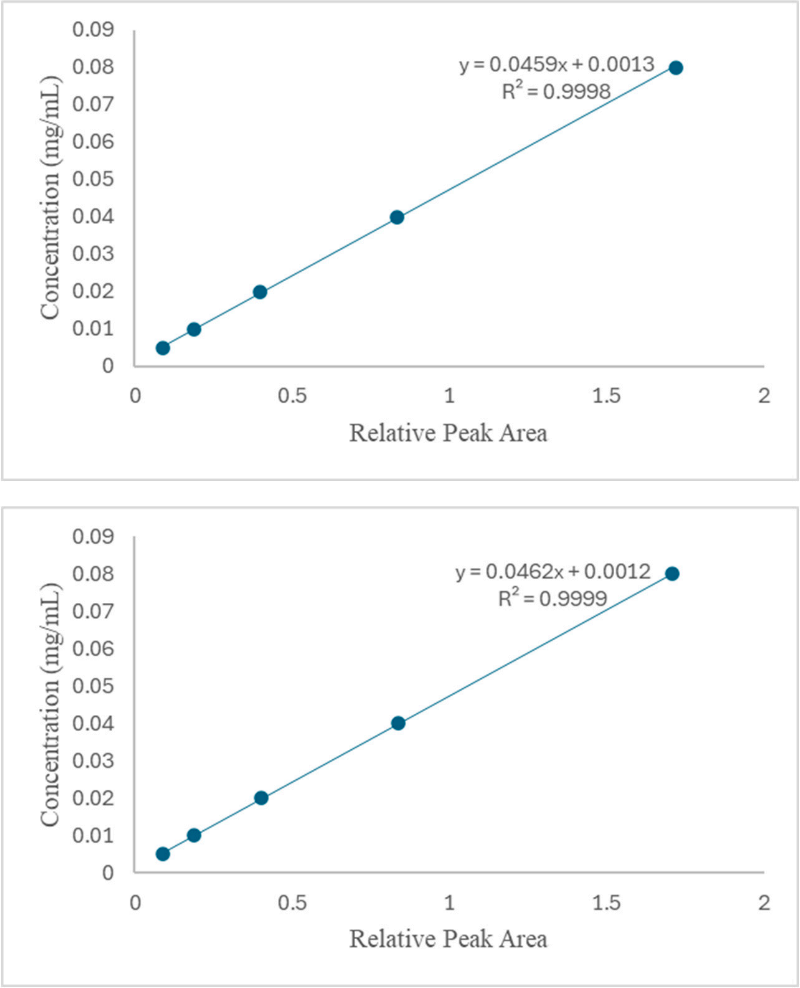

Figure S63: Calibration curves of PH-247

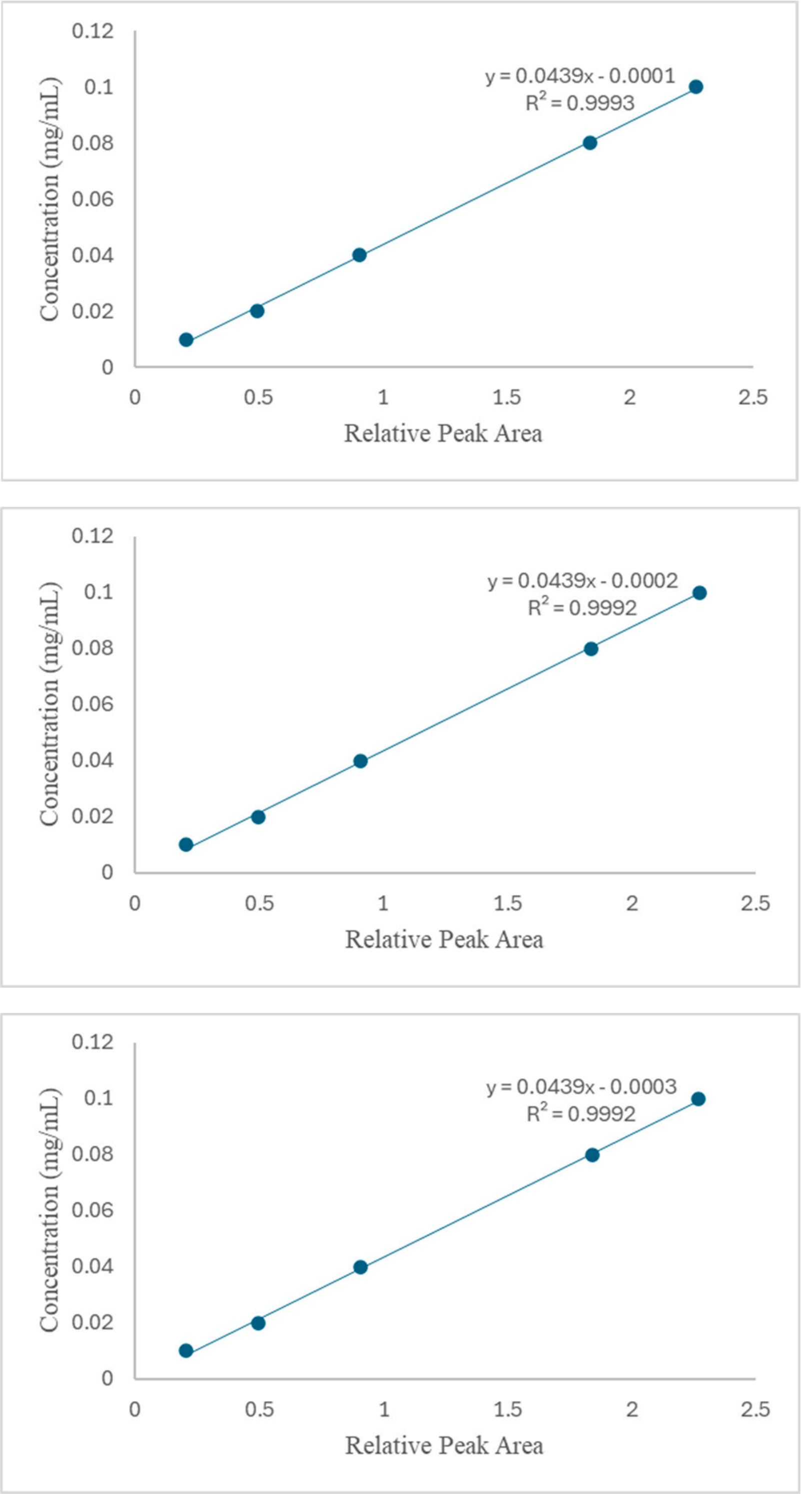

Figure S64: Calibration curves of PH-249

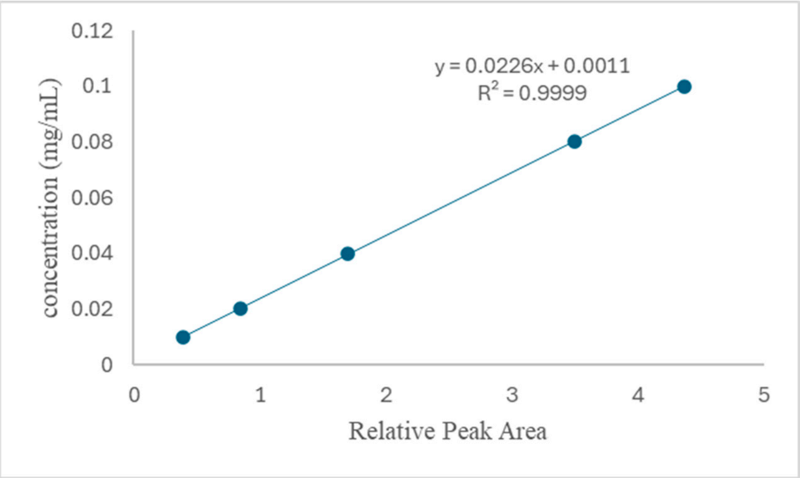
y = 0.0226x + 0.0011
R² = 0.9999
concentration (mg/mL)
Relative Peak Area
0.12
0.1
0.08
0.06
0.04
0.02
0
0 1 2 3 4 5

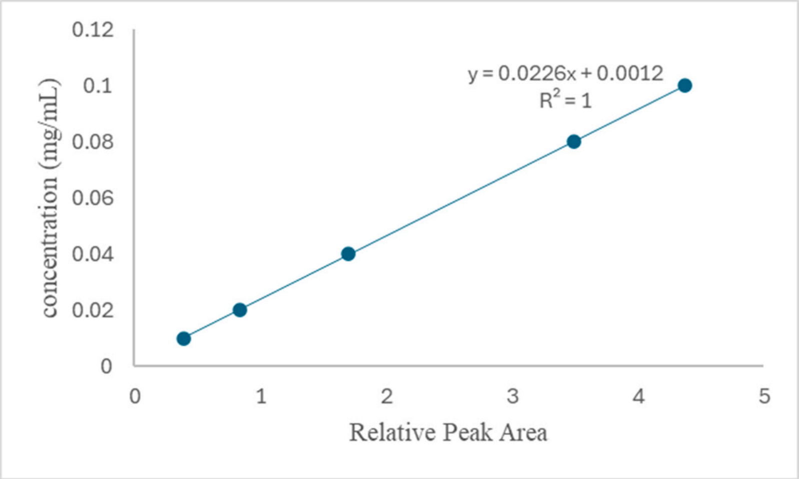
y = 0.0226x + 0.0012
R² = 1
concentration (mg/mL)
Relative Peak Area
0.12
0.1
0.08
0.06
0.04
0.02
0
0 1 2 3 4 5

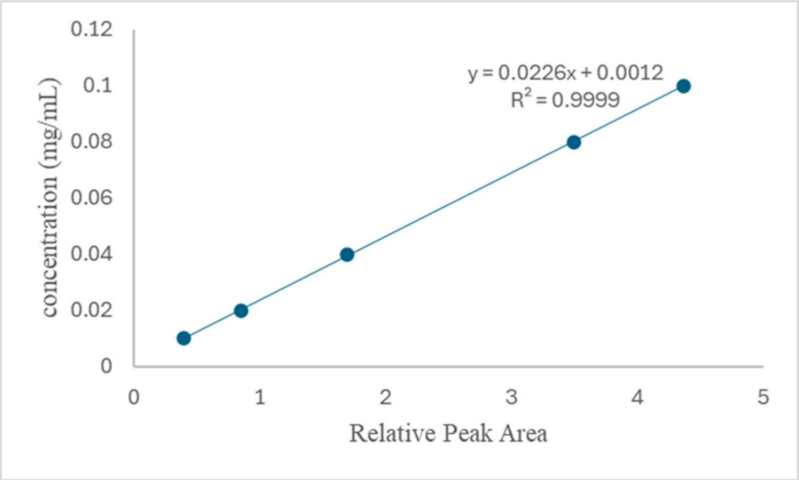
y = 0.0226x + 0.0012
R² = 0.9999
concentration (mg/mL)
Relative Peak Area
0.12
0.1
0.08
0.06
0.04
0.02
0
0 1 2 3 4 5

Figure S65: Calibration curves of PH-251

File S6 UHPLC-UV chromatograms of the plasma stability

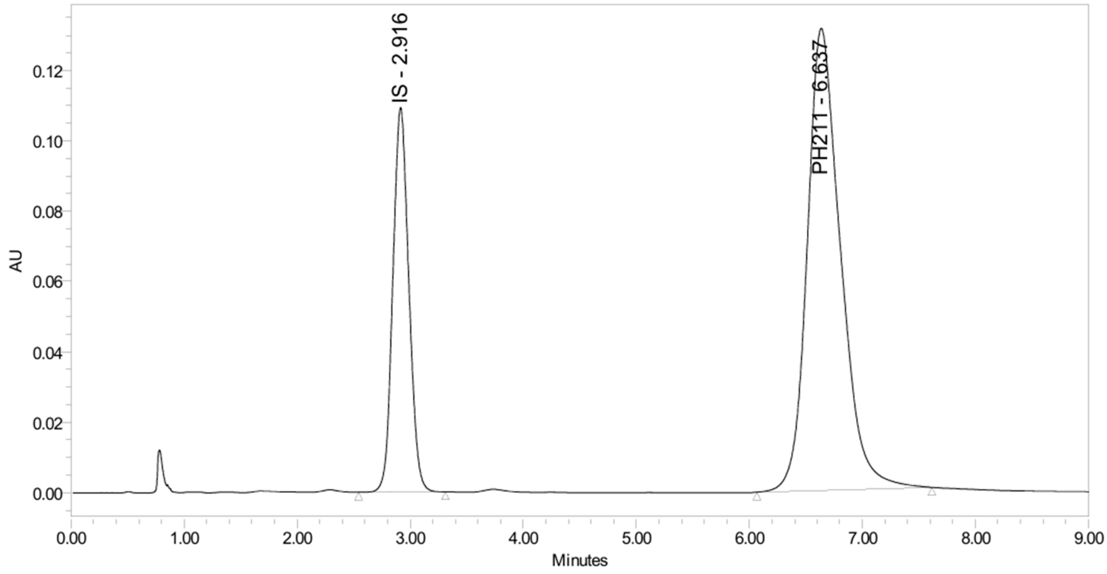

Figure S66: UHPLC-UV chromatogram of PH-211 plasma stability

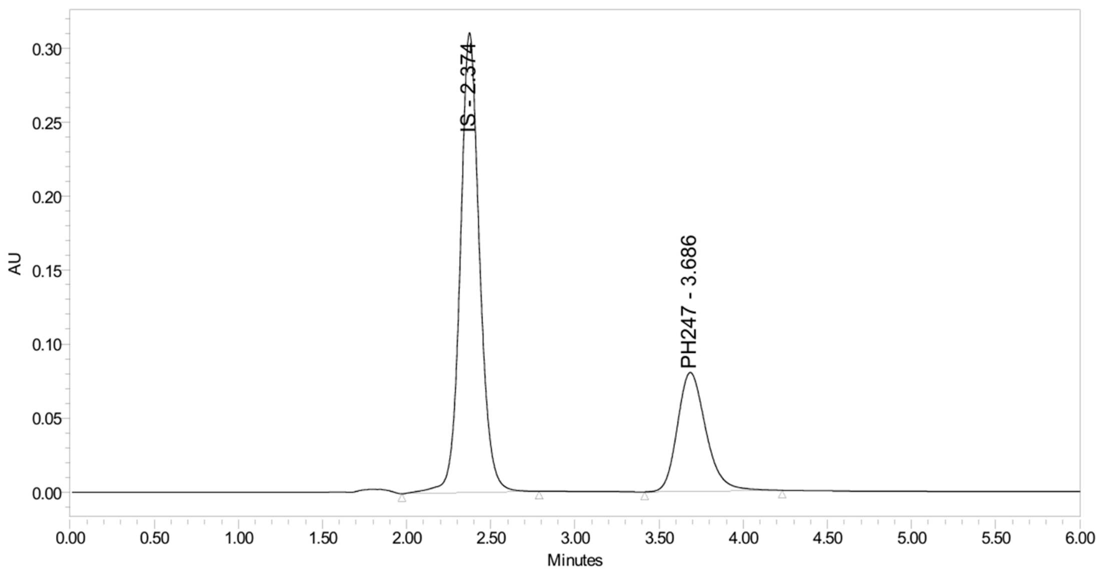

Figure S67: UHPLC-UV chromatogram of PH-247 plasma stability

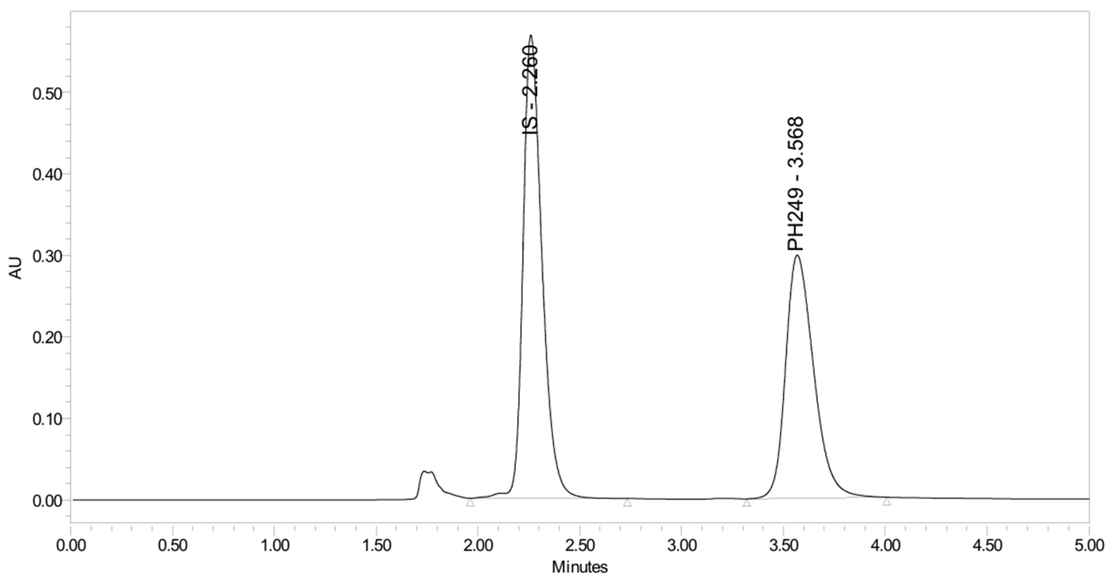

Figure S68: UHPLC-UV chromatogram of PH-249 plasma stability

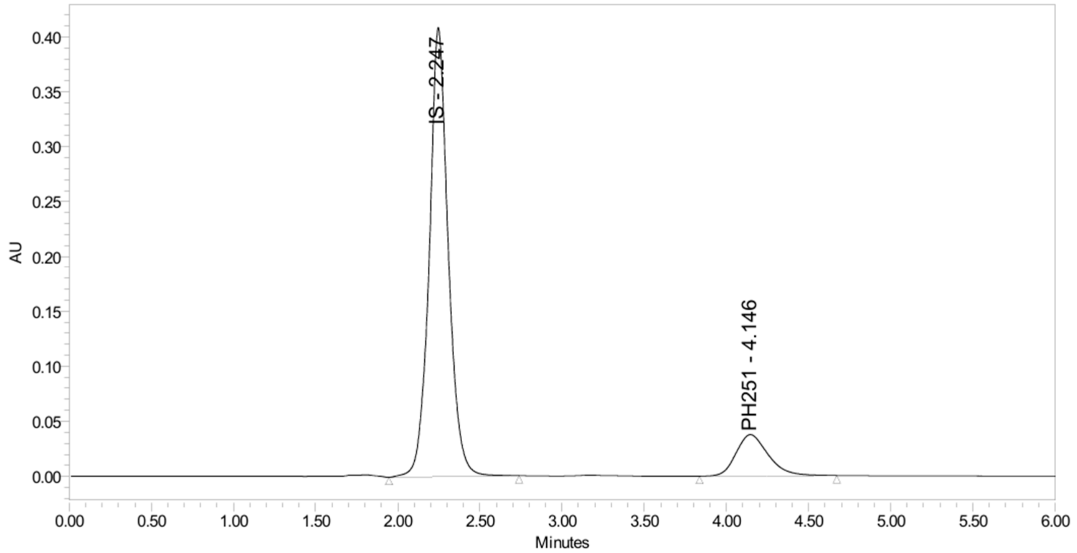

Figure S69: UHPLC-UV chromatogram of PH-251 plasma stability
